# Practical and Efficient Synthesis of (*E*)-α,β-Unsaturated Amides Incorporating α-Aminophosphonates via the Horner–Wadsworth–Emmons Reaction

**DOI:** 10.3390/molecules30183730

**Published:** 2025-09-13

**Authors:** Sindy Anahi Perez-Aniceto, Erica Cano-Tapia, Mario Ordoñez, José Luis Viveros-Ceballos, Ivan Romero-Estudillo

**Affiliations:** 1 Centro de Investigaciones Químicas-IICBA, Universidad Autónoma del Estado de Morelos, Av. Universidad 1001, Cuernavaca 62209, Morelos, Mexico; sindy.perez@uaem.edu.mx (S.A.P.-A.); erika.canotap@uaem.edu.mx (E.C.-T.); 2 Secihti-Centro de Investigaciones Químicas-IICBA, Universidad Autónoma del Estado de Morelos, Av. Universidad 1001, Cuernavaca 62209, Morelos, Mexico; ivan.romeroest@uaem.mx

**Keywords:** α-aminophosphonates, multicomponent reactions, α,β-unsaturated amides, Horner-Wadsworth-Emmons reaction, cinnamic acid derivatives

## Abstract

An efficient and practical procedure for the synthesis of (*E*)-α,β-unsaturated amides incorporating α-aminophosphonates, derived from readily accessible phosphonoacetamides, via the Horner–Wadsworth–Emmons (HWE) reaction was developed. The influence of reaction parameters, including base, solvent, and temperature, as well as the scope of the method with different aldehydes, was examined, affording the target compounds in good yields and with high (*E*)-selectivity. The required phosphonoacetamides were conveniently prepared through a Kabachnik–Fields reaction of aldehydes, benzylamine and dimethyl phosphite followed by hydrogenolytic cleavage of the *N*-Bn bond, acylation with bromoacetyl bromide, and a subsequent Arbuzov reaction. This HWE protocol provides straightforward access to a broad range of (*E*)-α,β-unsaturated amides incorporating α-aminophosphonates under mild conditions, offering valuable scaffolds with potential pharmacological relevance as anticancer agents.

## 1. Introduction

**Figure 1:**
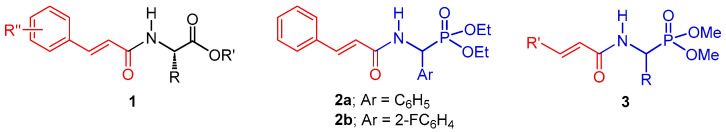
(*E*)-α,β-unsaturated amides incorporating α-amino acids and α-aminophosphonates with biological properties.

Cinnamic acid and derivatives, both isolated from natural sources and by synthetic methods, are important compounds characterized by low toxicity and a broad spectrum of biological activities and chemical applications [1,2]. Among them, cinnamic acid amides exhibit a wide range of biological activities [3,4], mainly those that incorporate an α-amino acid moiety in their structure **1** (Figure 1), which have been evaluated as anticancer agents [5], antimalarial [6], antioxidant [7,8], and as agents for the treatment of Alzheimer’s disease [9].

On the other hand, the α-aminophosphonic and α-aminophosphonate analogues of the corresponding α-amino acids and α-amino esters **2** and **3** (Figure 1), respectively, represent an important class of compounds with multiple applications in medicinal and organic chemistry, due to the ability of the phosphonic group to mimic the high-energy transition state of peptide bond hydrolysis, enabling them to act as enzyme inhibitors or receptor ligands in pathological conditions associated with α-amino acid metabolism [10,11,12,13,14]. Due to the importance of these compounds as a bioisosters of carboxylates, excellent methods for their preparation have been published in recent decades [15,16,17,18,19,20].

**Scheme 1:**
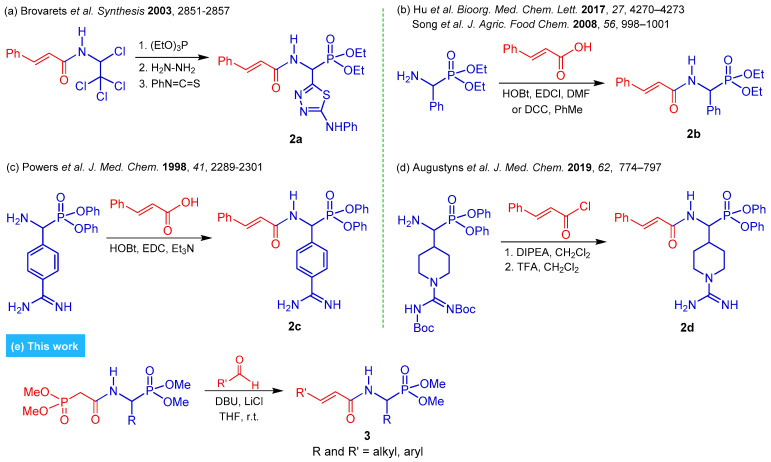
Methods for the synthesis of (*E*)-α,β-unsaturated amides incorporating α-aminophosphonates [21,22,23,24,25,26,27].

In contrast to the different methods for the synthesis of (*E*)-α,β-unsaturated amides bearing α-amino acids, the (*E*)-α,β-unsaturated amides incorporating α-aminophosphonates has been little explored, and there are few reports has been described in the literature, mainly α-aminophosphonates **2a**–**d** carrying the cinnamic and acrylic acid fragment (Scheme 1a–d) [21,22,23,24,25,26,27]. Considering the importance of these non-proteinogenic α-aminophosphonic acids as pharmacophores in the discovery of new drugs, which is in line with our current research interest in the synthesis of α-aminophosphonic acids [28], we herein report an alternative and practical method for the preparation of α,β-unsaturated amides **3** bearing α-aminophosphonates via the Horner–Wadsworth–Emmons reaction using phosphonoacetamides **7a**–**c** (Scheme 1e).

## 2. Results and Discussion

**Scheme 2:**
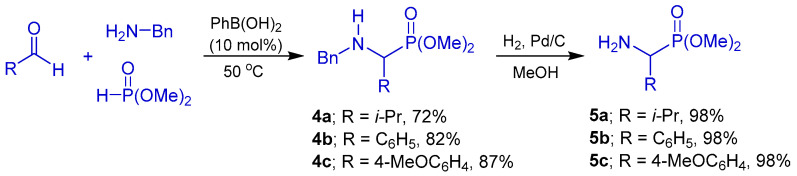
Synthesis of α-aminophosphonates **4a**–**c** and **5a**–**c**.

For the preparation of (*E*)-α,β-unsaturated amides incorporating α-aminophosphonates, initially we carried out the synthesis of α-aminophosphonates **5a**–**c** through the Kabachnik–Fields reaction [29,30] of isobutyraldehyde, benzaldehyde, or 4-methoxybenzaldehyde with benzylamine and dimethyl phosphite in the presence of 10 mol% of PhB(OH)_2_ at 50 °C under solvent-free conditions [31], obtaining the corresponding *N*-benzyl α-aminophosphonates **4a**–**c** in 72 to 87% yield. Subsequent debenzylation over Pd/C in methanol under hydrogen pressure using a balloon, afforded the α-aminophosphonates **5a**–**c** in 98% yield, which were used in the following reactions without further purification (Scheme 2).

With the α-aminophosphonates **5a**–**c** in hand, we decided to explore the preparation of (*E*)-α,β-unsaturated amide **3a** incorporating the α-aminophosphonate **5a** moiety through the reaction of **5a** with *trans*-cinnamic acid, using several coupling reagents, such as acyl chloride, hexafluorophosphate benzotriazole tetramethyl uronium (HBTU), benzotriazol-1-yl-oxy-tris (dimethylamino) phosphonium hexafluorophosphate (PyBOP), *N*,*N*’-Dicyclohexylcarbodiimide (DCC) and mixed anhydrides [*i*-BuOC(O)Cl, (Boc)_2_O]. In this context, initially the *trans*-cinnamic acid was reacted with SOCl_2_ and *N*,*N*-diisopropylethylamine (DIPEA) in dichloromethane at 0 to 25 °C to obtain the corresponding cinnamoyl chloride which, without isolation was reacted with the α-aminophosphonate **5a**, obtaining the (*E*)-α,β-unsaturated amide **3a** in 56% yield (Table 1, entry 1). In the next experiment, the *trans*-cinnamic acid was activated with HBTU and DIPEA in dichloromethane, to give the compound **3a** in 76% yield (Table 1, entry 2). Similar results were obtained when PyBOP was used as the coupling reagent and DIPEA in MeCN at 0 to 25 °C (Table 1, entry 3). Additionally, we carried out the reaction of *trans*-cinnamic acid with DCC and 4-dimethylaminopyridine (DMAP) in dichloromethane followed by the addition of **5a**, affording the (*E*)-α,β-unsaturated amide **3a** in 74% (Table 1, entry 4). Similar results were obtained when the mixed anhydride derived from *trans*-cinnamic acid and isobutyl chloroformate was reacted with the α-aminophosphonate **5a** (Table 1, entry 5). Finally, the reaction of **5a** with the mixed anhydride obtained from *trans*-cinnamic acid and di-*tert*-butyl dicarbonate [(Boc)_2_O], produced (*E*)-α,β-unsaturated amide **3a** in 68% (Table 1, entry 6).

**Scheme 3:**
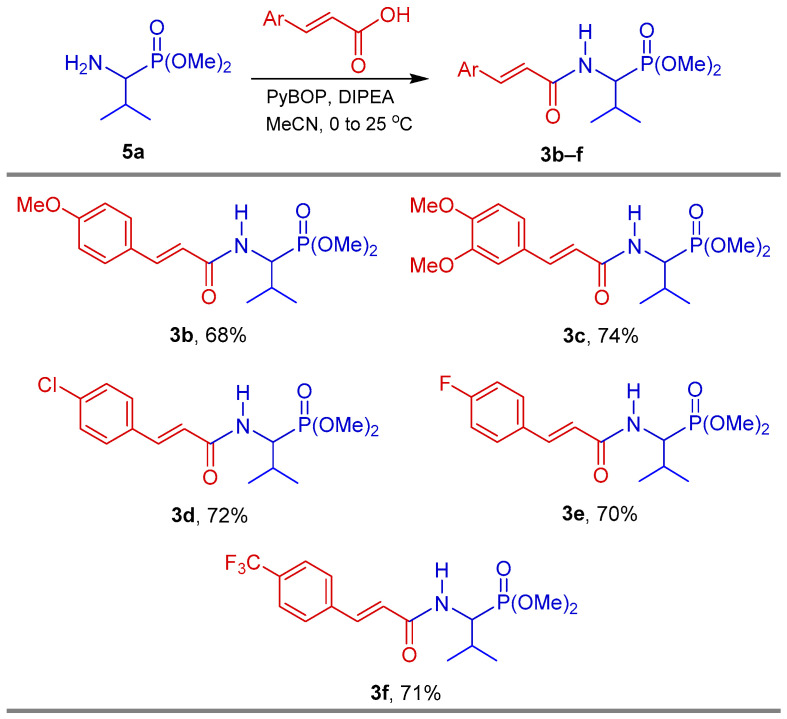
Synthesis of (*E*)-α,β-unsaturated amides **3b**–**f** incorporating the α-aminophosphonate **5a**.

With the optimized reaction conditions established for the synthesis of (*E*)-α,β-unsaturated amide **3a**, we turned our attention to investigate the scope and versatility of this method in the synthesis of several (*E*)-α,β-unsaturated amides incorporating the α-aminophosphonate **5a**. Thus, the scope of this reaction was evaluated by reacting the α-aminophosphonate **5a** with several cinnamic acids, using PyBOP as the coupling agent and DIPEA in MeCN at 0 to 25 °C, obtaining the corresponding (*E*)-α,β-unsaturated amides **3b**–**f** in 68 to 74% yield (Scheme 3).

**Scheme 4:**
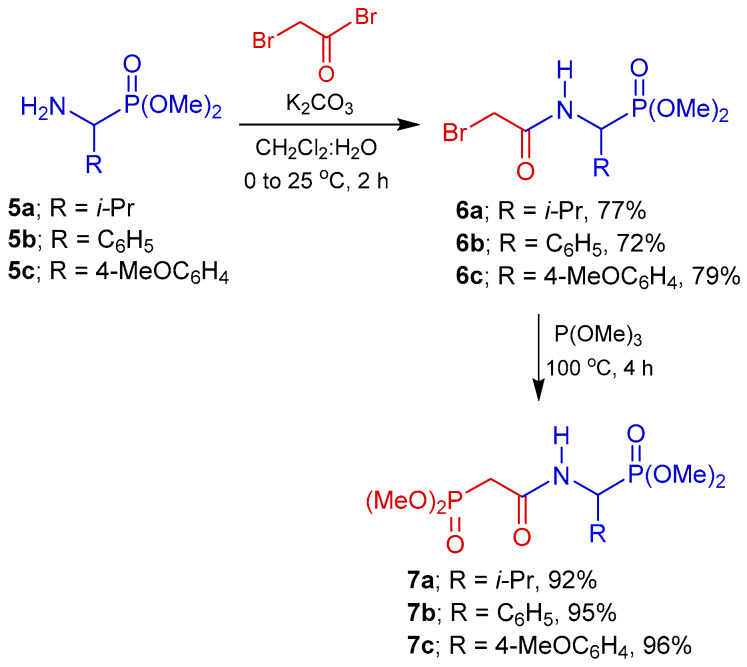
Synthesis of phosphonoacetamides **7a**–**c**.

Although the method described above produced the desired (*E*)-α,β-unsaturated amides in good yields, an important limitation is the limited commercial availability of the corresponding acrylic acid and derivatives. Therefore, we decided to explore a more general method to access (*E*)-α,β-unsaturated amides bearing aryl or alkyl substituents at the β-position, via the Horner–Wadsworth–Emmons (HWE) reaction, a method with which we have previous experience [32,33]. To accomplish the above, it was necessary to prepare the phosphonoacetamides **7a**–**c**, our starting materials. Thus, the α-aminophosphonates **5a**–**c** were reacted with bromoacetyl bromide and potassium carbonate in a dichloromethane:water mixture (4:1) at room temperature, producing the bromoamides **6a**–**c** in 72 to 79% yield, which were subjected to a Michaelis-Arbuzov reaction with trimethyl phosphite at 100 °C, to obtain the corresponding phosphonoacetamides **7a**–**c** in 92 to 96% yield (Scheme 4).

With the phosphonoacetamides **7a**–**c** successfully prepared, the next step was to identify suitable conditions for the preparation of the target α,β-unsaturated amides bearing an α-aminophosphonate moiety through the Horner–Wadsworth–Emmons reaction. Initially, the phosphonoacetamide **7a** was reacted with cesium carbonate and benzaldehyde in acetonitrile at 50 °C for 12 h, obtaining the α,β-unsaturated amide **3a** in 72% yield with an *E*:*Z* ratio of 93:07 (Table 2, entry 1). In a second attempt, the reaction was conducted at 80 °C for 6 h, producing **3a** in 70% yield and with similar *E-*selectivity 92:08 (Table 2, entry 2). When the reaction was carried out in toluene at 100 °C, the α,β-unsaturated amide **3a** was obtained in 65% yield and 93:07 *E:Z* selectivity (Table 2, entry 3). Subsequently, the reaction of phosphonoacetamide **7a** with benzaldehyde and potassium carbonate in acetonitrile at 50 and 80 °C for 24 and 12 h, respectively, afforded the α,β-unsaturated amide **3a** in 63 and 68% yield and *E:Z* ratios of 91:09 and 87:13 (Table 2, entries 4 and 5). Performing the same reaction in toluene at 100 °C generated the α,β-unsaturated amide **3a** in 68% yield and *E:Z* ratio 61:39 (Table 2, entry 6). Finally, following the protocol developed by our research group [32,33], the phosphonoacetamide **7a** was reacted with benzaldehyde, 1,8-diazabicyclo[5,4,0]undec-7-ene (DBU) and LiCl in THF at 25 °C [34], obtaining the α,β-unsaturated amide **3a** in 90% yield with high *E*-selectivity 94:06 (Table 2, entry 7). The stereochemistry in the C=C double bond of the α-unsaturated amide **3a** was assigned on the basis of the value of ^1^H NMR coupling constant between the olefinic protons.

**Scheme 5:**
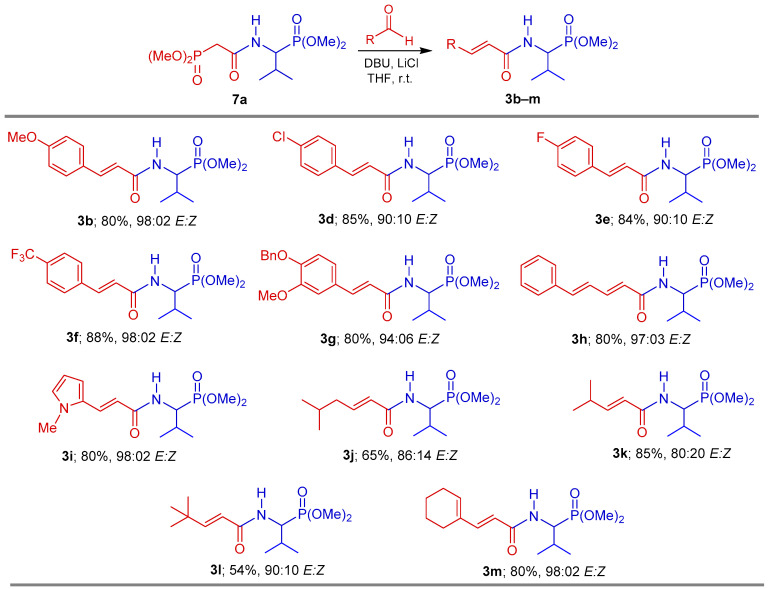
HWE reaction for the synthesis of α,β-unsaturated amides **3b**–**m**.

As shown in Table 2, the use of Cs_2_CO_3_, K_2_CO_3_ and DBU at room temperature afforded the α,β-unsaturated amide **3a** with high *E*-selectivity; however, the highest yield was achieved when DBU is used as the base. For this reason, we turned our attention to exploring the scope and versatility of this method in the synthesis of the α,β-unsaturated amides **3b** and **3d**–**l** incorporating dimethyl phosphovalinate via the Horner–Wadsworth–Emmons reaction using DBU as the most appropriate base. In this context, the phosphonoacetamide **7a** was reacted under HWE reaction with different aromatic aldehydes such as *4*-methoxybenzaldehyde, *4*-chlorobenzaldehyde, *4*-fluorobenzaldehyde, *4*-trifluoromethylbenzaldehyde, *O*-benzylvanillin, *trans*-cinnamaldehyde and *N*-methyl-2-pyrrolecarboxaldehyde, in the presence of DBU and lithium chloride in anhydrous THF at room temperature, obtaining the α,β-unsaturated amides incorporating the dimethyl phosphovalinate (*E*)-**3b** and **3d**–**i** in 80% to 88% and *E:Z* selectivities ranging from 90:10 to 98:02. It is worth noting that the electronic nature of the substituents on the aromatic ring had no significant effect on *E:Z* selectivity. Similarly, the reaction of phosphonoacetamide **7a** with aliphatic aldehydes such as isovaleraldehyde, isobutyraldehyde, trimethylacetaldehyde and 1-cyclohexenecarboxaldehyde under the same conditions, gave the α,β-unsaturated amides **3j**–**m** in 54 to 85% yield and *E:Z* selectivities ranging from 80:20 to 98:02. The *E:Z* ratio was determined by ^1^H NMR in the crude product and isolated yields after purification by column chromatography (Scheme 5).

**Scheme 6:**
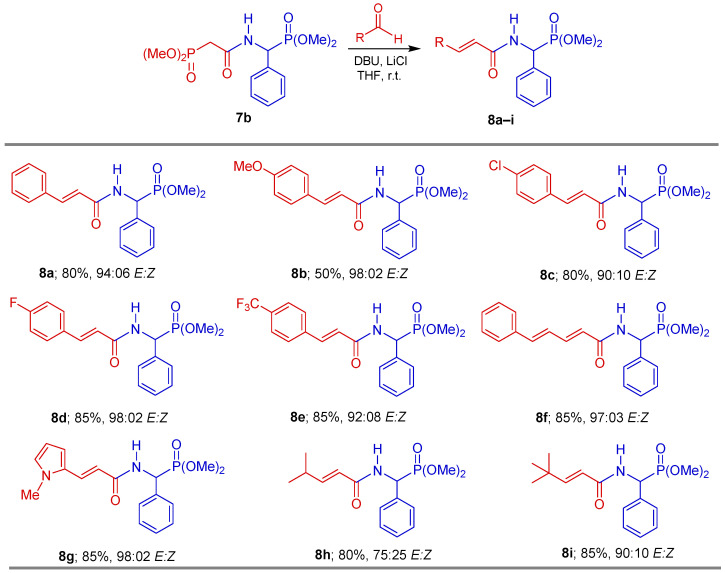
HWE reaction for the synthesis of α,β-unsaturated amides **8a**–**i**.

Additionally, the HWE reaction of the phosphonoacetamide **7b** with benzaldehyde, *4*-methoxybenzaldehyde, *4*-chlorobenzaldehyde, *4*-fluorobenzaldehyde, *4*-trifluoromethylbenzaldehyde, *trans*-cinnamaldehyde and *N*-methyl-2-pyrrolecarboxaldehyde in the presence of DBU and LiCl in THF at 25 °C, produced the α,β-unsaturated amides **8a**–**g** in 50 to 85% yield and *E:Z* selectivities ranging from 90:10 to 98:02. In a similar way, the HWE reaction of the phosphonoacetamide **7b** with isobutyraldehyde and trimethylacetaldehyde, gave the α,β-unsaturated amides **8h**,**i** in good yield and *E:Z* selectivities of 75:25 and 98:02, respectively. The *E:Z* ratio was determined by ^1^H NMR in the crude product and isolated yields after purification by column chromatography (Scheme 6).

**Scheme 7:**
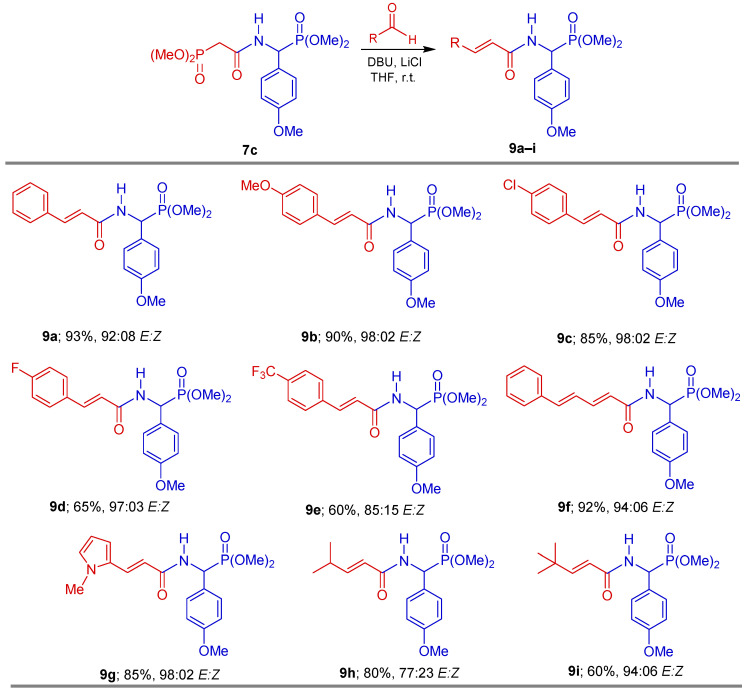
HWE reaction for the synthesis of α,β-unsaturated amides **9a**–**i**.

Finally, the HWE reaction of phosphonoacetamide **7c** with various aldehydes including benzaldehyde, *4*-methoxybenzaldehyde, *4*-chlorobenzaldehyde, *4*-fluorobenzaldehyde, *4*-trifluoromethylbenzaldehyde, isobutyraldehyde and trimethylacetaldehyde in the presence of DBU and LiCl in THF at 25 °C, afforded the α,β-unsaturated amides **9a**–**i** in 60% to 93% and *E:Z* selectivities from 85:15 to 98:02 The *E:Z* ratio was determined by ^1^H NMR in the crude product and isolated yields after purification by column chromatography (Scheme 7).

## 3. Materials and Methods

All commercial materials were used as received unless otherwise noted. Flash chromatography was performed with 230–400 mesh Silica Flash 60^®^. Thin layer chromatography was performed with pre-coated TLC sheets of silica gel (60 F_254_, Merck) and the plates were visualized with UV-light and ninhydrin and phosphomolybdic acid. Melting points were determined with a Fisher-Johns apparatus and are uncorrected. ^1^H, ^13^C, ^31^P NMR spectra were recorded in CDCl_3_ or CD_3_OD on a Bruker AVANCE III HD (500.13 MHz) and JEOL (600 MHz) using tetramethylsilane (TMS) as internal reference and (85% H_3_PO_4_) for ^31^P NMR. Chemical shifts (δ) are expressed in parts per million (ppm) and coupling constants (*J*) in Hertz. High resolution FAB(+) and CI(+) mass spectra (HRMS) were obtained on a JEOL MStation MS-700.

**General procedure for the synthesis of *N*-benzyl α-aminophosphonates (4a–c).**

To a mixture of the corresponding aldehyde; isobutyraldehyde, benzaldehyde and 4-methoxybenzaldehyde (1.0 equiv) and benzylamine (1.0 equiv) was added phenylboronic acid (10 mol%). The reaction mixture was stirred at room temperature for 15 min. After this time, dimethyl phosphite (1.1 equiv) was added and the reaction mixture was stirred at 50 °C. The crude was purified by flash chromatography on silica gel using ethyl acetate-hexane (70:30), obtaining the pure *N-*benzyl α-aminophosphonates **4a**–**c**. The spectroscopic data for **4a** [31], **4b,c** [35]**,** are identical to those described in the literature.

**General procedure for the synthesis of α-aminophosphonates (5a–c).**

A mixture of *N*-benzyl α-aminophosphonates (1.0 equiv) and Pd/C (10%) in 20 mL of methanol was stirred under hydrogen pressure using a balloon for 2 h at 25 °C. The crude was filtered using celite and 20 mL of ethyl acetate. The solvent was evaporated under vacuum, obtaining the α-aminophosphonates **5a**–**c**. The spectroscopic data for **5a,b** [36,37], are identical to those described in the literature. Compound **5c** was used without further purification

**General procedure for the synthesis of bromoamides (6a–c).**

To a mixture of α-aminophosphonates **5a**–**c** (1.0 equiv) and potassium carbonate (2.3 equiv) in dichloromethane:water (40:10) (50 mL) was cooled at 0 °C and stirred for 15 min, followed by the dropwise addition of bromoacetyl bromide (1.1 equiv), and the reaction mixture was stirred at 25 °C for 2 h. After this time, water (20 mL) was added and extracted with dichloromethane (3 × 20 mL). The combined organic extracts were dried over Na_2_SO_4_, filtered and evaporated in vacuum. The crude products were purified by flash column chromatography on silica gel using ethyl acetate-hexane (80:20) as eluent, obtaining the corresponding bromoamides **6a**–**c**.

Dimethyl (1-(2-bromoacetamido)-2-methylpropyl)phosphonate (**6a**). α-Aminophosphonate **5a** (1.10 g, 6 mmol), potassium carbonate (1.98 g, 14 mmol) and bromoacetyl bromide (1.4 g, 0.581 mL, 6.9 mmol) were reacted, obtaining the product **6a** (77% yield) as a white solid, Mp: 108–110 °C. ^1^H NMR (CDCl_3_, 500 MHz): *δ* 1.03 (d, *J* = 6.8 Hz, 3H, (CH_3_)_2_CH), 1.05 (d, *J* = 6.9 Hz, 3H, (CH_3_)_2_CH), 2.26 (oct, *J =* 6.9 Hz, 1H, CH(CH_3_)_2_), 3.77 (d, *J* = 10.7 Hz, 3H, (CH_3_O)_2_P), 3.78 (d, *J* = 10.7 Hz, 3H, (CH_3_O)_2_P), 3.92 (AB system, *J* = 13.2 Hz, 1H, CH_2_Br), 3.96 (AB system, *J* = 13.4 Hz, 1H, CH_2_Br), 4.36 (ddd, *J* = 17.9, 10.4, 4.5 Hz, 1H, CH-P), 6.83 (d, *J* = 10.4 Hz, 1H, NH). ^13^C NMR (CDCl_3_, 125 MHz): *δ* 17.9 (d, *J* = 4.6 Hz, (CH_3_)_2_CH), 20.4 (d, *J* = 12.4 Hz, (CH_3_)_2_CH), 28.8 (CH_2_Br), 28.9 (d, *J* = 3.6 Hz, CH(CH_3_)_2_), 50.5 (d, *J* = 152.9 Hz, C-P), 53.0 (d, *J* = 6.8 Hz, (CH_3_O)_2_P), 53.2 (d, *J* = 6.8 Hz, (CH_3_O)_2_P), 165.7 (d, *J* = 5.6 Hz, C=O). ^31^P NMR (CDCl_3_, 202 MHz): *δ* 25.9 ppm. HRMS-CI(+) *m/z*: [M + H]^+^ Calcd for C_8_H_18_BrNO_4_P + H^+^ 302.0151; Found 302.0161.

Dimethyl ((2-bromoacetamido)(phenyl)methyl)phosphonate (**6b**). α-Aminophosphonate **5b** (0.46 g, 2.1 mmol), potassium carbonate (0.70 g, 5 mmol) and of bromoacetyl bromide (0.47 g, 0.20 mL, 2.3 mmol) were reacted, obtaining the product **6b** (72% yield) as a white solid, Mp: 143–145 °C. ^1^H NMR (CDCl_3_, 500 MHz): *δ* 3.50 (d, *J* = 10.7 Hz, 3H, (CH_3_O)_2_P), 3.84 (d, *J* = 10.8 Hz, 3H, (CH_3_O)_2_P), 3.87 (s, 2H, CH_2_Br), 5.55 (dd, *J* = 20.7, 9.6 Hz, 1H, CH-P), 7.31–7.40 (m, 3H, H_arom_), 7.48–7.52 (m, 2H, H_arom_), 8.33 (d, *J* = 9.6 Hz, 1H, NH). ^13^C NMR (CDCl_3_, 125 MHz): *δ* 28.6 (CH_2_Br), 50.5 (d, *J* = 155.3 Hz, C-P), 54.1 (d, *J* = 6.8 Hz, (CH_3_O)_2_P), 54.3 (d, *J* = 6.8 Hz, (CH_3_O)_2_P), 128.3, 128.4, 128.7, 129.1 (2C), 134.3, 166.0 (d, *J* = 7.7 Hz, C=O). ^31^P NMR (CDCl_3_, 202 MHz): *δ* 23.0 ppm. HRMS-CI(+) *m/z*: [M + H]^+^ Calcd for C_11_H_16_BrNO_4_P + H^+^ 335.9995; Found 335.9998.

Dimethyl ((2-bromoacetamido)(4-methoxyphenyl)methyl)phosphonate (**6c**). α-Aminophosphonate **5c** (0.60 g, 2.4 mmol), potassium carbonate (0.80 g, 5.7 mmol) and bromoacetyl bromide (0.54 g, 0.23 mL, 2.6 mmol) were reacted, obtaining the product **6c** (80% yield) as a white solid, Mp: 134–135 °C. ^1^H NMR (CDCl_3_, 500 MHz): *δ* 3.51 (d, *J* = 10.7 Hz, 3H, (CH_3_O)_2_P), 3.80 (s, 3H, CH_3_O), 3.83 (d, *J* = 10.7 Hz, 3H, (CH_3_O)_2_P), 3.87 (s, 2H, CH_2_Br), 5.49 (dd, *J* = 20.3, 9.6 Hz, 1H, CH-P), 6.89 (AA’BB’ system, *J* = 8.7 Hz, 2H, H_arom_), 7.42 (AA’BB’ system, *J* = 8.7, Hz, 2H, H_arom_), 8.24 (d, *J* = 9.6 Hz, 1H, NH). ^13^C NMR (CDCl_3_, 125 MHz): *δ* 28.6 (CH_2_Br), 49.8 (d, *J* = 156.7 Hz, C-P), 53.9 (d, *J* = 6.8 Hz, (CH_3_O)_2_P), 54.2 (d, *J* = 6.8 Hz, (CH_3_O)_2_P), 55.5 (CH_3_O), 114.5 (2C), 126.3, 129.6, 129.7, 159.9, 165.9 (d, *J* = 8.2 Hz, C=O). ^31^P NMR (CDCl_3_, 202 MHz): *δ* 23.3 ppm. HRMS-CI(+) *m/z*: [M + H]^+^ Calcd for C_12_H_18_BrNO_5_P + H^+^ 366.0100; Found 366.0110.

**General procedure for the synthesis of phosphonoacetamides (7a–c).**

A mixture of bromoamides **6a**–**c** (1.0 equiv) and trimethyl phosphite (4.0 equiv) without solvent was heated for 4 h at 105–110 °C. After this time, the reaction mixture was purified by flash column chromatography using ethyl acetate-methanol (90:10) as the eluent, obtaining the phosphonoacetamides **7a**–**c**.

Dimethyl (2-((1-(dimethoxyphosphoryl)-2-methylpropyl)amino)-2-oxoethyl)phosphonate (**7a**). Bromoamide **6a** (0.50 g, 1.6 mmol) and trimethyl phosphite (0.62 g, 0.52 mL, 4.9 mmol) were reacted, obtaining the product **7a** (98% yield) as a white solid, Mp: 80–83 °C. ^1^H NMR (CDCl_3_, 500 MHz): *δ* 1.02 (dd, *J* = 6.8, 1.4 Hz, 3H, (CH_3_)_2_CH), 1.05 (d, *J* = 6.9 Hz, 3H, (CH_3_)_2_CH), 2.21–2.26 (m, 1H, CH(CH_3_)_2_), 2.99 (dd, *J* = 24.3, 14.7 Hz, 1H, CH_2_P), 3.03 (dd, *J* = 24.3, 14.8 Hz, 1H, CH_2_P), 3.76 (d, *J* = 10.7 Hz, 3H, (CH_3_O)_2_P), 3.78 (d, *J* = 10.7 Hz, 3H, (CH_3_O) _2_P), 3.80 (d, *J* = 11.1 Hz, 3H, (CH_3_O)_2_P), 3.82 (d, *J* = 11.1 Hz, 3H, (CH_3_O)_2_P), 4.42 (ddd, *J* = 17.9, 10.4, 4.4 Hz, 1H, CH-P), 7.27 (d, *J* = 10.4 Hz, 1H, NH). ^13^C NMR (CDCl_3_, 125 MHz): *δ* 17.8 (d, *J* = 4.5 Hz, (CH_3_)_2_CH), 20.4 (d, *J* = 13.2 Hz, (CH_3_)_2_CH), 28.9 (d, *J* = 3.6 Hz, CH(CH_3_)_2_), 34.1 (d, *J* = 152.4 Hz, C-P), 50.0 (d, *J* = 153.0 Hz, C-P), 52.9 (d, *J* = 6.8 Hz, (CH_3_O)_2_P), 52.9 (d *J* = 6.8 Hz (CH_3_O)_2_P), 53.0 (d, *J* = 6.8 Hz, (CH_3_O)_2_P), 53.8 (d, *J* = 6.8 Hz, (CH_3_O)_2_P), 164.1 (d, *J* = 5.6 Hz, C=O). ^31^P NMR (CDCl_3_, 202 MHz): *δ* 24.9, 26.3 ppm. HRMS-CI(+) *m/z*: [M + H]^+^ Calcd for C_10_H_24_NO_7_P_2_ + H^+^ 332.1023; Found 332.1021.

Dimethyl (2-(((dimethoxyphosphoryl)(phenyl)methyl)amino)-2-oxoethyl)phosphonate (**7b**). Bromoamide **6b** (0.62 g, 1.8 mmol) and trimethyl phosphite (0.68 g, 0.57 mL, 5.4 mmol) were reacted, obtaining the product **7b** (95% yield) as a white solid, Mp: 101–104 °C. ^1^H NMR (CDCl_3_, 600 MHz): *δ* 2.99 (dd, *J* = 21.7, 14.9 Hz, 1H, CH_2_P), 3.02 (dd, *J* = 21.7, 14.8 Hz, 1H, CH_2_P), 3.54 (d, *J* = 11.1 Hz, 3H, (CH_3_O)_2_P), 3.67 (d, *J* = 11.3 Hz, 3H, (CH_3_O)_2_P), 3.77 (d, *J* = 11.2 Hz, 3H, (CH_3_O)_2_P), 3.80 (d, *J* = 10.7 Hz, 3H, (CH_3_O)_2_P), 5.59 (dd, *J* = 21.7, 9.6 Hz, 1H, CH-P), 7.31–7.36 (m, 3H, H_arom_), 7.49–730 (m, 2H, H_arom_), 8.40 (d, *J* = 9.6 Hz, 1H, NH). ^13^C NMR (CDCl_3_, 151 MHz): *δ* 34.3 (d, *J* = 132.6 Hz, C-P), 49.9 (d, *J* = 155.0 Hz, C-P), 53.1 (d, *J* = 6.3 Hz, (CH_3_O)_2_P), 53.2 (d, *J* = 6.5 Hz, (CH_3_O)_2_P, 53.8 (d, *J* = 7.0 Hz, (CH_3_O)_2_P, 53.9 (d, *J* = 6.9 Hz, (CH_3_O)_2_P), 128.2 (2C),128.4, 128.7 (2C), 134.5, 163.9 (dd, *J* = 8.3, 5.3 Hz, C=O). ^31^P NMR (CDCl_3_, 202 MHz): *δ* 23.3, 24.6 ppm. HRMS-CI(+) *m/z*: [M + H]^+^ Calcd for C_13_H_22_NO_7_P_2_ + H^+^ 366.0866; Found 366.0871.

Dimethyl (2-(((dimethoxyphosphoryl)(4-methoxyphenyl)methyl)amino)-2-oxoethyl)phosphonate (**7c**). Bromoamide **6c** (0.32 g, 0.8 mmol) and trimethyl phosphite (0.33 g, 0.27 mL, 2.6 mmol) were reacted, obtaining the product **7c** (96% yield) as a white solid, Mp: 133–135 °C. ^1^H NMR (CDCl_3_, 600 MHz): *δ* 2.96 (dd, *J* = 24.2, 14.9 Hz, 1H, CH_2_P), 3.01 (dd, *J* = 24.2, 14.9 Hz, 1H, CH_2_P), 3.55 (d, *J* = 10.6 Hz, 3H, (CH_3_O)_2_P), 3.68 (d, *J* = 11.2 Hz, 3H, (CH_3_O)_2_P), 3.78 (d, *J* = 10.5 Hz, 3H, (CH_3_O)_2_P), 3.79 (d, *J* = 11.1 Hz, 3H, (CH_3_O)_2_P), 3.79 (s, 3H, CH_3_O), 5.53 (dd, *J* = 20.4, 9.6 Hz, 1H, CH-P), 6.88 (AA’BB’ system, *J* = 8.6 Hz, 2H, H_arom_), 7.42 (AA’BB’ system, *J* = 8.6 Hz, 2H, H_arom_), 8.30 (d, *J* = 9.6 Hz, 1H, NH). ^13^C NMR (CDCl_3_, 151 MHz): *δ* 34.3 (d, *J* = 132.2 Hz, CH_2_-P), 49.3 (d, *J* = 156.1 Hz, C-P), 53.2 (d, *J* = 6.3 Hz, (CH_3_O)_2_P), 53.3 (d, *J* = 7.0 Hz, (CH_3_O)_2_P), 53.8 (d, *J* = 6.3 Hz, (CH_3_O)_2_P), 53.9 (d, *J* = 7.0 Hz, (CH_3_O)_2_P), 55.3 (CH_3_O), 114.2 (2C), 126.4, 129.4 (2C), 159.6, 163.7 (d, *J* = 5.1 Hz, C=O). ^31^P NMR (CDCl_3_, 202 MHz): *δ* 23.5, 24.6 ppm.

**General procedure for the synthesis of α,β-unsaturated amides 3a–f.**

A solution of *trans*-cinnamic acid (1.1 equiv), PyBOP (1.3 equiv) in anhydrous MeCN (2 mL) and DIPEA (3.0 equiv) was treated at 0 °C under nitrogen atmosphere with dimethyl (1-amino-2-methylpropyl)phosphonate **5a** (1.0 equiv) in MeCN (1 mL) and the reaction mixture was stirred at room temperature for 24 h. After this time, the solvent was evaporated in vacuum, and the resulting residue was purified by column chromatography using ethyl acetate:hexane (80:20) as the eluent, to give the α,β-unsaturated amides **3a**–**f**.

Dimethyl *N*-[3-phenyl-2-ene-1-oxo]-2-(methylpropyl)phosphonate (**3a**). *trans*-Cinnamic acid (120 mg, 0.81 mmol), PyBOP (500 mg, 0.96mmol), anhydrous MeCN (2 mL), DIPEA (300 mg, 0.4 mL, 2.21mmol) and dimethyl (1-amino-2-methylpropyl)phosphonate **5a** (134 mg, 0.73 mmol) were reacted, obtaining the product **3a** (77% yield, only the diastereoisomer *E*) as white solid, Mp: 133 °C. ^1^H NMR (CD_3_OD, 500 MHz): *δ* 1.05 (dd, *J* = 6.8, 1.4 Hz, 3H, (CH_3_)_2_CH), 1.06 (d, *J* = 6.9 Hz, 3H, (CH_3_)_2_CH), 2.22 (oct, *J* = 6.9 Hz, 1H, CH(CH_3_)_2_), 3.77 (d, *J* = 10.7 Hz, 3H, (CH_3_O)_2_P), 3.79 (d, *J* = 10.7 Hz, 3H, (CH_3_O)_2_P), 4.48 (dd, *J* = 17.9, 5.8 Hz, 1H, CH-P), 6.77 (d, *J_trans_* = 15.9 Hz, 1H, CH=CH), 7.35–7.43 (m, 3H, H_arom_), 7.59–7.55 (m, 2H, H_arom_), 7.60 (d, *J_trans_* = 15.6 Hz, 1H, CH=CH). ^13^C NMR (CD_3_OD, 126 MHz): *δ* 17.7 (d, *J* = 4.5 Hz, (CH_3_)_2_CH), 19.6 (d, *J* = 12.5 Hz, (CH_3_)_2_CH), 29.1 (d, *J* = 3.2 Hz, CH(CH_3_)_2_), 50.4 (d, *J* = 152.4 Hz, C-P), 52.5 (d, *J* = 6.8 Hz, (CH_3_O)_2_P), 52.6 (d, *J* = 6.4 Hz, (CH_3_O)_2_P), 119.8 (C=C), 127.7, 128.8, 129.8, 135.0, 141.7 (C=C), 167.4 (d, *J* = 5.9 Hz, C=O). ^31^P NMR (CD_3_OD, 202 MHz): δ 27.0 ppm. HRMS-FAB(+) *m/z*: [M + H]^+^ Calcd for C_15_H_23_NO_4_P + H^+^ 312.1365; Found 312.1332.

Dimethyl *N*-[3-(4-methoxyphenyl)-2-ene-1-oxo]-2-(methylpropyl)phosphonate (**3b**). (*E*)-4-Methoxycinnamic acid (140 mg, 0.81 mmol), PyBOP (500 mg, 0.96 mmol), anhydrous MeCN (2 mL), DIPEA (300 mg, 0.4 mL, 2.21 mmol) and dimethyl (1-amino-2-methylpropyl)phosphonate **5a** (134 mg, 0.73 mmol) were reacted, obtaining the product **3b** (74% yield, only the diastereoisomer *E*) as a white solid, Mp: 136–137 °C. ^1^H NMR (CDCl_3_, 600 MHz): *δ* 1.04 (dd, *J =* 6.8, 1.3 Hz, 3H, (CH_3_)_2_CH), 1.07 (d, *J =* 6.8 Hz, 3H, (CH_3_)_2_CH), 2.30–2.20 (m, 1H, CH(CH_3_)_2_), 3.77 (d, *J =* 10.6 Hz, 3H, (CH_3_O)_2_P), 3.79 (d, *J =* 10.7 Hz, 3H, (CH_3_O)_2_P), 3.83 (s, 3H, CH_3_O), 4.62 (ddd, *J =* 17.9, 10.4, 4.7 Hz, 1H, CH-P), 6.52 (d, *J_trans_ =* 15.6 Hz, 1H, CH=CH), 6.80 (d, *J =* 10.4 Hz, 1H, NH), 6.88 (AA’BB’ system, *J =* 8.8 Hz, 2H, H_arom_), 7.47 (AA’BB’ system, *J =* 8.8 Hz, 2H, H_arom_), 7.63 (d, *J_trans_ =* 15.6 Hz, 1H, CH=CH). ^13^C NMR (CDCl_3_, 151 MHz): *δ* 18.2 (d, *J =* 4.5 Hz, (CH_3_)_2_CH), 20.4 (d, *J =* 12.3 Hz, (CH_3_)_2_CH), 29.2 (d, *J =* 4.0 Hz, CH(CH_3_)_2_), 49.7 (d, *J =* 152.4 Hz, C-P), 52.7 (d, *J =* 6.8 Hz, (CH_3_O)_2_P), 53.4 (d, *J =* 6.8 Hz, (CH_3_O)_2_P), 55.4 (CH_3_O), 114.3, 117.8 (C=C), 127.6, 129.5, 141.5 (C=C), 161.0, 166.5 (d, *J =* 5.8 Hz, C=O). ^31^P NMR (CDCl_3_, 243 MHz): δ 27.5 ppm. HRMS-FAB(+) *m/z*: [M + H]^+^ Calcd for C_16_H_25_NO_5_P + H^+^ 342.1465; Found 342.1489.

Dimethyl *N*-[3-(3,4-dimethoxyphenyl)-2-ene-1-oxo]-2-(methylpropyl)phosphonate (**3c**). (*E*)-3,4-Dimethoxycinnamic acid (140 mg, 0.81 mmol), PyBOP (500 mg, 0.96 mmol), anhydrous MeCN (2 mL), DIPEA (300 mg, 0.4 mL, 2.21 mmol) and dimethyl (1-amino-2-methylpropyl)phosphonate **5a** (134 mg, 0.73 mmol) were reacted, obtaining the product **3c** (68% yield, only the diastereoisomer *E*) as a colorless liquid. ^1^H NMR (CDCl_3_, 500 MHz): *δ* 1.05 (dd, *J* = 6.9, 1.3 Hz, 3H, (CH_3_)_2_CH), 1.08 (d, *J* = 6.9 Hz, 3H, (CH_3_)_2_CH), 2.26 (oct, *J* = 6.9 Hz, 1H, CH(CH_3_)_2_, 3.75 (d*, J* = 10.6 Hz, 3H, (CH_3_O)_2_P), 3.81 (d*, J* = 10.7 Hz, 3H, (CH_3_O)_2_P), 3.83 (s, 3H, CH_3_O), 3.92 (s, 3H, CH_3_O), 4.63 (ddd, *J* = 18.0, 10.5, 4.6 Hz, CH-P), 6.48 (d, *J_trans_* = 15.5 Hz, 1H, CH=CH), 6.60 (d, *J* = 10.5 Hz, 1H, NH), 6.86 (d, *J* = 8.3 Hz, 1H, H_arom_), 7.06 (d, *J* = 2.0 Hz, 1H, H_arom_), 7.10 (dd, *J* = 8.3, 2.0 Hz, 1H, H_arom_), 7.62 (d, *J_trans_* = 15.5 Hz, 1H, CH=CH). ^13^C NMR (CDCl_3_, 126 MHz): *δ* 18.1 (d, *J =* 4.8 Hz, (CH_3_)_2_CH), 20.4 (d, *J =* 12.4 Hz, (CH_3_)_2_CH), 29.1 (d, *J =* 3.8 Hz, CH(CH_3_)_2_), 49.7 (d, *J* = 152.4 Hz, C-P), 52.8 (d, *J* = 6.9 Hz, (CH_3_O)_2_P), 53.3 (d, *J* = 6.9 Hz, (CH_3_O)_2_P), 55.8 (CH_3_O), 55.9 (CH_3_O), 109.5 (C=C), 111.0 (C=C), 117.8, 122.2, 127.7, 141.8, 149.1, 150.7, 166.3 (d, *J* = 5.7 Hz, C=O). ^31^P NMR (CDCl_3_, 202 MHz): *δ* 26.8 ppm. HRMS-FAB(+) *m/z*: Calcd for C_17_H_27_NO_6_P + H^+^ 372.1571; Found 372.1609.

Dimethyl *N*-[3-(4-chlorophenyl)-2-ene-1-oxo]-2-(methylpropyl)phosphonate (**3d**). (*E*)-4-Chlorocinnamic acid (150 mg, 0.81 mmol), PyBOP (500 mg, 0.96 mmol), anhydrous MeCN (2 mL), DIPEA (300 mg, 0.4 mL, 2.21 mmol) and dimethyl (1-amino-2-methylpropyl)phosphonate **5a** (134 mg, 0.73 mmol) were reacted, obtaining the product **3d** (72% yield, only the diastereoisomer *E*) as a white solid, Mp: 109–110 °C. ^1^H NMR (CDCl_3_, 500 MHz): *δ* 1.05 (dd, *J* = 6.9, 1.2 Hz, 3H, (CH_3_)_2_CH), 1.09 (d, *J* = 7.0 Hz, 3H, (CH_3_)_2_CH), 2.25 (oct, *J* = 6.8 Hz, 1H, CH(CH_3_)_2_), 3.74 (d, *J* = 10.7 Hz, 3H, (CH_3_O)_2_P), 3.84 (d, *J* = 10.7 Hz, 3H, (CH_3_O)_2_P), 4.64 (ddd, *J* = 17.7, 10.2, 5.0 Hz, 1H, CH-P), 6.75 (d, *J_trans_* = 15.7 Hz, 1H, CH=CH), 7.32 (AA’BB’ system, *J* = 8.5 Hz, 2H, H_arom_), 7.44 (AA’BB’ system, *J* = 8.5 Hz, 2H, H_arom_), 7.59 (d, *J* = 10.2 Hz, 1H, NH), 7.64 (d, *J_trans_* = 15.7 Hz, 1H, CH=CH). ^13^C NMR (CDCl_3_, 151 MHz): *δ* 18.2 (d, *J* = 5.4 Hz, (CH_3_)_2_CH), 20.4 (d, *J* = 11.9 Hz, (CH_3_)_2_CH), 29.2 (d, *J* = 3.6 Hz, CH(CH_3_)_2_), 49.8 (d, *J =* 152.1 Hz, C-P), 52.8 (d, *J* = 6.9 Hz, (CH_3_O)_2_P), 53.5 (d, *J* = 6.9 Hz, (CH_3_O)_2_P), 120.9 (C=C), 129.1 (2C), 133.5 (2C), 140.5 (C=C), 166.1 (d, *J* = 5.8 Hz, C=O). ^31^P NMR (CDCl_3_, 202 MHz): *δ* 26.4 ppm. HRMS-FAB(+) *m/z*: [M + H]^+^ Calcd for C_15_H_22_ClNO_4_P + H^+^ 346.0969; Found 346.0973.

Dimethyl *N*-[3-(4-fluorophenyl)-2-ene-1-oxo]-2-(methylpropyl)phosphonate (**3e**). (*E*)-4-Fluorocinnamic acid (160 mg, 0.81 mmol), PyBOP (500 mg, 0.96 mmol), anhydrous MeCN (2 mL), DIPEA (300 mg, 0.4 mL, 2.21 mmol) and dimethyl (1-amino-2-methylpropyl)phosphonate **5a** (134 mg, 0.73 mmol) were reacted, obtaining the product **3e** (70% yield, only the diastereoisomer *E*) as a white solid, Mp: 138–140 °C. ^1^H NMR (CDCl_3_, 500 MHz): *δ* 1.05 (dd, *J* = 6.9, 1.2 Hz, 3H, (CH_3_)_2_CH), 1.09 (d, *J* = 7.0 Hz, 3H, (CH_3_)_2_CH), 2.25 (oct, *J* = 6.7 Hz, 1H, CH(CH_3_)_2_), 3.74 (d, *J* = 10.7 Hz, 3H, (CH_3_O)_2_P), 3.84 (d, *J* = 10.7 Hz, 3H, (CH_3_O)_2_P), 4.64 (ddd, *J* = 17.7, 10.2, 5.0 Hz, 1H, CH-P), 6.75 (d, *J_trans_* = 15.7 Hz, 1H, CH=CH), 7.04 (dd, *J* = 8.7, 5.7 Hz, 2H, H_arom_), 7.50 (dd, *J* = 8.8, 5.3 Hz, 2H, H_arom_), 7.59 (d, *J* = 10.2 Hz, 1H, NH), 7.64 (d, *J_trans_* = 15.7 Hz, 1H, CH=CH). ^13^C NMR (CDCl_3_, 151 MHz): *δ* 18.1 (d, *J* = 4.7 Hz, (CH_3_)_2_CH), 20.5 (d, *J* = 12.6 Hz, (CH_3_)_2_CH), 29.2 (d, *J* = 3.6 Hz, CH(CH_3_)_2_), 49.7 (d, *J =* 152.4 Hz, C-P), 52.9 (d, *J* = 6.9 Hz, (CH_3_O)_2_P), 53.3 (d, *J* = 7.2 Hz, (CH_3_O)_2_P), 116.0 (2C, *J* = 22.0 Hz), 119.8 (C=C), 129.8 (2C, *J* = 8.3 Hz), 140.9 (C=C), 163.7 (d, *J* = 250.7 Hz, C-F), 165.8 (d, *J* = 5.8 Hz, C=O). ^31^P NMR (CDCl_3_, 202 MHz): *δ* 26.5 ppm. HRMS-FAB(+) *m/z*: [M + H]^+^ Calcd for C_15_H_22_FNO_4_P + H^+^ 330.1265; Found 330.1250.

Dimethyl *N*-[3-(4-(trifluoromethyl)phenyl)-2-ene-1-oxo]-2-(methylpropyl)phosphonate (**3f**). (*E*)-4-(Trifluoromethyl)cinnamic acid (160 mg, 0.81 mmol), PyBOP (500 mg, 0.96 mmol), anhydrous MeCN (2 mL), DIPEA (300 mg, 0.4 mL, 2.21 mmol) and dimethyl (1-amino-2-methylpropyl)phosphonate **5a** (134 mg, 0.73 mmol) were reacted, obtaining the product **3f** (71% yield, only the diastereoisomer *E*) as a white solid, Mp: 139–140 °C. ^1^H RMN (CDCl_3_, 600 MHz): *δ* 1.05 (dd, *J* = 6.8, 1.3 Hz, 3H, (CH_3_)_2_CH), 1.08 (d, *J* = 6.9 Hz, 3H, (CH_3_)_2_CH), 2.27 (oct, *J* = 6.8 Hz, 1H, CH(CH_3_)_2_), 3.75 (d, *J* = 10.7 Hz, 3H, (CH_3_O)_2_P), 3.83 (d, *J* = 10.7 Hz, 3H, (CH_3_O)_2_P), 4.62 (ddd, *J* = 18.0, 10.1, 4.7 Hz, 1H, CH-P), 6.73 (d, *J_trans_* = 15.7 Hz, 1H, CH=CH), 6.88 (d, *J* = 10.1 Hz, 1H, NH), 7.62 (s, 4H, H_arom_), 7.69 (d, *J_trans_* = 15.7 Hz, 1H, CH=CH). ^13^C NMR (CDCl_3_, 125 MHz): *δ* 18.3 (d, *J* = 5.0 Hz, (CH_3_)_2_CH), 20.6 (d, *J* = 12.3 Hz, (CH_3_)_2_CH), 29.3 (d, *J* = 3.6 Hz, CH(CH_3_)_2_), 49.9 (d, *J* = 152.6 Hz, C-P), 53.0 (d, *J* = 6.8 Hz, (CH_3_O)_2_P), 53.6 (d, *J* = 6.8 Hz, (CH_3_O)_2_P), 122.9 (C=C), 124.1 (q, *J* = 272.0 Hz, CF_3_), 126.0 (2C, *J* = 4.1 Hz), 128.2, 131.5 (q, *J* = 32.2 Hz, C-CF_3_), 138.5, 140.3 (C=C), 165.7 (d, *J* = 5.4 Hz, C=O). ^31^P NMR (CDCl_3_, 243 MHz): *δ* 26.5 ppm. HRMS-FAB(+) *m/z*: [M + H]^+^ Calcd for C_16_H_22_F_3_NO_4_P + H^+^ 380.1233; Found 380.1250.

**General procedure for the synthesis of α,β-unsaturated amides 3, 8 and 9.**

A solution of phosphonoacetamides **7a**–**c** (1.0 equiv), lithium chloride (3.0 equiv) in dry THF (10 mL) was treated with 1,8-diazabicyclo[5,4,0]undec-7-ene (DBU) (3.0 equiv) under a nitrogen atmosphere at 25 °C. After stirring for 5 min, the corresponding aldehyde (1.0 equiv) was added and the reaction mixture was stirred at 25 °C for 4 h (the progress of the reaction was monitored by thin-layer chromatography). When the reaction was complete, a saturated ammonium chloride solution (10 mL) was added, and extracted with ethyl acetate (3 × 10 mL). The combined organic extracts were dried over Na_2_SO_4_, filtered and evaporated in vacuum. The crude products were purified by flash column chromatography using ethyl acetate as the eluent, obtaining the corresponding (*E*)-α,β-unsaturated amides incorporating α-aminophosphonates. Compounds **9f** and **9g** were unstable and clean NMR spectra could not be obtained.

Dimethyl *N*-[3-phenyl-2-ene-1-oxo]-2-(methylpropyl)phosphonate (**3a**). Phosphonoacetamide **7a** (0.20 g, 0.6 mmol), lithium chloride (70 mg, 1.6 mmol), DBU (0.27 g, 0.27 mL, 1.7 mmol) and benzaldehyde (60 mg, 0.06 mL, 0.6 mmol) were reacted, obtaining the product **3a** (90% yield, only the diastereoisomer *E*) as a white solid, Mp: 133 °C. The spectroscopic data are identical to those described above.

Dimethyl *N*-[3-(4-methoxyphenyl)-2-ene-1-oxo]-2-(methylpropyl)phosphonate (**3b**). Phosphonoacetamide **7a** (0.20 g, 0.6 mmol), lithium chloride (70 mg, 1.6 mmol), DBU (0.27 g, 0.27 mL, 1.7 mmol) and *4-*methoxybenzaldehyde (80 mg, 0.07 mL, 0.6 mmol) were reacted, obtaining the product **3b** (80% yield, only the diastereoisomer *E*) as a white solid, Mp: 136–137 °C. The spectroscopic data are identical to those described above.

Dimethyl *N*-[3-(4-chlorophenyl)-2-ene-1-oxo]-2-(methylpropyl)phosphonate (**3d**). Phosphonoacetamide **7a** (0.20 g, 0.6 mmol), lithium chloride (70 mg, 1.6 mmol), DBU (0.27 g, 0.27 mL, 1.7 mmol) and *4-*chlorobenzaldehyde (80 mg, 0.6 mmol) were reacted, obtaining the product **3d** (85% yield, only the diastereoisomer *E*) as a white solid, Mp: 109–110 °C. The spectroscopic data are identical to those described above.

Dimethyl *N*-[3-(4-fluorophenyl)-2-ene-1-oxo]-2-(methylpropyl)phosphonate (**3e**). phosphonoacetamide **7a** (0.20 g, 0.6 mmol), lithium chloride (70 mg, 1.6 mmol), DBU (0.27 g, 0.27 mL, 1.7 mmol) and *4-*fluorobenzaldehyde (70 mg, 0.06 mL, 0.6 mmol) were reacted, obtaining the product **3e** (84% yield, only the diastereoisomer *E*) as a white solid, Mp: 138–140 °C. The spectroscopic data are identical to those described above.

Dimethyl *N*-[3-(4-(trifluoromethyl)phenyl)-2-ene-1-oxo]-2-(methylpropyl)phosphonate (**3f**). phosphonoacetamide **7a** (0.20 g, 0.6 mmol), lithium chloride (70 mg, 1.6 mmol), DBU (0.27 g, 0.27 mL, 1.7 mmol) and *4-*trifluoromethylbenzaldehyde (0.10 g, 0.08 mL, 0.6 mmol) were reacted, obtaining the product **3f** (88% yield, only the diastereoisomer *E*) as a white solid, Mp: 139–140 °C. The spectroscopic data are identical to those described above.

Dimethyl *N*-[3-((4-benzyloxi)-3-methoxyphenyl)-2-ene-1-oxo]-2-(methylpropyl)phosphonate (**3g**). Phosphonoacetamide **7a** (0.20 g, 0.6 mmol), lithium chloride (70 mg, 1.6 mmol), DBU (0.27 g, 0.27 mL, 1.7 mmol) and 4-benzyloxy-3-methoxybenzaldehyde (0.15 g, 0.6 mmol) were reacted, obtaining the product **3g** (80% yield, only the diastereoisomer *E*) as a white solid, Mp: 135–137 °C. ^1^H NMR (CDCl_3_, 500 MHz): *δ* 1.04 (dd, *J* = 6.7, 1.4 Hz, 3H, (CH_3_)_2_CH), 1.07 (d, *J* = 6.9 Hz, 3H, (CH_3_)_2_CH), 2.25 (oct, *J* = 6.8 Hz, 1H, CH(CH_3_)_2_), 3.74 (d, *J* = 10.7 Hz, 3H, (CH_3_O)_2_P), 3.79 (d, *J* = 10.7 Hz, 3H, (CH_3_O)_2_P), 3.92 (s, 3H, CH_3_O), 4.61 (ddd, *J* = 18.0, 10.4, 4.6 Hz, 1H, CH-P), 5.18 (s, 2H, CH_2_Ph), 6.44 (d, *J_trans_* = 15.6 Hz, 1H, CH=CH), 6.42 (d, *J* = 10.4 Hz, 1H, NH), 6.86 (d, *J* = 8.4 Hz, 1H, H_arom_), 7.02 (d, *J* = 8.3 Hz, 1H, H_arom_), 7.06–7.07 (m, 1H, H_arom_), 7.59 (d, *J_trans_* = 15.5 Hz, 1H, CH=CH). ^13^C NMR (CDCl_3_, 125 MHz): *δ* 18.2 (d, *J* = 5.0 Hz, (CH_3_)_2_CH), 20.6 (d, *J* = 12.7 Hz, (CH_3_)_2_CH), 29.3 (d, *J* = 3.6 Hz, CH(CH_3_)_2_), 49.8 (d, *J* = 152.6 Hz, C-P), 53.0 (d, *J* = 6.8 Hz, (CH_3_O)_2_P), 53.5 (d, *J* = 6.8 Hz, (CH_3_O)_2_P), 56.2 (CH_3_O), 71.1 (CH_2_Ph), 110.5, 113.7, 118.2, 122.2 (C=C), 127.4 (2C), 128.2, 128.3, 128.8 (2C), 126.8, 142.0 (C=C), 149.9, 150.1, 166.4 (d, *J* = 5.9 Hz, C=O). ^31^P NMR (CDCl_3_, 202 MHz): *δ* 26.7 ppm. HRMS-CI(+) *m/z*: [M + H]^+^ Calcd for C_23_H_31_NO_6_P + H^+^ 448.1884; Found 448.1866.

Dimethyl *N*-[(2*E,*4*E*)-5-phenylpenta-2,4-dien-1-oxo]-2-(methylpropyl)phosphonate (**3h**). Phosphonoacetamide **7a** (0.20 g, 0.6 mmol), lithium chloride (70 mg, 1.6 mmol), DBU (0.27 g, 0.27 mL, 1.7 mmol) and *trans*-cinnamaldehyde (80 mg, 0.07 mL, (0.6 mmol) were reacted, obtaining the product **3h** (80% yield, only the diastereoisomer *E*) as a yellow solid, Mp: 146–147 °C. ^1^H NMR (CDCl_3_, 500 MHz): *δ* 1.04 (dd, *J* = 6.8, 1.3 Hz, 3H, (CH_3_)_2_CH), 1.06 (d, *J* = 6.9 Hz, 3H, (CH_3_)_2_CH), 2.24 (oct, *J* = 6.7 Hz, 1H, CH(CH_3_)_2_), 3.75 (d, *J* = 10.5 Hz, 3H, (CH_3_O)_2_P), 3.81 (d, *J* = 10.7 Hz, 3H, (CH_3_O)_2_P), 4.59 (ddd, *J* = 18.0, 10.4, 4.7 Hz, 1H, CH-P), 6.14 (d, *J_trans_* = 15.0 Hz, 1H, CH=CH), 6.46–6.48 (m, 1H, CH=CH), 6.82–6.90 (m, 2H, H_arom_), 7.28–7.31 (m, 1H, H_arom_), 7.33–7.36 (m, 2H, H_arom_), 7.42–7.47 (m, 3H, H_arom_ and CH=CH). ^13^C NMR (CDCl_3_, 125 MHz): *δ* 18.3 (d, *J* = 5.0 Hz, (CH_3_)_2_CH), 20.6 (d, *J* = 12.3 Hz, (CH_3_)_2_CH), 29.3 (d, *J* = 3.6 Hz, CH(CH_3_)_2_), 49.8 (d, *J* = 152.6 Hz, C-P), 52.9 (d, *J* = 7.3 Hz, (CH_3_O)_2_P), 53.5 (d, *J* = 6.8 Hz, (CH_3_O)_2_P), 123.4 (C=C), 126.4 (C=C), 127.3, 129.0, 136.4, 139.9 (C=C), 142.1 (C=C), 166.3 (d, *J* = 5.4 Hz, C=O). ^31^P NMR (CDCl_3_, 202 MHz): *δ* 26.7 ppm.

Dimethyl *N*-[*N*-methylpyrrol-2-ene-1-oxo]-2-(methylpropyl)phosphonate (**3i**). Phosphonoacetamide **7a** (0.20 g, 0.6 mmol), lithium chloride (70 mg, 1.6 mmol), DBU (0.27 g, 0.27 mL, 1.7 mmol) and *N*-methyl-2-pyrrolecarboxaldehyde (60 mg, 0.06 mL, 0.6 mmol) were reacted, obtaining the product **3i** (80% yield, only the diastereoisomer *E*) as a brown solid, Mp: 125 °C. ^1^H NMR (CDCl_3_, 500 MHz): *δ* 1.04 (dd, *J* = 6.9, 1.4 Hz, 3H, (CH_3_)_2_CH), 1.06 (d, *J* = 6.9 Hz, 3H, (CH_3_)_2_CH), 2.25 (oct, *J* = 6.8 Hz, 1H, CH(CH_3_)_2_), 3.70 (s, 3H, CH_3_-N), 3.75 (d, *J* = 10.7 Hz, 3H, (CH_3_O)_2_P), 3.80 (d, *J* = 10.7 Hz, 3H, (CH_3_O)_2_P), 4.61 (ddd, *J* = 18.3, 10.5, 4.4 Hz, 1H, CH-P), 6.15–6.16 (m, 1H, H_arom_), 6.22 (d, *J* = 10.3 Hz, 1H, NH), 6.27 (d, *J_trans_* = 15.3 Hz, 1H, CH=CH), 6.61–6.62 (m, 1H, H_arom_), 6.71–6.72 (m, 1H, H_arom_), 7.61 (d, *J_trans_* = 15.3 Hz, 1H, CH=CH). ^13^C NMR (CDCl_3_, 125 MHz): *δ* 18.0 (d, *J* = 5.0 Hz, (CH_3_)_2_CH), 20.4 (d, *J* = 12.7 Hz, (CH_3_)_2_CH), 29.2 (d, *J* = 4.1 Hz, CH(CH_3_)_2_), 34.3 (CH_3_-N), 49.6 (d, *J* = 152.1 Hz, C-P), 52.8 (d, *J* = 6.8 Hz, (CH_3_O)_2_P), 53.2 (d, *J* = 7.3 Hz, (CH_3_O)_2_P), 109.1, 110.6, 114.7 (C=C), 126.3, 129.5, 129.9 (C=C), 166.6 (d, *J* = 5.9 Hz, C=O). ^31^P NMR (CDCl_3_, 202 MHz): *δ* 26.8 ppm. HRMS-CI(+) *m/z*: [M + H]^+^ Calcd for C_14_H_23_N_2_O_4_P + H^+^ 315.1468; Found 315.1481.

Dimethyl *N*-[5-methylhex-2-ene-1-oxo]-2-(methylpropyl)phosphonate (**3j**). Phosphonoacetamide **7a** (0.20 g, 0.6 mmol), lithium chloride (70 mg,1.6 mmol), DBU (0.27 g, 0.27 mL, 1.7 mmol) and isovaleraldehyde (60 mg, 0.06 mL, 0.6 mmol) were reacted, obtaining the product **3j** (80% yield, only the diastereoisomer *E*) as a white solid, Mp: 125 °C. ^1^H NMR (CDCl_3_, 500 MHz): *δ* 0.92 (d, *J =* 6.6 Hz, 3H, (CH_3_)_2_CH), 0.93 (d, *J =* 6.7 Hz, 3H, (CH_3_)_2_CH), 1.01 (dd, *J* = 6.8, 1.3 Hz, 3H, (CH_3_)_2_CH), 1.04 (d, *J* = 6.9 Hz, 3H, (CH_3_)_2_CH), 1.76 (sept, *J* = 6.7 Hz, 1H, CH(CH_3_)_2_), 2.08 (t, *J =* 7.9 Hz, 2H, CH_2_CH), 2.27 (oct, *J* = 6.8 Hz, 1H, CH(CH_3_)_2_), 3.73 (d, *J* = 10.7 Hz, 3H, (CH_3_O)_2_P), 3.79 (d, *J* = 10.7 Hz, 3H, (CH_3_O)_2_P), 4.54 (ddd, *J* = 18.0, 10.4, 4.6 Hz, 1H, CH-P), 5.96 (d, *J_trans_* = 15.6 Hz, 1H, CH=CH), 6.55 (d, *J* = 10.4 Hz, 1H, NH), 6.89 (dt, *J_trans_* = 15.0, 7.9 Hz, 1H, CH=CH). ^13^C NMR (CDCl_3_, 125 MHz): *δ* 18.3 (d, *J* = 5.4 Hz, (CH_3_)_2_CH), 20.5 (d, *J =* 12.3 Hz, (CH_3_)_2_CH), 22.6 (d, *J* = 4.5 Hz, (CH_3_)_2_CH), 28.0 (d, *J* = 4.5 Hz, (CH_3_)_2_CH), 29.3 (d, *J* = 4.5 Hz, CH(CH_3_)_2_), 29.8 (d, *J =* 12.3 Hz, CH(CH_3_)_2_), 41.7 (CH_2_CH), 49.6 (d, *J =* 152.6 Hz, C-P), 52.8 (d, *J* = 6.8 Hz, (CH_3_O)_2_P), 53.4 (d, *J* = 6.8 Hz, (CH_3_O)_2_P), 124.1 (C=C), 145.0 (C=C), 166.2 (d, *J* = 5.5 Hz, C=O). ^31^P NMR (CDCl_3_, 202 MHz): *δ* 26.7 ppm.

Dimethyl *N*-[4-methylpent-2-ene-1-oxo]-2-(methylpropyl)phosphonate (**3k**). Phosphonoacetamide **7a** (0.20 g, 0.6 mmol), lithium chloride (70 mg, 1.6 mmol), DBU (0.27 g, 0.27 mL, 1.7 mmol), and isobutyraldehyde (40 mg, 0.05 mL, 0.6 mmol) were reacted, obtaining the product **3k** (65% yield, *E*/*Z* 85:15 ratio) as a white solid, Mp: 100–102 °C. ^1^H NMR (CDCl_3_, 500 MHz): *δ* 1.02 (d, *J* = 6.7 Hz, 3H, (CH_3_)_2_CH), 1.04 (d, *J* = 6.8 Hz, 3H, (CH_3_)_2_CH), 1.07 (d, *J* = 6.7 Hz, 6H, (CH_3_)_2_CH), 2.23 (oct, *J* = 6.8 Hz, 1H, CH(CH_3_)_2_), 2.46 (sept, *J* = 6.7 Hz, 1H, CH(CH_3_)_2_), 3.73 (d, *J* = 10.7 Hz, 3H, (CH_3_O)_2_P), 3.78 (d, *J* = 10.7 Hz, 3H, (CH_3_O)_2_P), 4.55 (ddd, *J* = 18.3, 10.5, 4.4 Hz, 1H, CH-P), 5.84 (d, *J_trans_* = 15.4 Hz, 1H, CH=CH), 6.09 (d, *J* = 10.5 Hz, 1H, NH), 6.89 (dd, *J_trans_* = 15.4, 6.5 Hz, 1H, CH=CH). ^13^C NMR (CDCl_3_, 125 MHz): *δ* 18.2 (d, *J* = 4.3 Hz, (CH_3_)_2_CH), 20.6 (d, *J* = 12.6 Hz, (CH_3_)_2_CH), 21.6 (d, *J* = 5.4 Hz, (CH_3_)_2_CH), 27.7 (d, *J* = 12.3 Hz, (CH_3_)_2_CH), 29.3 (d, *J* = 3.6 Hz, CH(CH_3_)_2_), 31.0 (d, *J* = 3.6 Hz, CH(CH_3_)_2_), 49.6 (d, *J* = 152.6 Hz, C-P), 52.9 (d, *J* = 6.8 Hz, (CH_3_O)_2_P), 53.3 (d, *J* = 6.8 Hz, (CH_3_O)_2_P), 120.3 (C=C), 152.5 (C=C), 166.3 (d, *J* = 5.8 Hz, C=O). ^31^P NMR (CDCl_3_, 202 MHz): *δ* 26.8 ppm.

Dimethyl *N*-[4,4-dimethylpent-2-ene-1-oxo]-2-(methylpropyl)phosphonate (**3l**). Phosphonoacetamide **7a** (0.20 g, 0.6 mmol), lithium chloride (70 mg, 1.6 mmol), DBU (0.27 g, 0.27 mL, 1.7 mmol), and trimethylacetaldehyde (50 mg, 0.06 mL, 0.6 mmol) were reacted, obtaining the product **3l** (54% yield, only the diastereoisomer *E*) as a white solid, Mp: 104–105 °C. ^1^H NMR (CDCl_3_, 500 MHz): *δ* 1.02 (dd, *J* = 6.9, 1.4 Hz, 3H, (CH_3_)_2_CH), 1.05 (d, *J* = 7.0 Hz, 3H, (CH_3_)_2_CH), 1.09 (s, 9H, (CH_3_)_3_C), 2.23 (oct, *J* = 6.8 Hz, 1H, CH(CH_3_)_2_), 3.73 (d, *J* = 10.7 Hz, 3H, (CH_3_O)_2_P), 3.78 (d, *J* = 10.7 Hz, 3H, (CH_3_O)_2_P), 4.56 (ddd, *J =* 18.3, 9.9, 4.5 Hz, 1H, CH-P), 5.84 (d, *J_tran_*_s_ = 15.6 Hz, 1H, CH=CH), 6.33 (d, *J* = 9.9 Hz, 1H, NH), 6.91 (d, *J_trans_ =* 15.6 Hz, 1H, CH=CH). **^13^**C NMR (CDCl_3_, 125 MHz): *δ* 18.3 (d, *J* = 5.0 Hz, (CH_3_)_2_CH), 20.6 (d, *J* = 12.3 Hz, (CH_3_)_2_CH), 29.0 ((CH_3_)_3_C), 29.3 (d, *J* = 4.1 Hz, CH(CH_3_)_2_), 33.8 (C(CH_3_)_3_), 49.6 (d, *J* = 152.6 Hz, C-P), 52.8 (d, *J* = 6.8 Hz, (CH_3_O)_2_P), 53.5 (d, *J* = 6.8 Hz, (CH_3_O)_2_P), 118.4 (C=C), 155.9 (C=C), 166.8 (d, *J* = 5.9 Hz, C=O). ^31^P NMR (CDCl_3_, 202 MHz): *δ* 26.7 ppm.

Dimethyl *N*-[1-(3-cyclohex-2-ene-1-oxo)-2-(methylpropyl)phosphonate (**3m**). Phosphonoacetamide **7a** (0.20 g, 0.6 mmol), lithium chloride (70 mg, 1.6 mmol), DBU (0.27 g, 0.27 mL, 1.7 mmol), and 1-cyclohexenecarboxaldehyde (60 mg, 0.07 mL, 0.6 mmol) were reacted, obtaining the product **3m** (80% yield, only the diastereoisomer *E*) as a yellow liquid. ^1^H NMR (CDCl_3_, 500 MHz): *δ* 1.01 (dd, *J* = 6.9, 1.4 Hz, 3H, (CH_3_)_2_CH), 1.03 (d, *J* = 6.9 Hz, 3H, (CH_3_)_2_CH), 1.58–1.66 (m, 2H, CH_2_), 1.67–1.73 (m, 2H, CH_2_), 2.12–2.16 (m, 2H, CH_2_), 2.17–2.26 (m, 3H, CH_2_ and CH(CH_3_)_2_), 3.72 (d, *J* = 10.7 Hz, 3H, (CH_3_O)_2_P), 3.77 (d, *J* = 10.7 Hz, 3H, (CH_3_O)_2_P), 5.84 (d, *J_trans_* = 15.0 Hz, 1H, CH=CH), 5.99 (d, *J* = 9.3 Hz, 1H, NH), 6.14–6.15 (m, 1H, CH-CH_2_), 6.91 (d, *J_trans_* = 15.0 Hz, 1H, CH=CH). ^13^C NMR (CDCl_3_, 125 MHz): *δ* 18.2 (d, *J* = 4.5 Hz, (CH_3_)_2_CH), 20.6 (d, *J* = 12.7 Hz, (CH_3_)_2_CH), 22.3 (d, *J* = 5.4 Hz, CH(CH_3_)_2_), 24.5, 26.6, 29.3 (2C), 49.6 (d, *J* = 152.1 Hz, C-P), 52.9 (d, *J* = 6.8 Hz, (CH_3_O)_2_P), 53.4 (d, *J* = 6.8 Hz, (CH_3_O)_2_P), 116.3 (C=C), 134.8 (C=C), 138.3 (C=C), 145.7 (C=C), 166.8 (d, *J* = 5.8 Hz, C=O). ^31^P NMR (CDCl_3_, 202 MHz): *δ* 26.8 ppm.

Dimethyl *N*-[3-phenyl-2-ene-1-oxo]-(phenyl-methyl)phosphonate (**8a**). Phosphonoacetamide **7b** (0.20 g, 0.6 mmol), lithium chloride (70 mg, 1.6 mmol), DBU (0.27 g, 0.27 mL, 1.7 mmol) and benzaldehyde (60 mg, 0.06 mL, 0.6 mmol) were reacted, obtaining the product **8a** (80% yield, only the diastereoisomer *E*) as a white solid, Mp: 165–167 °C. ^1^H NMR (CDCl_3_, 600 MHz): *δ* 3.53 (d, *J =* 10.7 Hz, 3H, (CH_3_O)_2_P), 3.85 (d, *J =* 10.8 Hz, 3H, (CH_3_O)_2_P), 5.84 (dd, *J =* 20.8, 9.8 Hz, 1H, CH-P), 6.68 (d, *J_trans_ =* 15.7 Hz, 1H, CH=CH), 7.31–7.24 (m, 4H, H_arom_), 7.33 (t, *J* = 7.8 Hz, 2H, H_arom_), 7.40–7.37 (m, 2H, H_arom_), 7.63–7.59 (m, 2H, H_arom_), 7.63 (d, *J_trans_ =* 15.7 Hz, 1H, CH=CH), 8.43 (d, *J =* 9.8 Hz, 1H, NH). ^13^C NMR (CDCl_3_, 151 MHz): *δ* 49.7 (d, *J =* 154.9 Hz, C-P), 53.9 (d, *J =* 7.0 Hz, (CH_3_O)_2_P), 53.9 (d, *J =* 7.1 Hz, (CH_3_O)_2_P), 120.7 (C=C), 127.8 (2C), 128.4 (2C), 128.5, 128.7 (2C), 128.8 (2C), 129.7, 135.0, 135.1, 141.5 (C=C), 165.7 (d, *J =* 7.9 Hz, C=O). ^31^P NMR (CDCl_3_, 243 MHz): *δ* 24.0 ppm. HRMS-CI(+) *m/z*: [M + H]^+^ Calcd for C_18_H_20_NO_4_P + H^+^ 346.1203; Found 346.1218.

HRMS-CI(+) *m/z*: [M + H]^+^ Calcd for C_13_H_22_NO_7_P_2_ +H^+^ 366.0866; Found 366.0871.

Dimethyl *N*-[3-(4-methoxyphenyl)-2-ene-1-oxo]-(phenyl-methyl)phosphonate (**8b**). Phosphonoacetamide **7b** (0.20 g, 0.6 mmol), lithium chloride (70 mg, 1.6 mmol), DBU (0.27 g, 0.27 mL, 1.7 mmol) and *4-*methoxybenzaldehyde (70 mg, 0.06 mL, 0.6 mmol) were reacted, obtaining the product **8b** (50% yield, only the diastereoisomer *E*) as a white solid, Mp: 163–165 °C. ^1^H NMR (CDCl_3_, 500 MHz): *δ* 3.52 (d, *J* = 10.6 Hz, 3H, (CH_3_O)_2_P), 3.81 (s, 3H, CH_3_O), 3.83 (d, *J* = 10.7 Hz, 3H, (CH_3_O)_2_P), 5.78 (dd, *J* = 20.8, 9.6 Hz, 1H, CH-P), 6.47 (d, *J_trans_* = 15.6 Hz, 1H, CH=CH), 6.84 (AA’BB’ system, *J* = 8.8 Hz, 2H, H_arom_), 7.29 (d, *J* = 7.4 Hz, 1H, H_arom_), 7.32–7.35 (m, 2H, H_arom_), 7.37 (d, *J* = 9.2 Hz, 2H, H_arom_), 7.57 (AA’BB’ system, *J* = 8.9 Hz, 2H, H_arom_), 7.59 (d, *J_trans_* = 15.7 Hz, 1H, CH=CH), 7.63 (d, *J* = 9.6 Hz, 1H, NH). ^13^C NMR (CDCl_3_, 125 MHz): *δ* 49.8 (d, *J* = 154.4 Hz, C-P), 54.0 (d, *J* = 8.6 Hz, (CH_3_O)_2_P), 54.0 (d, *J* = 7.7 Hz, (CH_3_O)_2_P), 55.5 (CH_3_O), 114.4 (2C), 118.1 (C=C), 127.8, 128.5 (2C), 129.0, 129.6 (2C), 135.2, 141.7 (C=C), 161.2, 165.9 (d, *J* = 7.7 Hz, C=O). ^31^P NMR (CDCl_3_, 202 MHz): *δ* 24.1 ppm. HRMS-CI(+) *m/z*: [M + H]^+^ Calcd for C_19_H_22_NO_5_P + H^+^ 376.1308; Found 376.1314.

Dimethyl *N*-[3-(4-chlorophenyl)-2-ene-1-oxo]-(phenyl-methyl)phosphonate (**8c**). Phosphonoacetamide **7b** (0.20 g, 0.6 mmol), lithium chloride (70 mg, 1.6 mmol), DBU (0.25 g, 0.24 mL, 1.7 mmol) and *4-*chlorobenzaldehyde (70 mg, 0.6 mmol) were reacted, obtaining the product **8c** (80% yield, only the diastereoisomer *E*) as a white solid, Mp: 151–153 °C. ^1^H NMR (CDCl_3_, 600 MHz): *δ* 3.52 (d, *J* = 10.6 Hz, 3H, (CH_3_O)_2_P), 3.85 (d, *J* = 10.8 Hz, 3H, (CH_3_O)_2_P), 5.81 (dd, *J* = 20.7, 9.8 Hz, 1H, CH-P), 6.61 (d, *J_trans_* = 15.6 Hz, 1H, CH=CH), 7.36–7.26 (m, 7H, H_arom_), 7.57 (d, *J_trans_* = 15.6 Hz, 1H, CH=CH), 7.58 (AA’BB’ system, *J* = 7.7 Hz, 2H, H_arom_), 8.21 (d, *J* = 9.7 Hz, 1H, NH). ^13^C NMR (CDCl_3_, 151 MHz): *δ* 49.7 (d, *J* = 155.0 Hz, C-P), 53.9 (d, *J* = 7.2 Hz, (CH_3_O)_2_P), 53.9 (d, *J* = 7.2 Hz, (CH_3_O)_2_P), 121.2 (C=C), 128.3, 128.4 (2C), 128.5, 128.9 (2C), 129.0 (2C), 129.1 (2C), 133.6, 134.9, 135.5, 140.2 (C=C), 165.3 (d, *J* = 7.6 Hz, C=O). ^31^P NMR (CDCl_3_, 243 MHz): *δ* 24.7 ppm. HRMS-CI(+) *m/z*: [M + H]^+^ Calcd for C_18_H_20_ ClNO_4_P + H^+^ 380.0813; Found 380.0786.

Dimethyl *N*-[3-(4-fluorophenyl)-2-ene-1-oxo]-(phenyl-methyl)phosphonate (**8d**). Phosphonoacetamide **7b** (0.20 g, 0.6 mmol), lithium chloride (70 mg, 1.6 mmol), DBU (0.25 g, 0.24 mL, 1.7 mmol) and *4-*fluorobenzaldehyde (70 mg, 0.6 mmol) were reacted, obtaining the product **8d** (80% yield, only the diastereoisomer *E*) as a white solid, Mp: 145–146 °C. ^1^H NMR (CDCl_3_, 500 MHz): *δ* 3.52 (d, *J* = 10.7 Hz, 3H, (CH_3_O)_2_P), 3.85 (d, *J* = 10.8 Hz, 3H, (CH_3_O)_2_P), 5.82 (ddd, *J* = 20.8, 9.8, 4.6 Hz, 1H, CH), 6.57 (dd, *J_trans_* = 15.7, 5.6 Hz, 1H, CH=CH), 6.97–7.00 (m, 2H, H_arom_), 7.26–7.37 (m, 5H, H_arom_), 7.57–7.60 (m, 2H, H_arom_), 8.24 (d, *J* = 9.8 Hz, 1H, NH). ^13^C NMR (CDCl_3_, 125 MHz): *δ* 49.8 (d, *J* = 154.9 Hz, C-P), 54.0 (d, *J* = 7.3 Hz, (CH_3_O)_2_P), 54.1 (d, *J* = 7.3 Hz, (CH_3_O)_2_P), 116.0 (2C, *J =* 21.3 Hz), 120.5 (C=C), 128.5 (2C), 129.0 (2C), 129.7 (2C, *J* = 8.6 Hz), 131.4, 135.0, 140.5 (C=C), 140.5, 163.7 (d, *J* = 250.2 Hz, C-F), 165.6 (C=O). ^31^P NMR (CDCl_3_, 202 MHz): *δ* 24.0 ppm. HRMS-CI(+) *m/z*: [M + H]^+^ Calcd for C_18_H_20_FNO_4_P + H^+^ 364.1108; Found 364.1121.

Dimethyl *N*-[3-(4-trifluoromethyl)phenyl)-2-ene-1-oxo]-(phenyl-methyl)phosphonate (**8e**). Phosphonoacetamide **7b** (0.20 g, 0.6 mmol), lithium chloride (70 mg, 1.6 mmol), DBU (0.25 g, 0.24 mL, 1.7 mmol) and *4-*trifluoromethylbenzaldehyde (90 mg, 0.07 mL, 0.6 mmol) were reacted, obtaining the product **8e** (85% yield, only the diastereoisomer *E*) as a white solid, Mp: 153–154 °C. ^1^H NMR (CDCl_3_, 500 MHz): *δ* 3.54 (d, *J* = 10.5 Hz, 3H, (CH_3_O)_2_P), 3.88 (d, *J* = 10.8 Hz, 3H, (CH_3_O)_2_P), 5.85 (dd, *J* = 20.8, 9.8 Hz, 1H, CH-P), 6.75 (d, *J_trans_* = 15.7 Hz, 1H, CH=CH), 7.25–7.28 (m, 1H, H_arom_), 7.33 (dd, *J* = 8.1 Hz, 2H, H_arom_), 7.46 (d, *J* = 8.5 Hz, 2H, H_arom_), 7.54 (AA’BB’ system, *J* = 8.4 Hz, 2H, H_arom_), 7.61–7.63 (m, 2H, H_arom_), 7.63 (d, *J_trans_* = 15.7 Hz, 1H, CH=CH), 8.56 (d, *J* = 9.8 Hz, 1H, NH). ^13^C NMR (CDCl_3_, 125 MHz): *δ* 49.6 (d, *J* = 154.4 Hz, C-P), 53.9 (d, *J* = 7.3 Hz, (CH_3_O)_2_P), 53.9 (d, *J* = 7.3 Hz, (CH_3_O)_2_P), 123.2 (C=C), 123.9 (q, *J* = 272.0 Hz, CF_3_), 125.6 (2C, *J* = 4.1 Hz), 127.9 (3C), 128.3, 128.4, 128.8, 131.1 (q, *J* = 32.3 Hz, C-CF_3_), 134.7, 138.5, 139.6 (C=C), 165.0 (d, *J* = 7.3 Hz, C=O). ^31^P NMR (CDCl_3_, 202 MHz): δ 23.8 ppm.

Dimethyl *N*-[(2*E,*4*E*)-5-phenylpenta-2,4-dien-1-oxo]-(phenyl-methyl)phosphonate (**8f**). Phosphonoacetamide **7b** (0.20 g, 0.5 mmol), lithium chloride (70 mg, 1.6 mmol), DBU (0.25 g, 0.24 mL, 1.7 mmol) and *trans*-cinnamaldehyde (70 mg, 0.07 mL, 0.6 mmol) were reacted, obtaining the product **8f** (85% yield, only the diastereoisomer *E*) as a yellow solid, Mp: 75–76 °C. ^1^H NMR (CDCl_3_, 500 MHz): *δ* 3.53 (d, *J* = 10.7 Hz, 3H, (CH_3_O)_2_P), 3.85 (d, *J* = 10.8 Hz, 3H, (CH_3_O)_2_P), 5.82 (dd, *J* = 20.9, 9.8 Hz, 1H, CH-P), 6.19 (d, *J_trans_* = 15.0 Hz, 1H, CH=CH), 6.74 (d, *J_trans_* = 15.6 Hz, 1H, CH), 6.83 (d, *J_trans_ =* 15.6 Hz, 1H, CH=CH), 7.38–7.27 (m, 6H, H_arom_), 7.46–7.39 (m, 2H, H_arom_), 7.58–7.60 (m, 2H, CH=CH and H_arom_), 8.11 (d, *J* = 9.8 Hz, 1H, NH). ^13^C NMR (CDCl_3_, 125 MHz): *δ* 49.8 (d, *J* = 154.9 Hz, C-P), 54.0 (d, *J* = 7.6 Hz, (CH_3_O)_2_P), 54.1 (d, *J* = 7.7 Hz, (CH_3_O)_2_P), 123.7 (C=C), 126.6 (C=C), 127.2, 128.5, 128.6 (2C), 129.0 (4C), 135.1, 136.5, 139.6 (C=C), 141.9 (C=C), 165.9 (d, *J* = 7.7 Hz, C=O). ^31^P NMR (CDCl_3_, 202 MHz): *δ* 24.0 ppm.

Dimethyl *N*-[*N*-methylpyrrol-2-ene-1-oxo]-(phenyl-methyl)phosphonate (**8g**). Phosphonoacetamide **7b** (0.20 g, 0.5 mmol), lithium chloride (70 mg, 1.6 mmol), DBU (0.25 g, 0.24 mL, 1.7 mmol) and *N*-methyl-2-pyrrolecarboxaldehyde (60 mg, 0.06 mL, 0.6 mmol) were reacted, obtaining the product **8g** (85% yield, only the diastereoisomer *E*) as a white solid, Mp: 143–144 °C. ^1^H NMR (CDCl_3_, 500 MHz): *δ* 3.52 (d, *J* = 10.5 Hz, 3H, (CH_3_O)_2_P), 3.64 (s, 3H, CH_3_-N), 3.82 (d, *J* = 10.7 Hz, 3H, (CH_3_O)_2_P), 5.78 (dd, *J* = 20.9, 9.6 Hz, 1H, CH-P), 6.10–6.11 (m, 1H, H_arom._), 6.33 (d, *J_trans_* = 15.4 Hz, 1H, CH=CH), 6.48–6.49 (m, 1H, H_arom._), 6.67–6.68 (m, 1H, H_arom._), 7.28–7.30 (m, 1H, H_arom_), 7.33–736 (m, 2H, H_arom_), 7.55–7.58 (m, 3H, CH=CH, H_arom_), 7.71 (d, *J* = 9.7, 1H, NH). ^13^C NMR (CDCl_3_, 125 MHz): *δ* 34.5 (CH_3_-N), 49.9 (d, *J* = 154.9 Hz, C-P), 54.0 (d, *J* = 7.7 Hz, (CH_3_O)_2_P), 54.1 (d, *J* = 7.7 Hz, (CH_3_O)_2_P), 109.2, 110.8, 115.3 (C=C), 126.4, 128.5 (3C), 129.0 (2C), 129.8, 129.9 (C=C), 135.3, 166.4 (d, *J* = 7.7 Hz, C=O). ^31^P NMR (CDCl_3_, 202 MHz): δ 24.1 ppm.

Dimethyl *N*-[4-methylpent-2-ene-1-oxo]-(phenyl-methyl)phosphonate (**8h**). Phosphonoacetamide **7b** (0.20 g, 0.5 mmol), lithium chloride (70 mg, 1.6 mmol), DBU (0.25 g, 0.24 mL, 1.7 mmol) and isobutyraldehyde (40 mg, 0.05 mL, 0.6 mmol) were reacted, obtaining the product **8h** (80% yield, only the diastereoisomer *E*) as a white solid, Mp: 116–118 °C. ^1^H NMR (CDCl_3_, 500 MHz): *δ* 0.99 (d, *J* = 6.8 Hz, 3H, (CH_3_)_2_CH), 1.00 (d, *J* = 6.8 Hz, 3H, (CH_3_)_2_CH), 2.37 (oct, *J* = 6.7 Hz, 1H, CH(CH_3_)_2_), 3.49 (d, *J* = 10.6 Hz, 3H, (CH_3_O)_2_P), 3.82 (d, *J* = 10.6 Hz, 3H, (CH_3_O)_2_P), 5.72 (dd, *J* = 20.9, 9.5 Hz, 1H, CH-P), 5.92 (dd, *J_trans_* = 15.4, 1.5 Hz, 1H, CH=CH), 6.88 (dd, *J_trans_* = 15.4, 6.8 Hz, 1H, CH=CH), 7.37–7.39 (m, 3H, H_arom_), 7.57–7.52 (m, 2H, H_arom_), 7.78 (d, *J* = 9.5 Hz, 1H, NH). ^13^C NMR (CDCl_3_, 125 MHz): *δ* 21.4 ((CH_3_)_2_CH), 21.5 ((CH_3_)_2_CH), 30.9 (CH(CH_3_)_2_), 49.6 (d, *J* = 154.9 Hz, C-P), 53.9 (d, *J* = 9.5 Hz, (CH_3_O)_2_P), 53.9 (d, *J* = 6.8 Hz, (CH_3_O)_2_P), 120.5 (C=C), 128.4, 128.6 (2C), 128.9 (2C), 135.2, 152.2 (C=C), 165.9 (d, *J* = 7.7 Hz, C=O). ^31^P NMR (CDCl_3_, 202 MHz): *δ* 24.0 ppm.

Dimethyl *N*-[4,4-dimethylpent-2-ene-1-oxo]-(phenyl-methyl)phosphonate (**8i**). Phosphonoacetamide **7b** (0.20 g, 0.5 mmol), lithium chloride (70 mg, 1.6 mmol), DBU (0.25 g, 0.24 mL, 1.7 mmol) and trimethylacetaldehyde (50 mg, 0.06 mL, 0.6 mmol) were reacted, obtaining the product **8i** (85% yield, only the diastereoisomer *E*) as a white solid, Mp: 118–120 °C. ^1^H NMR (CDCl_3_, 500 MHz): *δ* 1.01 (s, 9H, (CH_3_)_3_C), 3.49 (d, *J* = 10.7 Hz, 3H, (CH_3_O)_2_P), 3.82 (d, *J* = 10.8 Hz, 3H, (CH_3_O)_2_P), 5.73 (dd, *J* = 20.9, 8.1 Hz, 1H, CH-P), 5.88 (d, *J_trans_* = 15.8 Hz, 1H, CH=CH), 6.89 (d, *J_trans_* = 15.5 Hz, 1H, CH=CH), 7.37–7.28 (m, 3H, H_arom_), 7.53–7.55 (m, 2H, H_arom_). ^13^C NMR (CDCl_3_, 125 MHz): *δ* 28.9 ((CH_3_)_3_C), 33.7 (C(CH_3_)_3_), 49.6 (d, *J* = 155.3 Hz, C-P), 53.9 (d, *J* = 7.3 Hz, (CH_3_O)_2_P), 54.0 (d, *J* = 7.3 Hz, (CH_3_O)_2_P), 118.5 (C=C), 128.4, 128.6 (2C), 128.9 (2C), 135.3, 155.9 (C=C), 166.1 (C=O). ^31^P NMR (CDCl_3_, 202 MHz): δ 24.1 ppm. HRMS-CI(+) *m/z*: [M + H]^+^ Calcd for C_16_H_25_NO_4_P + H^+^ 326.1516; Found 326.1536.

Dimethyl *N*-[3-phenyl-2-ene-1-oxo]-(4-methoxyphenyl-methyl)phosphonate (**9a**). Phosphonoacetamide **7c** (0.20 g, 0.5 mmol), lithium chloride (60 mg, 1.6 mmol), DBU (0.23 g, 0.23 mL, 1.6 mmol) and benzaldehyde (50 mg, 0.05 mL, 0.6 mmol) were reacted, obtaining the product **9a** (93% yield, only the diastereoisomer *E*) as a white solid, Mp: 134–135 °C. ^1^H NMR (CDCl_3_, 500 MHz): *δ* 3.54 (d, *J* = 10.5 Hz, 3H, (CH_3_O)_2_P), 3.71 (s, 3H, CH_3_O), 3.84 (d, *J* = 10.8 Hz, 3H, (CH_3_O)_2_P), 5.77 (dd, *J* = 20.4, 9.7 Hz, 1H, CH-P), 6.65 (d, *J_trans_* = 15.7 Hz, 1H, CH=CH), 6.85–6.90 (m, 3H, H_arom_), 7.29–7.31 (m, 3H, H_arom_), 7.39–7.41 (m, 2H, H_arom_), 7.51 (d, *J* = 8.7 Hz, 1H, H_arom_), 7.63 (d, *J_trans_* = 15.6 Hz, 1H, CH=CH), 8.17 (d, *J* = 9.8 Hz, 1H, NH). ^13^C NMR (CDCl_3_, 125 MHz): *δ* 49.1 (d, *J =* 156.2 Hz, C-P), 54.0 (d, *J =* 6.8 Hz, (CH_3_O)_2_P), 54.0 (d, *J =* 6.8 Hz, (CH_3_O)_2_P), 55.4 (CH_3_O), 114.4 (3C), 120.8 (C=C), 127.0, 128.0 (2C), 128.9 (2C), 129.8 (2C), 135.2, 141.6 (C=C), 159.7, 165.7 (d, *J* = 7.7 Hz, C=O). ^31^P NMR (CDCl_3_, 202 MHz): *δ* 23.0 ppm.

Dimethyl *N*-[3-(4-methoxyphenyl)-2-ene-1-oxo]-(4-methoxyphenyl-methyl)phosphonate (**9b**). Phosphonoacetamide **7c** (0.20 g, 0.5 mmol), lithium chloride (60 mg, 1.6 mmol), DBU (0.23 g, 0.23 mL, 1.6 mmol) and *4-*methoxybenzaldehyde (90 mg, 0.08 mL, 0.5 mmol) were reacted, obtaining the product **9b** (90% yield, only the diastereoisomer *E*) as a white solid, Mp: 137–138 °C. ^1^H NMR (CDCl_3_, 500 MHz): *δ* 3.56 (d, *J* = 10.5 Hz, 3H, (CH_3_O)_2_P), 3.68 (s, 3H, CH_3_O), 3.78 (s, 3H, CH_3_O), 3.84 (d, *J* = 10.8 Hz, 3H, (CH_3_O)_2_P), 5.81 (dd, *J* = 20.5, 9.8 Hz, 1H, CH-P), 6.57 (d, *J_trans_* = 15.6 Hz, 1H, CH=CH), 6.80 (AA’BB’ system, *J* = 8.7 Hz, 2H, H_arom_), 6.83 (AA’BB’ system, *J* = 8.7 Hz, 2H, H_arom_), 7.32 (AA’BB’ system, *J* = 8.9 Hz, 2H, H_arom_), 7.53 (AA’BB’ system, *J* = 8.8, 2.1 Hz, 2H, H_arom_), 7.59 (d, *J_trans_* = 15.7 Hz, 1H, CH=CH), 8.51 (d, *J* = 9.8 Hz, 1H, NH). ^13^C NMR (CDCl_3_, 125 MHz): *δ* 49.1 (d, *J* = 156.2 Hz, C-P), 53.9 (d, *J* = 7.7 Hz, (CH_3_O)_2_P), 54.0 (d, *J* = 7.8 Hz, (CH_3_O)_2_P), 55.4 (CH_3_O), 55.5 (CH_3_O), 114.3 (3C), 118.6 (C=C), 127.2, 128.0, 129.5 (3C), 129.8 (2C), 141.1 (C=C), 159.7, 161.0, 166.2 (d, *J* = 7.7 Hz, C=O). ^31^P NMR (CDCl_3_, 202 MHz): δ 24.3 ppm. HRMS-CI(+) *m/z*: [M + H]^+^ Calcd for C_20_H_25_NO_6_P + H^+^ 406.1414; Found 406.1442.

Dimethyl *N*-[3-(4-chlorophenyl)-2-ene-1-oxo]-(4-methoxyphenyl-methyl)phosphonate (**9c**). Phosphonoacetamide **7c** (0.20 g, 0.5 mmol), lithium chloride (60 mg, 1.6 mmol), DBU (0.23 g, 0.23 mL, 1.6 mmol) and *4-*chlorobenzaldehyde (90 mg, 0.5 mmol) were reacted, obtaining the product **9c** (85% yield, only the diastereoisomer *E*) as a white solid, Mp: 156–157 °C. ^1^H NMR (CDCl_3_, 500 MHz): *δ* 3.53 (d, *J* = 10.5 Hz, 3H, (CH_3_O)_2_P), 3.73 (s, 3H, CH_3_O), 3.84 (d, *J* = 10.8 Hz, 3H, (CH_3_O)_2_P), 5.73 (dd, *J* = 20.4, 9.7 Hz, 1H, CH-P), 6.58 (d, *J_trans_* = 15.6 Hz, 1H, CH=CH), 6.86 (AA’BB’ system, *J* = 8.4 Hz, 2H, H_arom_), 7.28 (AA’BB’ system, *J* = 8.7 Hz, 2H, H_arom_), 7.33 (AA’BB’ system, *J* = 8.5 Hz, 2H, H_arom_), 7.49 (AA’BB’ system, *J* = 8.7, 2.0 Hz, 2H, H_arom_), 7.57 (d, *J_trans_* = 15.7 Hz, 1H, CH=CH), 7.96 (d, *J* = 9.7 Hz, 1H, NH). ^13^C NMR (CDCl_3_, 125 MHz): *δ* 49.1 (d, *J* = 156.2 Hz, C-P), 54.0 (d, *J* = 7.8 Hz, (CH_3_O)_2_P), 54.1 (d, *J* = 7.7 Hz, (CH_3_O)_2_P), 55.4 (CH_3_O), 114.4 (2C), 121.4 (C=C), 126.9, 129.1 (2C), 129.2 (2C), 129.7 (2C), 133.7, 135.6, 140.3 (C=C), 159.8, 165.4 (d, *J* = 7.7 Hz, C=O). ^31^P NMR (CDCl_3_, 202 MHz): δ 24.1 ppm.

Dimethyl *N*-[3-(4-fluorophenyl)-2-ene-1-oxo]-(4-methoxyphenyl-methyl)phosphonate (**9d**). Phosphonoacetamide **7c** (0.20 g, 0.5 mmol), lithium chloride (60 mg, 1.6 mmol), DBU (0.23 g, 0.23 mL, 1.6 mmol) and *4-*fluorobenzaldehyde (60 mg, 0.05 mL, 0.5 mmol) were reacted, obtaining the product **9d** (62% yield, only the diastereoisomer *E*) as a white solid, Mp: 143–145 °C. ^1^H NMR (CDCl_3_, 500 MHz): *δ* 3.53 (d, *J* = 10.5 Hz, 3H, (CH_3_O)_2_P), 3.73 (s, 3H, CH_3_O), 3.84 (d, *J* = 10.7 Hz, 3H, (CH_3_O)_2_P), 5.74 (dd, *J* = 20.4, 9.7 Hz, 1H, CH-P), 6.54 (d, *J_trans_* = 15.6 Hz, 1H, CH=CH), 6.85 (AA’BB’ system, *J* = 8.5 Hz, 2H, H_arom_), 7.00 (AA’BB’ system, *J* = 8.6 Hz, 2H, H_arom_), 7.38 (AA’BB’ system, *J* = 8.8 Hz, 2H, H_arom_), 7.50 (AA’BB’ system, *J* = 8.8 Hz, 2H, H_arom_), 7.59 (d, *J_trans_* = 15.7 Hz, 1H, CH=CH), 8.00 (d, *J* = 9.7 Hz, 1H, NH). ^13^C NMR (CDCl_3_, 125 MHz): *δ* 49.1 (d, *J* = 156.2 Hz, C-P), 54.0 (d, *J* = 7.3 Hz, (CH_3_O)_2_P), 54.1 (d, *J* = 7.3 Hz, (CH_3_O)_2_P), 55.4 (CH_3_O), 114.4 (2C), 116.0 (2C, *J* = 22.3 Hz), 120.5 (C=C), 127.0, 129.7, 129.8 (2C, *J* = 8.5 Hz), 131.4 (2C, *J* = 3.2 Hz), 140.5 (C=C), 159.8, 163.7 (d, *J* = 250.2 Hz, C-F), 165.5 (d, *J* = 7.7 Hz, C=O). ^31^P NMR (CDCl_3_, 202 MHz): *δ* 24.2 ppm. HRMS-CI(+) *m/z*: [M + H]^+^ Calcd for C_19_H_21_FNO_5_P + H^+^ 394.1214; Found 394.1191.

Dimethyl *N*-[3-(4-(trifluoromethyl)phenyl)-2-ene-1-oxo]-(4-methoxyphenyl-methyl)phosphonate (**9e**). Phosphonoacetamide **7c** (0.20 g, 0.5 mmol), lithium chloride (60 mg, 1.6 mmol), DBU (0.23 g, 0.23 mL, 1.6 mmol) and *4-*trifluoromethylbenzaldehyde (90 mg, 0.07 mL, 0.5 mmol) were reacted, obtaining the product **9e** (50% yield, only the diastereoisomer *E*) as a white solid, Mp: 158–159 °C. ^1^H NMR (CDCl_3_, 500 MHz): δ 3.54 (d, *J* = 10.5 Hz, 3H, (CH_3_O)_2_P), 3.72 (s, 3H, CH_3_O), 3.85 (d, *J* = 10.8 Hz, 3H, (CH_3_O)_2_P), 5.75 (dd, *J* = 20.3, 9.8 Hz, 1H, CH-P), 6.71 (d, *J_trans_* = 15.7 Hz, 1H, CH=CH), 6.86 (AA’BB’ system, *J =* 8.7 Hz, 2H, H_arom_), 7.37–7.40 (m, 4H, H_arom_), 7.56 (AA’BB’ system, *J* = 8.4 Hz, 2H, H_arom_), 7.64 (d, *J_trans_* = 15.7 Hz, 1H, CH=CH), 8.22 (d, *J* = 9.8 Hz, 1H, NH). ^13^C NMR (CDCl_3_, 125 MHz): δ 49.2 (d, *J* = 156.2 Hz, C-P), 54.0 (d, *J* = 7.3 Hz, (CH_3_O)_2_P), 54.1 (d, *J* = 6.8 Hz, (CH_3_O)_2_P), 55.4 (CH_3_O), 114.4 (2C), 123.3 (C=C), 124.1 (q, *J* = 272.0 Hz, CF_3_), 125.9 (2C, *J =* 3.6 Hz), 126.8, 128.1 (2C), 129.8 (2C), 131.4 (q, *J* = 32.2 Hz, C-CF_3_), 138.6, 139.9 (C=C), 159.8, 165.0 (d, *J* = 7.7 Hz, C=O). ^31^P NMR (CDCl_3_, 202 MHz): δ 24.1 ppm. HRMS-CI(+) *m/z*: [M + H]^+^ Calcd for C_20_H_22_F_3_NO_5_P + H^+^ 444.1182; Found 444.1219.

Dimethyl *N*-[4-methylpent-2-ene-1-oxo]-(4-methoxyphenyl-methyl)phosphonate (**9h**). Phosphonoacetamide **7c** (0.20 g, 0.5 mmol), lithium chloride (60 mg, 1.6 mmol), DBU (0.23 g, 0.23 mL, 1.6 mmol) and isobutyraldehyde (40 mg, 0.05 mL, 0.5 mmol) were reacted, obtaining the product **9h** (60% yield, only the diastereoisomer *E*) as a white solid, Mp: 122–124 °C. ^1^H NMR (CDCl_3_, 500 MHz): *δ* 1.00 (d, *J* = 6.7 Hz, 3H, (CH_3_)_2_CH), 1.01 (d, *J* = 6.7 Hz, 3H, (CH_3_)_2_CH), 2.39 (oct, *J* = 6.9 Hz, 1H, CH(CH_3_)_2_), 3.50 (d, *J* = 10.5 Hz, 3H, (CH_3_O)_2_P), 3.79 (s, 3H, CH_3_O), 3.80 (d, *J* = 10.8 Hz, 3H, (CH_3_O)_2_P), 5.65 (dd, *J* = 20.5, 8.7 Hz, 1H, CH-P), 5.87 (dd, *J_trans_* = 15.4, 1.5 Hz, 1H, CH=CH), 6.87 (AA’BB’ system, *J* = 8.3 Hz, 2H, H_arom_), 7.41 (d, *J* = 8.7 Hz, 1H, NH), 7.45 (AA’BB’ system, *J* = 8.9 Hz, 2H, H_arom_), 7.45 (d, *J_trans_* = 15.2 Hz, 1H, CH=CH). ^13^C NMR (CDCl_3_, 125 MHz): *δ* 21.4 ((CH_3_)_2_CH), 21.5 ((CH_3_)_2_CH), 30.9 (CH(CH_3_)_2_), 48.9 (d, *J* = 156.2 Hz, C-P), 53.8 (d, *J* = 7.3 Hz, (CH_3_O)_2_P), 54.0 (d, *J* = 6.8 Hz, (CH_3_O)_2_P), 55.5 (CH_3_O), 114.4 (2C), 120.5 (C=C), 127.3 (2C), 129.8, 152.2 (C=C), 159.7, 165.7 (d, *J* = 7.3 Hz, C=O). ^31^P NMR (CDCl_3_, 202 MHz): *δ* 24.0 ppm. HRMS-CI(+) *m/z*: [M + H]^+^ Calcd for C_16_H_24_NO_5_P + H^+^ 342.1465; Found 342.1483.

Dimethyl *N*-[4,4-dimethylpent-2-ene-1-oxo]-(4-methoxyphenyl-methyl)phosphonate (**9i**). Phosphonoacetamide **7c** (0.20 g, 0.5 mmol), lithium chloride (60 mg, 1.6 mmol), DBU (0.23 g, 0.23 mL, 1.6 mmol) and trimethylacetaldehyde (40 mg, 0.05 mL, 0.5 mmol) were reacted, obtaining the product **9i** (52% yield, only the diastereoisomer *E*) as a white solid, Mp: 148–150 °C. ^1^H NMR (CDCl_3_, 500 MHz): *δ* 1.03 (s, 9H, ((CH_3_)_3_C), 3.50 (d, *J* = 10.5 Hz, 3H, (CH_3_O)_2_P), 3.79 (s, 3H, CH_3_O), 3.80 (d, *J* = 10.8 Hz, 3H, (CH_3_O)_2_P), 5.65 (dd, *J* = 20.4, 9.6 Hz, 1H, CH-P), 5.82 (d, *J_trans_* = 15.6 Hz, 1H, CH=CH), 6.84 (AA’BB’ system, *J* = 8.5 Hz, 2H, H_arom_), 7.32 (d, *J* = 9.6 Hz, 1H, NH), 7.45 (d, *J_trans_* = 15.4, 1H, CH=CH), 7.45 (AA’BB’ system, *J* = 8.5 Hz, 2H, H_arom_). ^13^C NMR (CDCl_3_, 125 MHz): *δ* 28.9 ((CH_3_)_3_C), 33.7 (C(CH_3_)_3_), 48.9 (d, *J* = 156.7 Hz, C-P), 53.8 (d, *J* = 7.3 Hz, (CH_3_O)_2_P), 54.0 (d, *J* = 6.8 Hz, (CH_3_O)_2_P), 55.5 (CH_3_O), 114.4 (2C), 118.5 (C=C), 127.3, 129.8 (2C), 156.0 (C=C), 159.7, 165.9 (d, *J* = 7.7 Hz, C=O). ^31^P NMR (CDCl_3_, 202 MHz): δ 24.3 ppm. HRMS-CI(+) *m/z*: [M + H]^+^ Calcd for C_17_H_26_NO_5_P + H^+^ 356.1621; Found 356.1638.

## 4. Conclusions

In conclusion, the easy access to α-aminophosphonates **5a**–**c** through the Kabachnik–Fields reaction and phosphonoacetamides **7a**–**c** in combination with high selectivity *E*/*Z* and good chemical yields in the Horner–Wadsworth–Emmons reaction, thus as the optimization of the HWE reaction using DBU as a base in the presence of LiCl, make this experimental procedure an excellent, practical and general methodology to obtain (*E*)-α,β-unsaturated amides bearing an α-aminophosphonate moiety. The biological evaluation of the obtained (*E*)-α,β-unsaturated amides as potential anticancer agents is currently underway.

Optimization of the HWE reaction was achieved using DBU as a base in the presence of LiCl.

## Figures and Tables

**Table 1:** Optimization of reaction conditions for the preparation of α,β-unsaturated amide **3a**.

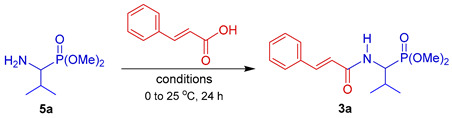		
Entry	Conditions	Yield (%)
1	SOCl_2_, DMF, DIPEA, CH_2_Cl_2_	56
2	HBTU, DIPEA, CH_2_Cl_2_	76
3	PyBOP, DIPEA, MeCN	77
4	DCC, DMAP, CH_2_Cl_2_	74
5	*i*-BuOC(O)Cl, NMM, CH_2_Cl_2_	72
6	(Boc)_2_O, Py, DMAP, THF	68

**Table 2:** Optimization of HWE reaction conditions for the preparation of α,β-unsaturated amide **3a**.

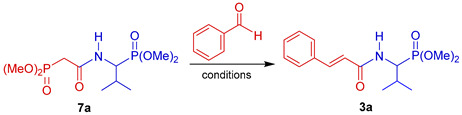				
Entry	Conditions	Time (h)	Yield (%) ^1^	E:Z ^2^
1	Cs_2_CO_3_, MeCN, 50 °C	12	72	93:07
2	Cs_2_CO_3_, MeCN, 80 °C	6	70	92:08
3	Cs_2_CO_3_, PhMe, 100 °C	4	65	93:07
4	K_2_CO_3_, MeCN, 50 °C	24	60	91:09
5	K_2_CO_3_, MeCN, 80 °C	20	63	87:13
6	K_2_CO_3_, PhMe, 100 °C	16	68	61:39
7	DBU, LiCl, THF, 25 °C	3	90	94:06

^1^ Isolated yields after purification by column chromatography. ^2^ The *E:Z* ratio was determined by ^1^H NMR in the crude product.

## Data Availability

Data contained within the article or Supplementary Materials.

## Supplementary Materials

The following supporting information can be downloaded at: https://www.mdpi.com/article/10.3390/molecules30183730/s1, Figures S1–S105: Spectroscopic data.

## References

[1] Sova M. (2012). Antioxidant and Antimicrobial Activities of Cinnamic Acid Derivatives. Mini-Rev. Med. Chem..

[2] De P., Baltas M., Bedos-Belval F. (2011). Cinnamic Acid Derivatives as Anticancer Agents-A Review. Curr. Med. Chem..

[3] Silva A.T., Bento C.M., Pena A.C., Figueiredo L.M., Prudêncio C., Aguiar L., Silva T., Ferraz R., Gomes M.S., Teixeira C. (2020). Cinnamic Acid Conjugates in the Rescuing and Repurposing of Classical Antimalarial Drugs. Molecules.

[4] Gaikwad N., Nanduri S., Madhavi Y.V. (2019). Cinnamamide: An insight into the pharmacological advances and structure–activity relationships. Eur. J. Med. Chem..

[5] Çalışkan E., Öztürk D.A., Koran K., Tekin S., Sandal S., Erkan S., Görgülü A.O., Çetina A. (2022). Synthesis of New Cinnamoyl-Amino Acid Conjugates and in Vitro Cytotoxicity and Genotoxicity Studies. Chem. Biodivers..

[6] Pérez B.C., Teixeira C., Figueiras M., Gut J., Rosenthal F.J., Gomes J.R.B., Gomes P. (2012). Novel cinnamic acid/4-aminoquinoline conjugates bearing non-proteinogenic amino acids: Towards the development of potential dual action antimalarials. Eur. J. Med. Chem..

[7] Fan Q., Jiang H., Yuan E.-d., Zhang J.-x., Ning Z.-x., Qi S.-j., Wei Q.-y. (2012). Tyrosinase inhibitory effects and antioxidative activities of novel cinnamoyl amides with amino acid ester moiety. Food Chem..

[8] Spasova M., Kortenska-Kancheva V., Totseva I., Ivanova G., Georgiev L., Milkova T. (2006). Synthesis of cinnamoyl and hydroxycinnamoyl amino acid conjugates and evaluation of their antioxidant activity. J. Peptide Sci..

[9] Huang S., Liu W., Li Y., Zhang K., Zheng X., Wu H., Tang T. (2021). Design, Synthesis, and Activity Study of Cinnamic Acid Derivatives as Potent Antineuroinflammatory Agents. ACS Chem. Neurosci..

[10] Mucha A., Kafarski P., Berlicki L. (2011). Remarkable Potential of the α-Aminophosphonate/Phosphinate Structural Motif in Medicinal Chemistry. J. Med. Chem..

[11] Orsini F., Sello G., Sisti M. (2010). Aminophosphonic Acids and Derivatives. Synthesis and Biological Applications. Curr. Med. Chem..

[12] Naydenova E.D., Todorov P.T., Troev K.D. (2010). Recent synthesis of aminophosphonic acids as potential biological importance. Amino Acids.

[13] Gluza K., Kafarski P., El-Shemy H. (2013). Drug Discovery.

[14] Lejczak B., Kafarski P. (2009). Biological Activity of Aminophosphonic Acids and Their Short Peptides. Top. Heterocyclic Chem..

[15] Ordoñez M., Viveros-Ceballos J.L., Romero-Estudillo I., Asao T., Asaduzzaman M.D. (2017). Chap. 6 stereoselective synthesis of α-aminophosphonic acids through Pudovik and Kabachnik Fields-reaction. Amino Acid-New Insights and Roles in Plant and Animal.

[16] Mayorquín-Torres M.C., Simoens A., Bonneure E., Stevens C.V. (2024). Synthetic Methods for Azaheterocyclic Phosphonates and Their Biological Activity: An Update 2004–2024. Chem. Rev..

[17] Amira A., Aouf Z., K’tir H., Chemam Y., Ghodbane R., Zerrouki R., Aouf N.-E. (2021). Recent Advances in the Synthesis of α-Aminophosphonates: A Review. ChemistrySelect.

[18] Varga P.R., Keglevich G. (2021). Synthesis of α-Aminophosphonates and Related Derivatives; The Last Decade of the Kabachnik–Fields Reaction. Molecules.

[19] Kudzin Z.H., Kudzin M.H., Drabowicz J., Stevens C.V. (2011). Aminophosphonic acids-phosphorus analogues of natural amino acids. Part 1: Syntheses of α-aminophosphonic acids. Curr. Org. Chem..

[20] Ali T.E., Abdel-Kariem S.M. (2015). Methods for the synthesis of α-heterocyclic/heteroarylα-aminophosphonic acids and their esters. Arkivoc.

[21] Golovchenko A.V., Pilyo S.G., Brovarets V.S., Chernega A.N., Dracha B.S. (2003). A Facile Synthesis of Derivatives of (1,3,4-Thiadiazol-2-yl)glycine and Its Phosphonyl Analogue. Synthesis.

[22] Hu D., Wan Q.-Q., Yang S., Song B., Bhadury P.S., Jin L.-H., Yan K., Liu F., Chen Z., Xue W. (2008). Synthesis and antiviral activities of amide derivatives containing the α-aminophosphonate moiety. J. Agric. Food Chem..

[23] Lan X., Xie D., Yin L., Wang Z., Chen J., Zhang A., Song B., Hu D. (2017). Novel *α,β*-unsaturated amide derivatives bearing *α*-aminophosphonate moiety as potential antiviral agents. Bioorg. Med. Chem. Lett..

[24] Jackson D.S., Fraser S.A., Ni L.-M., Kam C.-M., Winkler U., Johnson D.A., Froelich C.J., Hudig D., Powers J.C. (1998). Synthesis and Evaluation of Diphenyl Phosphonate Esters as Inhibitors of the Trypsin-like Granzymes A and K and Mast Cell Tryptase. J. Med. Chem..

[25] Moreno-Cinos C., Sassetti E., Salado I.G., Witt G., Benramdane S., Reinhardt L., Cruz C.D., Joossens J., Van der Veken P., Brötz-Oesterhelt H. (2019). α-Amino Diphenyl Phosphonates as Novel Inhibitors of Escherichia coli ClpP Protease. J. Med. Chem..

[26] Akgun B., Savci E., Avci D. (2012). Synthesis and Polymerizations of Phosphonated-bis(methacrylamide)s for Dental Applications. J. Polym. Sci. Part A.

[27] Anandhan R., Prasanth K., Nithishkumar P. (2024). Purple light-induced Ritter-type reaction of diazophosphonates: Access to α-amido-β-keto phosphonates. Org. Biomol. Chem..

[28] Morales-Solís J.C., Ordoñez M., Viveros-Ceballos J.L. (2024). Stereodivergent Synthesis of the Four Stereoisomers of Diethyl 4-Hydroxyphosphopipecolate from Ethyl (*R*)-4-Cyano-3-hydroxybutanoate. Synthesis.

[29] Kabachnik M.I., Medved T. (1952). Нoвый метoд синтеза сс-аминoфoсфинoвых кислoт». Dokl. Akad. Nauk SSSR.

[30] Fields E.K. (1952). The synthesis of esters of substituted amino phosphonic acids. J. Am. Chem. Soc..

[31] Bedolla-Medrano M., Hernández-Fernández E., Ordoñez M. (2014). Phenylphosphonic acid as efficient and recyclable catalyst in the synthesis of α-aminophosphonates under solvent-free conditions. Synlett.

[32] Hernández-Fernández E., Fernandez-Zertuche M., García-Barradas O., Muñoz-Muñiz O., Ordoñez M. (2006). Practical and Efficient Synthesis of (*E*)-α,β-Unsaturated Amides Bearing (*S*)-α-Methylbenzylamine from 2-Phosphonamides via Horner-Wadsworth-Emmons Reaction. Synlett.

[33] Ordoñez M., Hernández-Fernández E., Montiel-Pérez M., Rojas-Cabrera H., Fernandez-Zertuche M., García-Barradas O. (2007). A convenient Method for the Preparation of Chiral Phosphonoacetamides and their Horner-Wadsworth-Emmons Reaction. Tetrahedron Asymmetry.

[34] Blanchette M.A., Choy W., Davis J.T., Essenfeld A.P., Masamune S., Roush W.R., Sakai T. (1984). Horner-wadsworth-emmons reaction: Use of lithium chloride and an amine for base-sensitive compounds. Tetrahedron Lett..

[35] Bhagat S., Chakraborti A.K. (2007). An Extremely Efficient Three-Component Reaction of Aldehydes/Ketones, Amines, and Phosphites (Kabachnik-Fields Reaction) for the Synthesis of α-Aminophosphonates Catalyzed by Magnesium Perchlorate. J. Org. Chem..

[36] Zielinska K., Mazurkiewicz R., Szymanska K., Jarzebski A., Magiera S., Erfurt K. (2016). Penicillin G acylase-mediated kinetic resolution of racemic 1-(*N-*acylamino)alkylphosphonic and 1-(*N*-acylamino)alkylphosphinic acids and their esters. J. Mol. Catal. B Enzym..

[37] Azizi N., Rajabi F., Saidi M.R. (2004). A mild and highly efficient protocol for the one-pot synthesis of primary α-amino phosphonates under solvent-free conditions. Tetrahedron Lett..

