# Supplementary material for: Practical and Efficient Synthesis of (E)-α,β-Unsaturated Amides Incorporating α-Aminophosphonates via the Horner–Wadsworth–Emmons Reaction
Source: Molecules. 2025 Sep 13;30(18):3730. doi: 10.3390/molecules30183730 (PMC12472741; doi:10.3390/molecules30183730)
Supplement: Supplementary file 1 [file molecules-30-03730-s001.zip › molecules-3849742-supplementary.pdf]

Supplementary Information for

Practical and Efficient Synthesis of (*E*)-α,β-Unsaturated Amides Incorporating α-Aminophosphonates via the Horner–Wadsworth–Emmons Reaction

Sindy Anahi Pérez-Aniceto[a], Erica Cano-Tapia[a], Mario Ordoñez*[a], José Luis Viveros-Ceballos*[a] and Ivan Romero-Estudillo[b]

[a] Centro de Investigaciones Químicas-IICBA, Universidad Autónoma del Estado de Morelos, Av. Universidad 1001, 62209 Cuernavaca, Morelos, México; [b] Secihti-Centro de Investigaciones Químicas-IICBA, Universidad Autónoma del Estado de Morelos, Av. Universidad 1001, 62209 Cuernavaca, Morelos, México.

* Correspondence: palacios@uaem.mx (MO) jlvc@uaem.mx (JLVC);

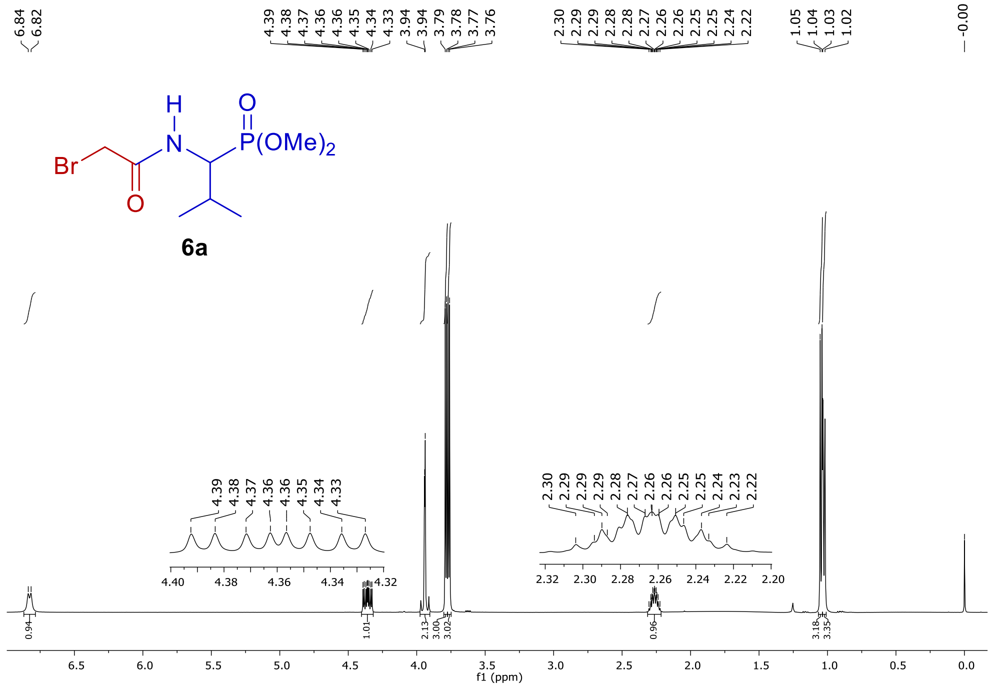

Figure S1. Spectrum of dimethyl (1-(2-bromoacetamido)-2-methylpropyl)phosphonate **6a** (^{1}H NMR 500 MHz, $CDCl_3$).

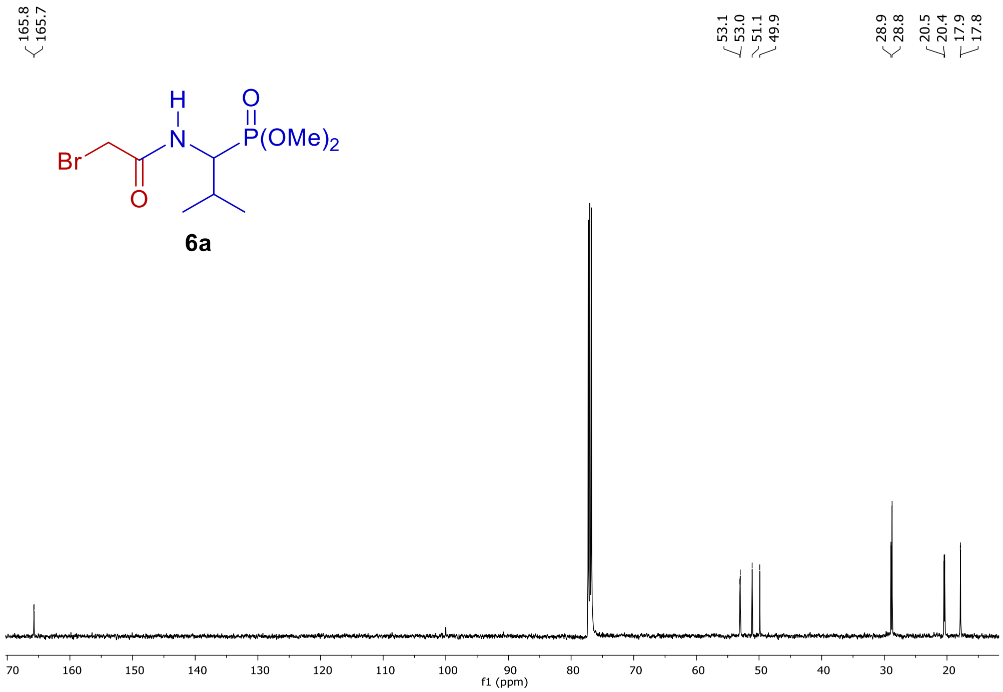

Figure S2. Spectrum of dimethyl (1-(2-bromoacetamido)-2-methylpropyl)phosphonate **6a** (^{13}C NMR 125 MHz, $CDCl_3$).

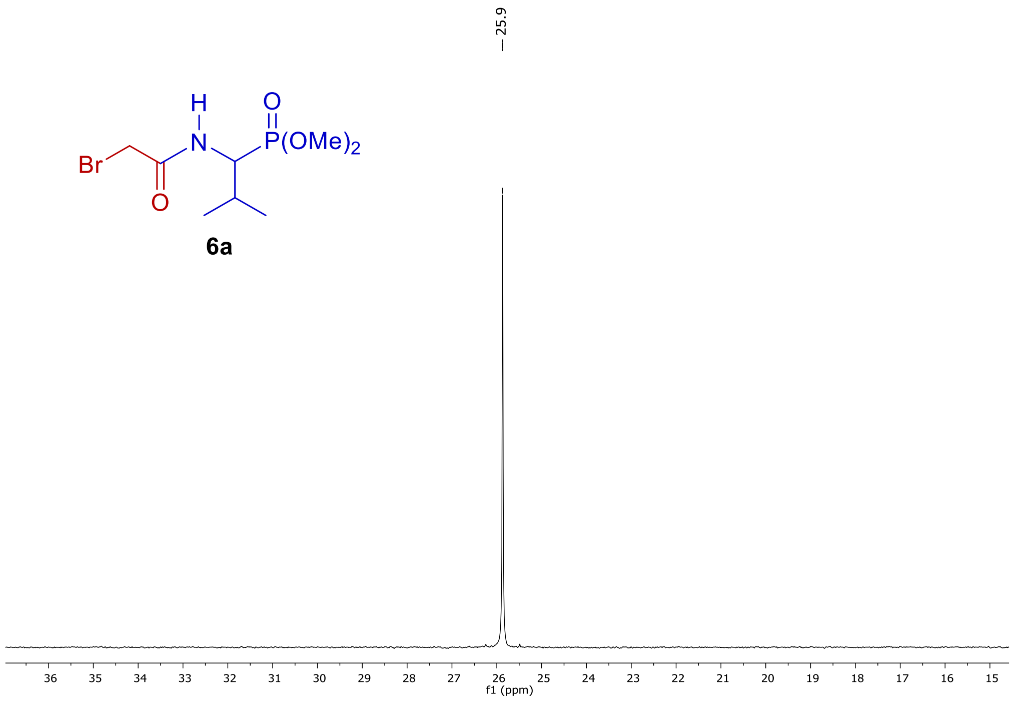

Figure S3. Spectrum of dimethyl (1-(2-bromoacetamido)-2-methylpropyl)phosphonate **6a** (^{31}P NMR 202 MHz, $CDCl_3$).

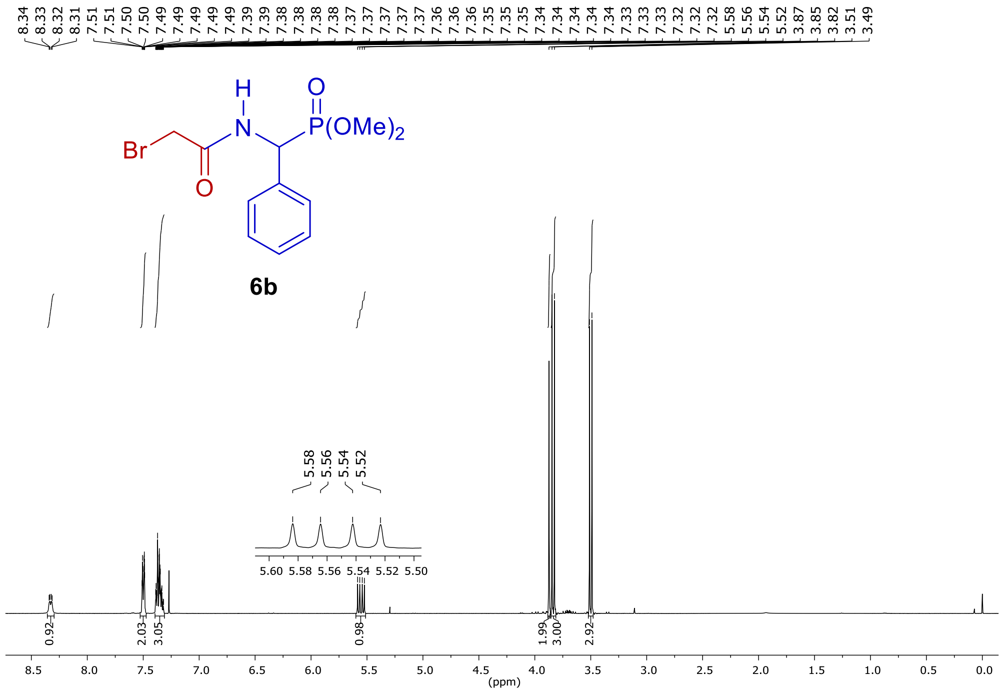

Figure S4. Spectrum of dimethyl ((2-bromoacetamido)(phenyl)methyl)phosphonate **6b** (^{1}H NMR 500 MHz, $CDCl_3$).

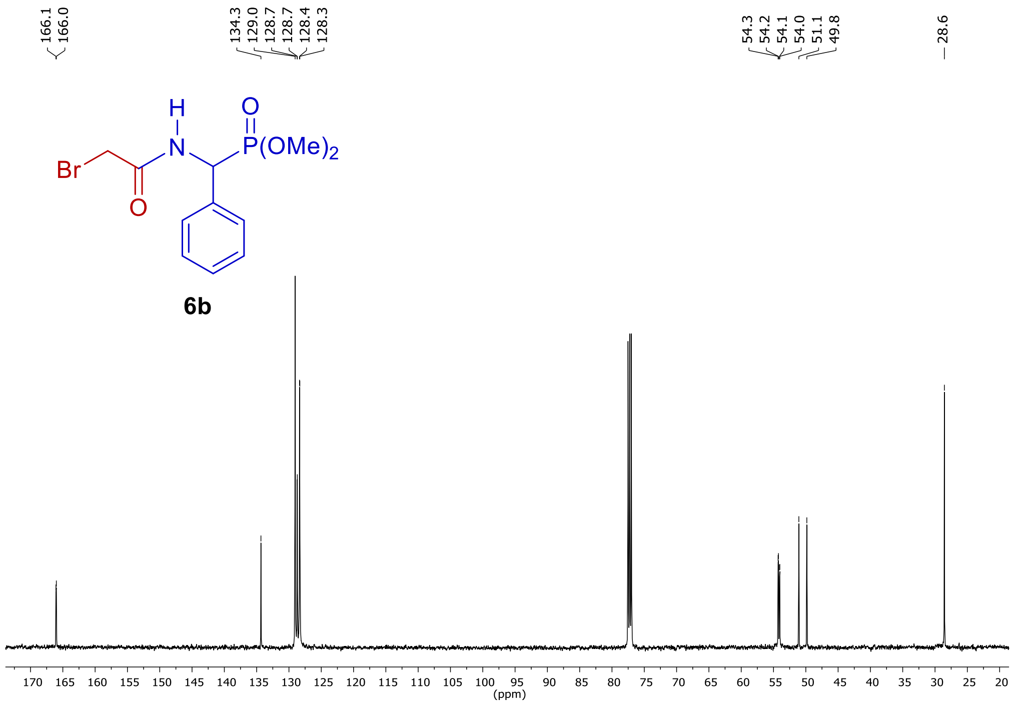

Figure S5. Spectrum of dimethyl ((2-bromoacetamido)(phenyl)methyl)phosphonate **6b** (^{13}C NMR 125 MHz, CDCl$_3$).

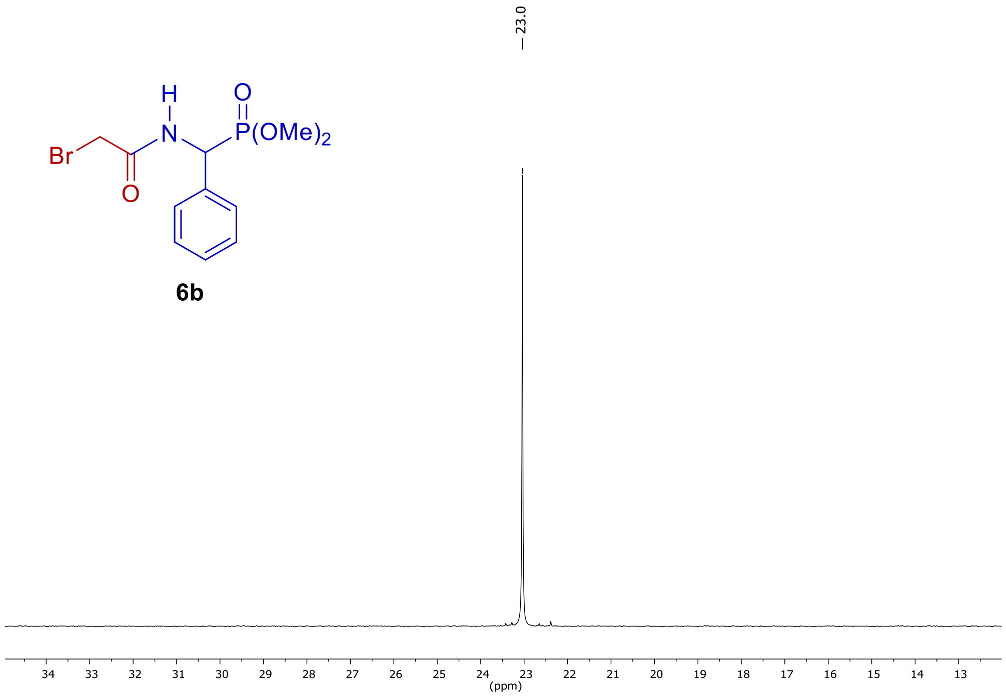

Figure S6. Spectrum of dimethyl ((2-bromoacetamido)(phenyl)methyl)phosphonate **6b** (^{31}P NMR 202 MHz, CDCl$_3$).

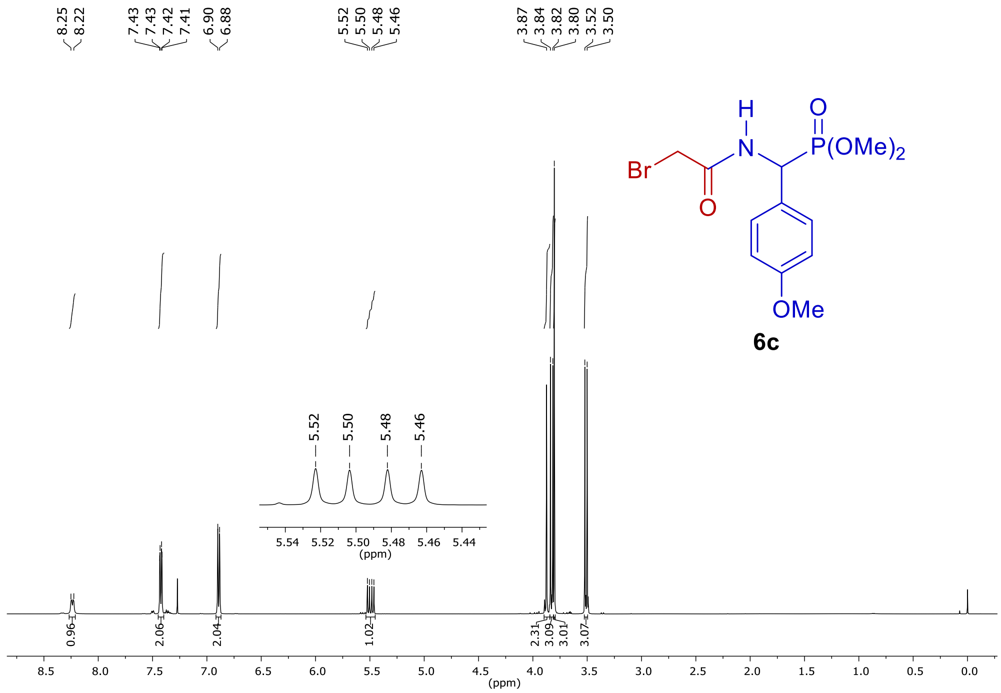

Figure S7. Spectrum of dimethyl ((2-bromoacetamido)(4-methoxyphenyl)methyl)phosphonate **6c** (^{1}H NMR 500 MHz, $CDCl_3$).

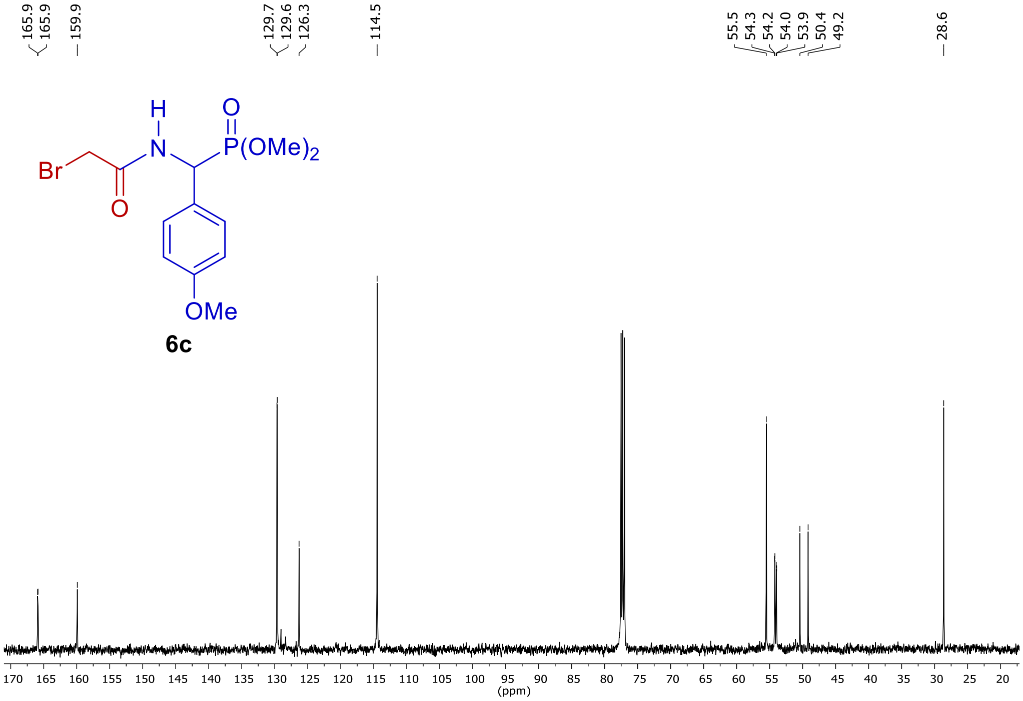

Figure S8. Spectrum of dimethyl ((2-bromoacetamido)(4-methoxyphenyl)methyl)phosphonate **6c** (^{13}C NMR 125 MHz, $CDCl_3$).

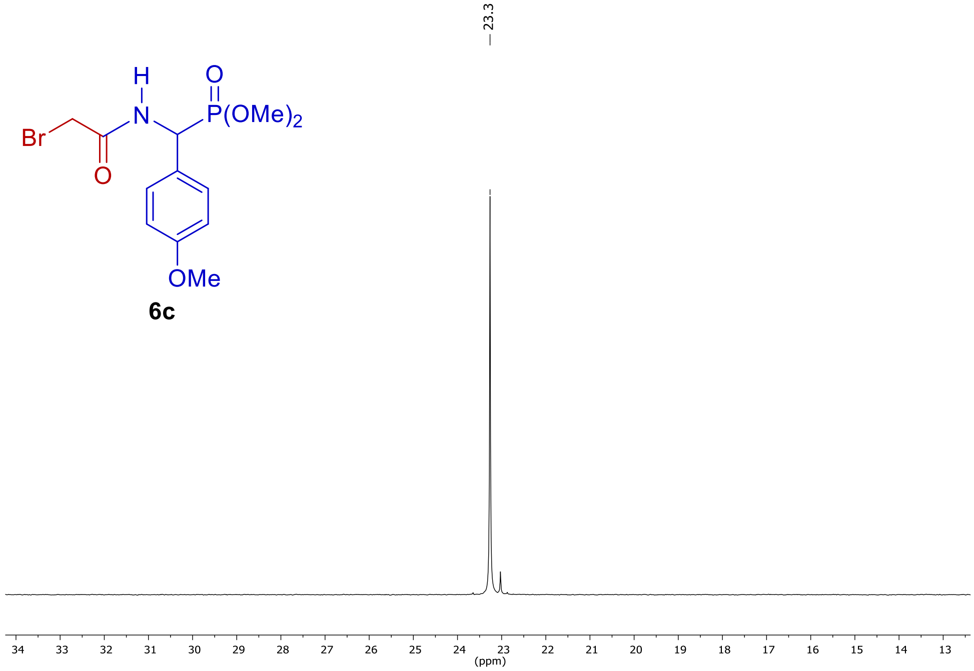

Figure S9. Spectrum of dimethyl ((2-bromoacetamido)(4-methoxyphenyl)methyl)phosphonate **6c** (^{31}P NMR 202 MHz, $CDCl_3$).

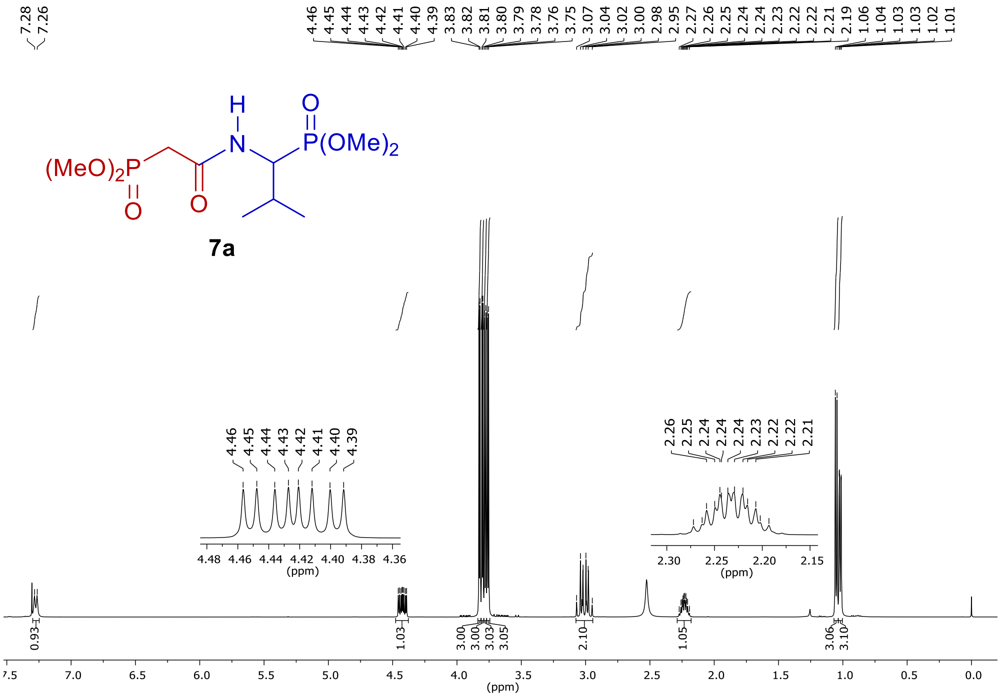

Figure S10. Spectrum of dimethyl (2-((1-(dimethoxyphosphoryl)-2-methylpropyl)amino)-2-oxoethyl)phosphonate **7a** (^{1}H NMR 500 MHz, $CDCl_3$).

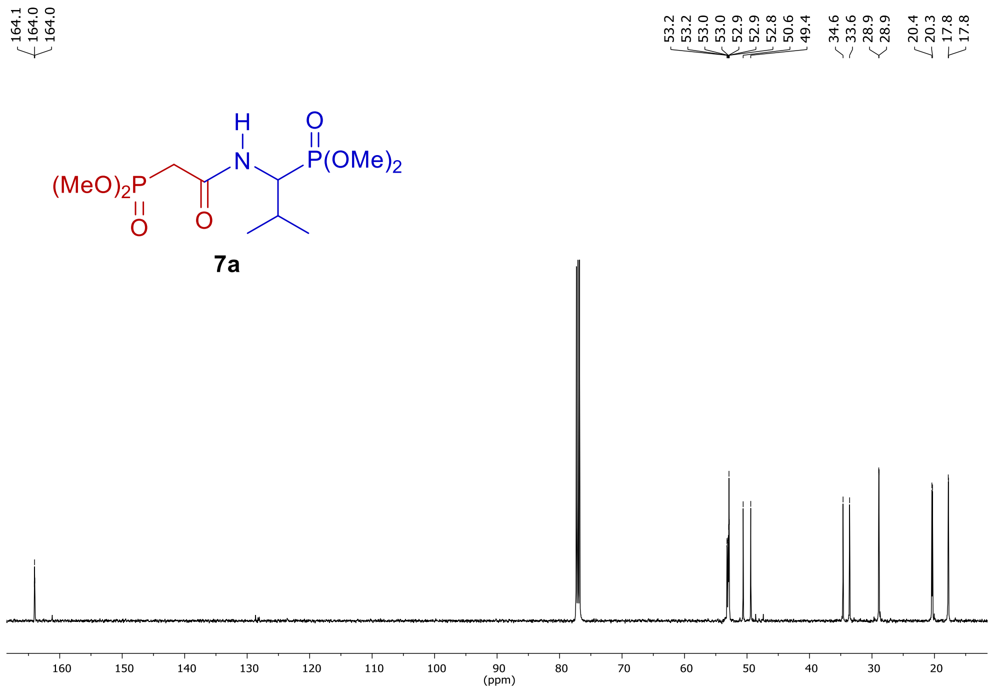

Figure S11. Spectrum of dimethyl (2-((1-(dimethoxyphosphoryl)-2-methylpropyl)amino)-2-oxoethyl)phosphonate **7a** (^{13}C NMR 125 MHz, $CDCl_3$).

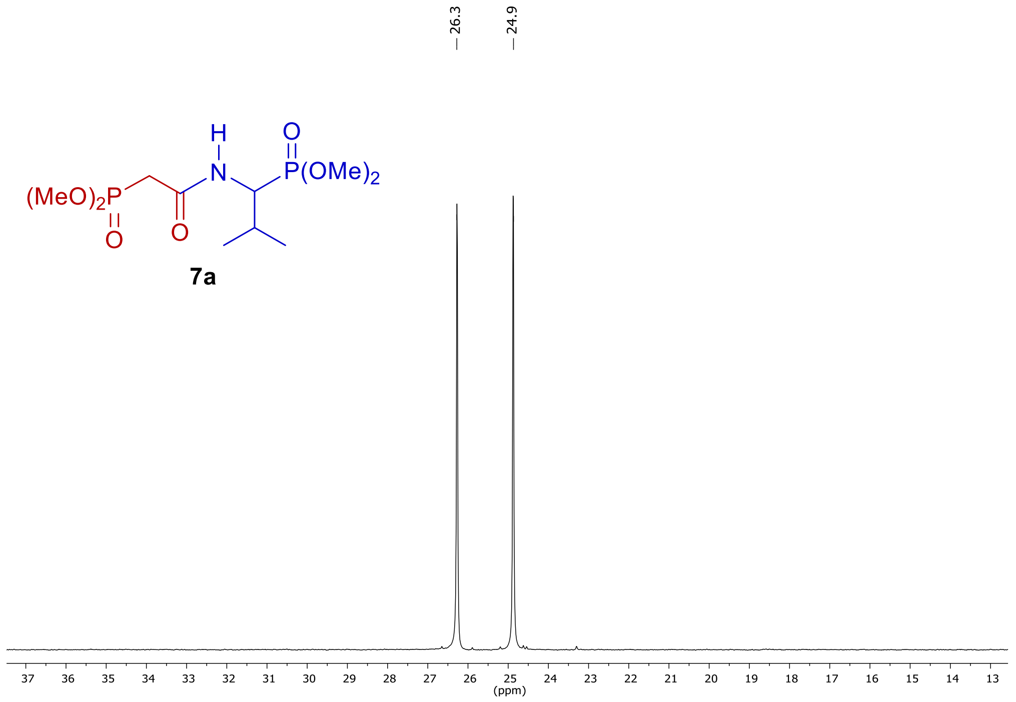

Figure S12. Spectrum of dimethyl (2-((1-(dimethoxyphosphoryl)-2-methylpropyl)amino)-2-oxoethyl)phosphonate **7a** (^{31}P NMR 202 MHz, $CDCl_3$).

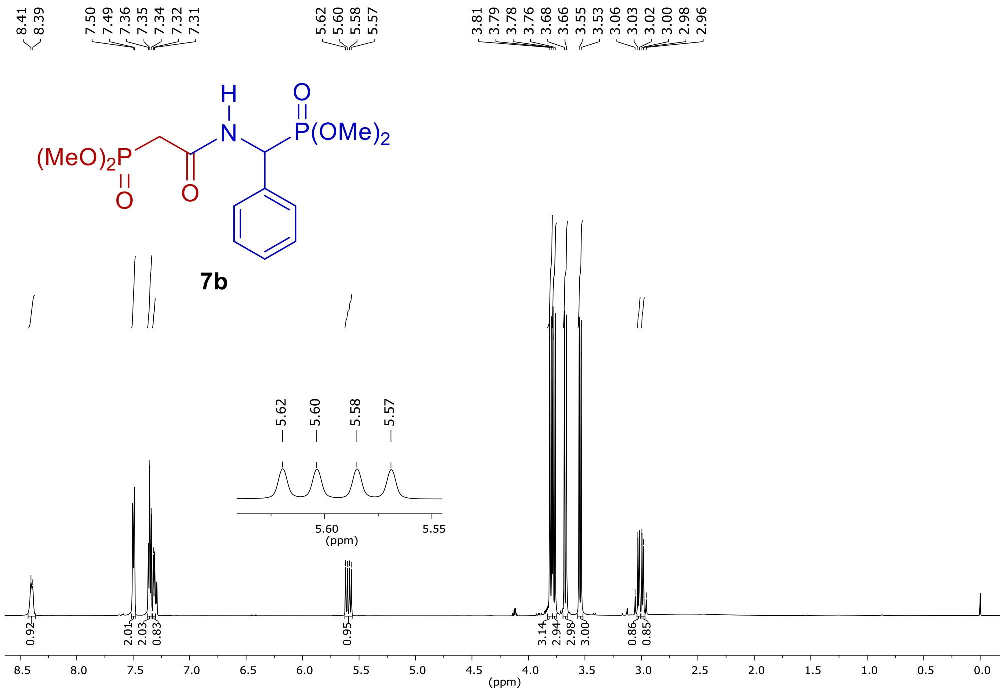

Figure S13. Spectrum of dimethyl (2-(((dimethoxyphosphoryl)(phenyl)methyl)amino)-2-oxoethyl)phosphonate **7b** (^{1}H NMR 600 MHz, $CDCl_3$).

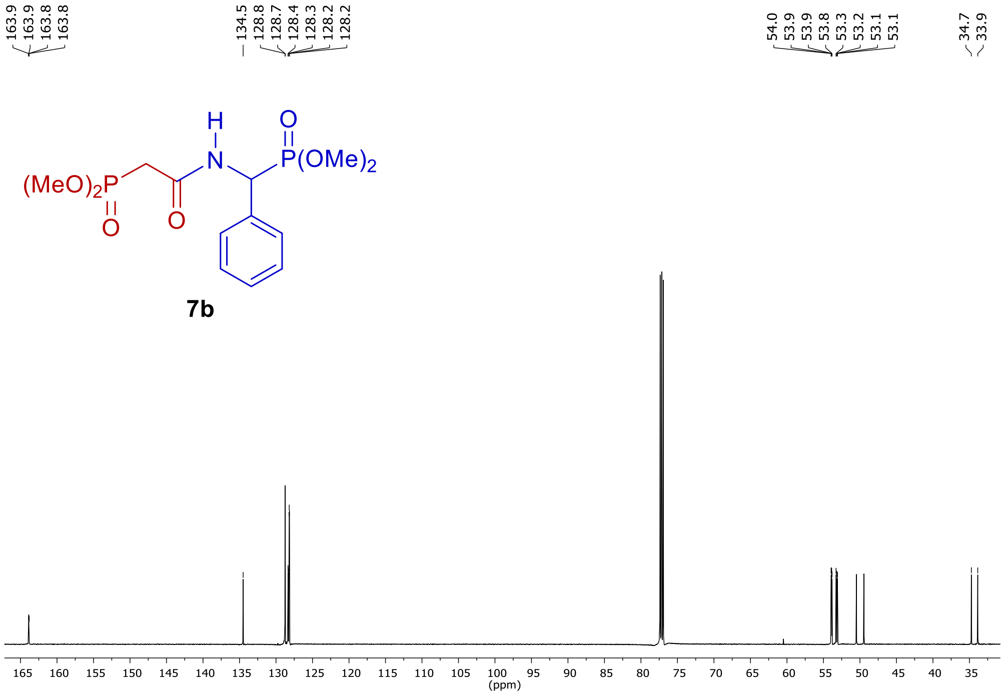

Figure S14. Spectrum of dimethyl (2-(((dimethoxyphosphoryl)(phenyl)methyl)amino)-2-oxoethyl)phosphonate **7b** (^{13}C NMR 151 MHz, $CDCl_3$).

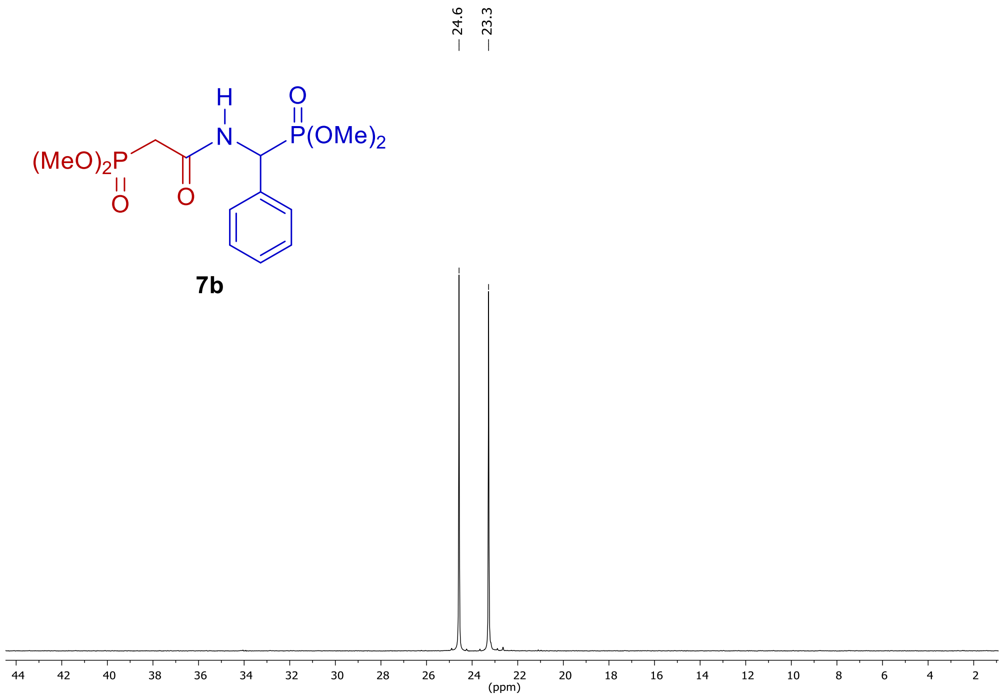

Figure S15. Spectrum of dimethyl (2-(((dimethoxyphosphoryl)(phenyl)methyl)amino)-2-oxoethyl)phosphonate **7b** (^{31}P NMR 202 MHz, $CDCl_3$).

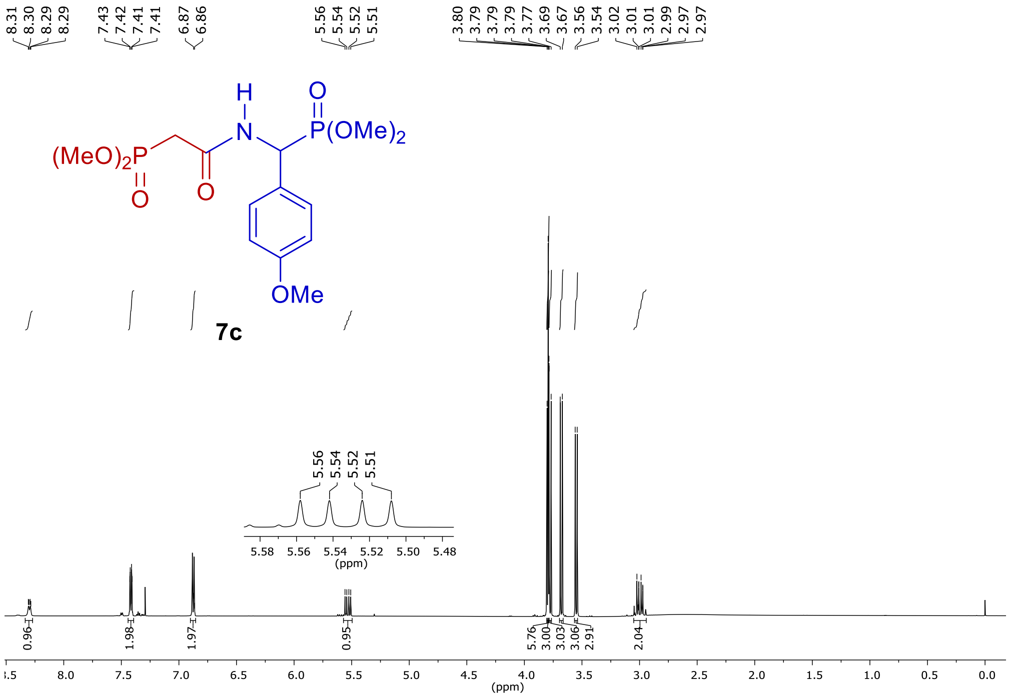

Figure S16. Spectrum of dimethyl (2-(((dimethoxyphosphoryl)(4-methoxyphenyl)methyl)amino)-2-oxoethyl)phosphonate **7c** (^{1}H NMR 600 MHz, $CDCl_3$).

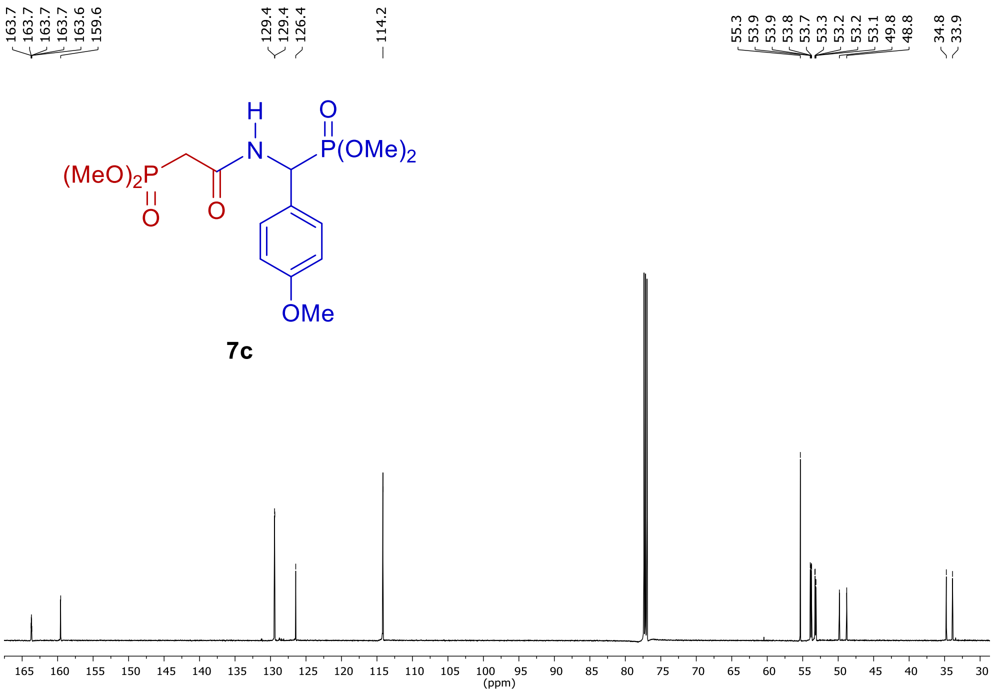

Figure S17. Spectrum of dimethyl (2-(((dimethoxyphosphoryl)(4-methoxyphenyl)methyl)amino)-2-oxoethyl)phosphonate **7c** (^{13}C NMR 151 MHz, $CDCl_3$).

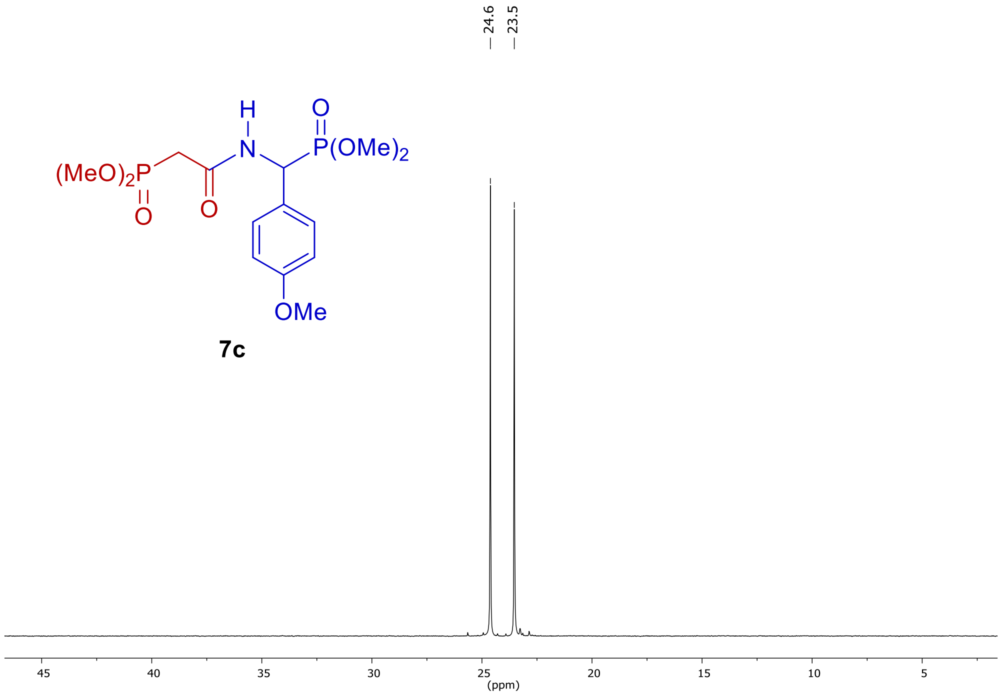

Figure S18. Spectrum of dimethyl (2-(((dimethoxyphosphoryl)(4-methoxyphenyl)methyl)amino)-2-oxoethyl)phosphonate **7c** (^{31}P NMR 202 MHz, $CDCl_3$).

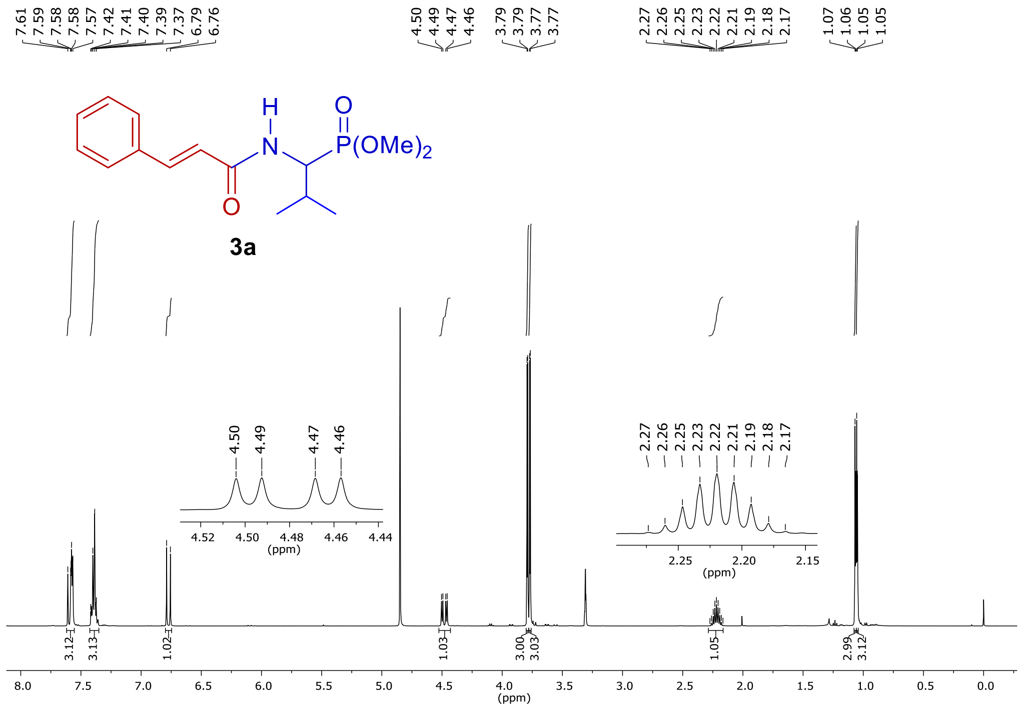

Figure S19. Spectrum of dimethyl *N*-[3-phenyl-2-ene-1-oxo]-2-(methylpropyl)phosphonate **3a** (^{1}H NMR 500 MHz, CD_3OD).

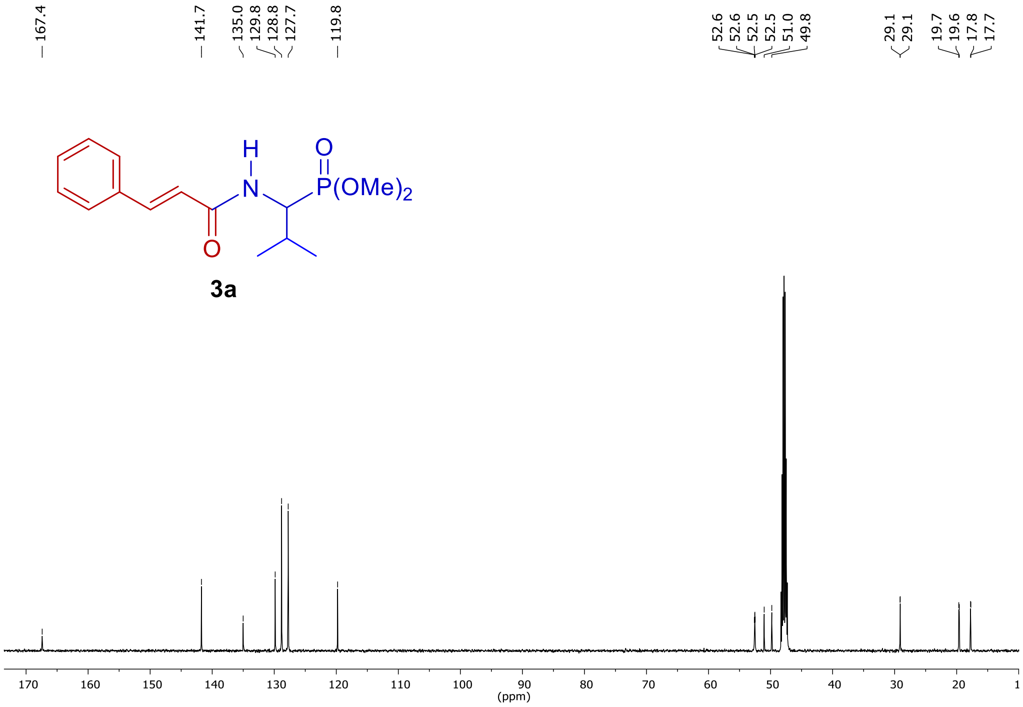

Figure S20. Spectrum of dimethyl *N*-[3-phenyl-2-ene-1-oxo]-2-(methylpropyl)phosphonate **3a** (^{13}C NMR 125 MHz, CD_3OD).

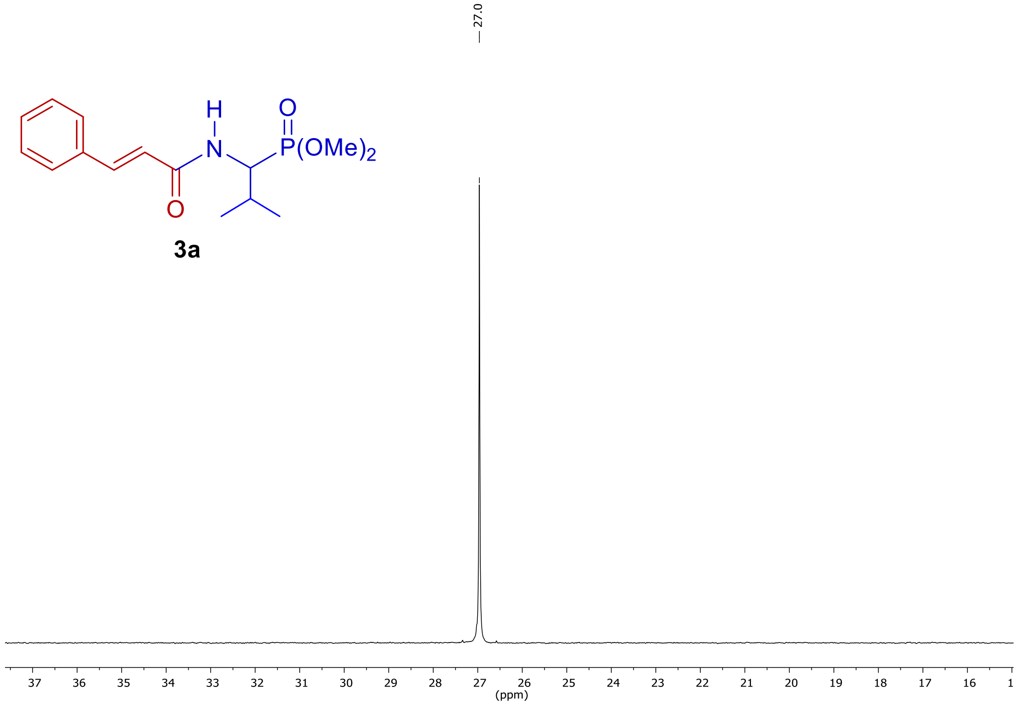

Figure S21. Spectrum of dimethyl *N*-[3-phenyl-2-ene-1-oxo]-2-(methylpropyl)phosphonate **3a** (^{31}P NMR 202 MHz, CD_3OD).

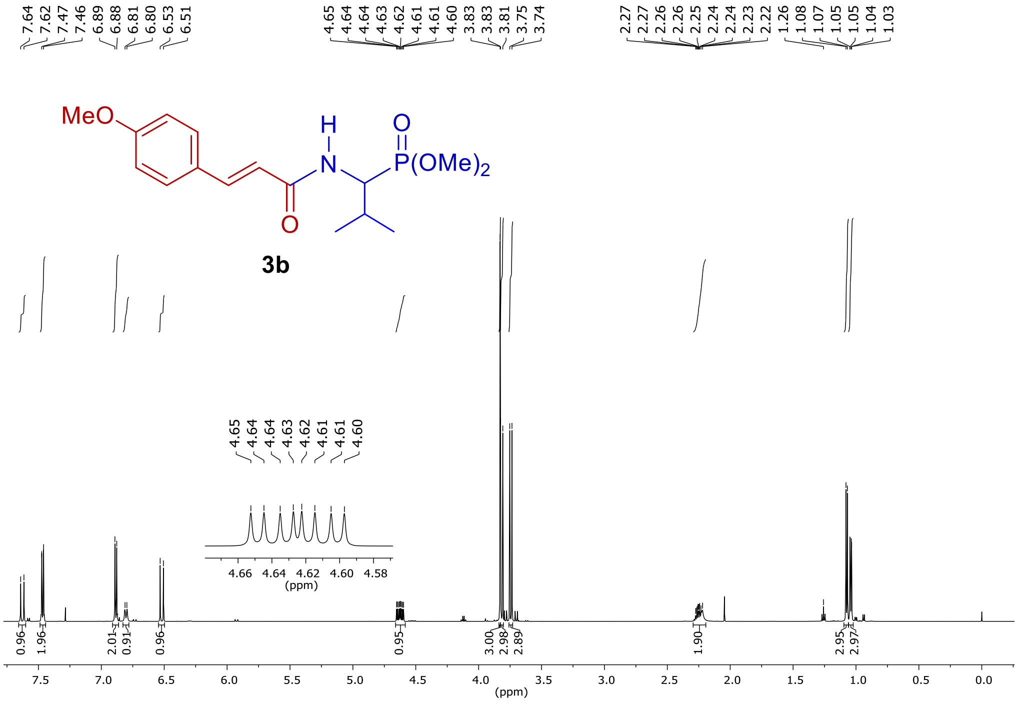

Figure S22. Spectrum of dimethyl *N*-[3-(4-methoxyphenyl)-2-ene-1-oxo]-2-(methylpropyl)phosphonate **3b** (^{1}H NMR 600 MHz, $CDCl_3$).

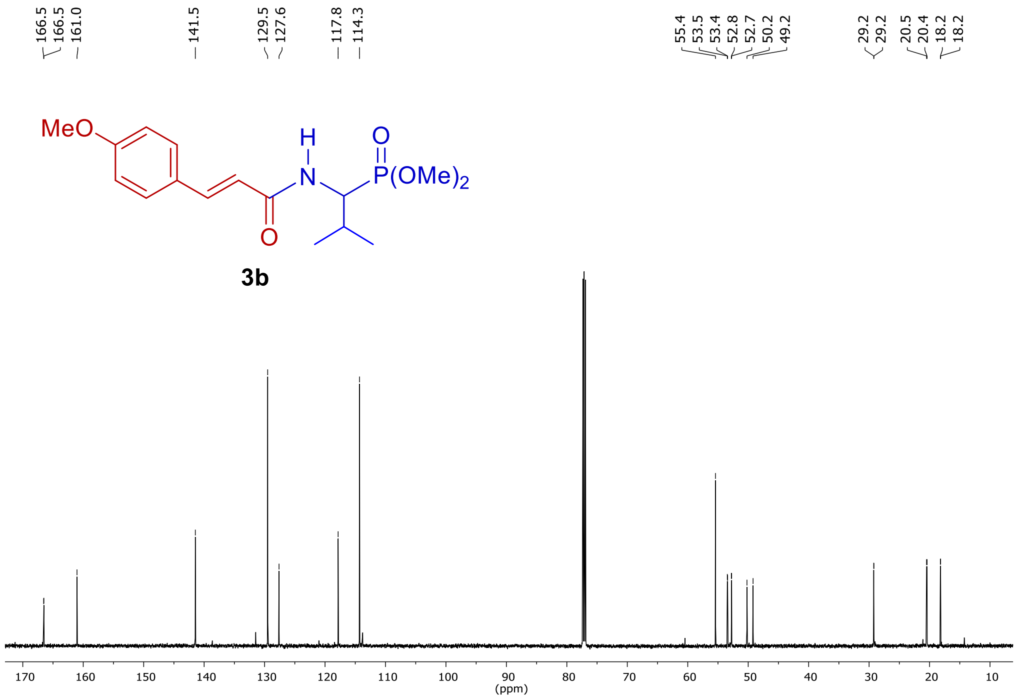

Figure S23. Spectrum of dimethyl *N*-[3-(4-methoxyphenyl)-2-ene-1-oxo]-2-(methylpropyl)phosphonate **3b** (^{13}C NMR 151 MHz, $CDCl_3$).

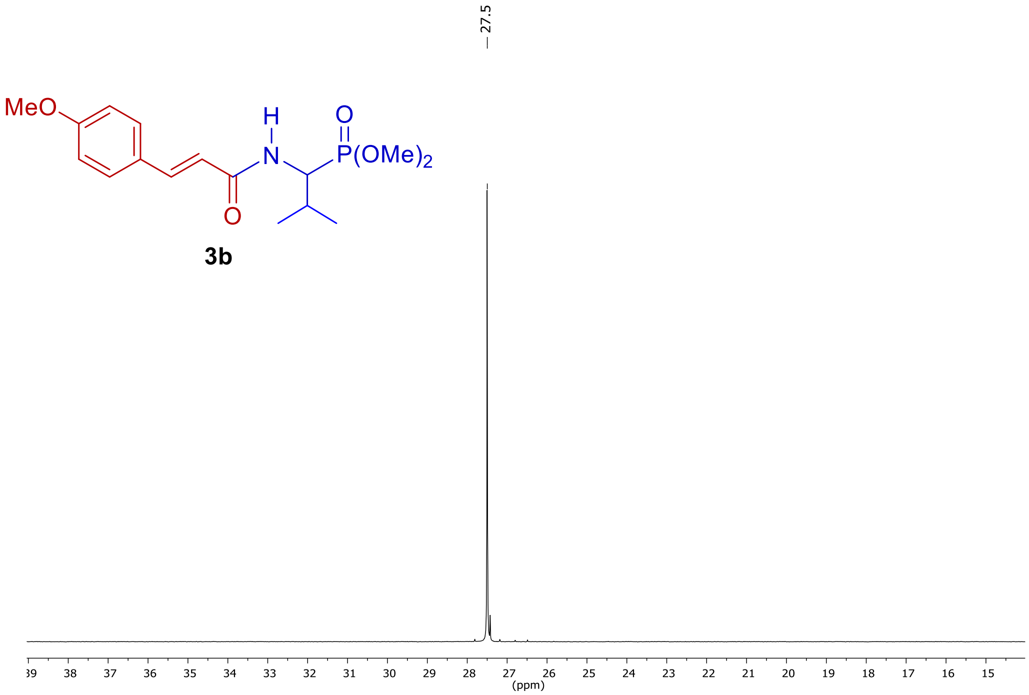

Figure S24. Spectrum of dimethyl *N*-[3-(4-methoxyphenyl)-2-ene-1-oxo]-2-(methylpropyl)phosphonate **3b** (^{31}P NMR 243 MHz, $CDCl_3$).

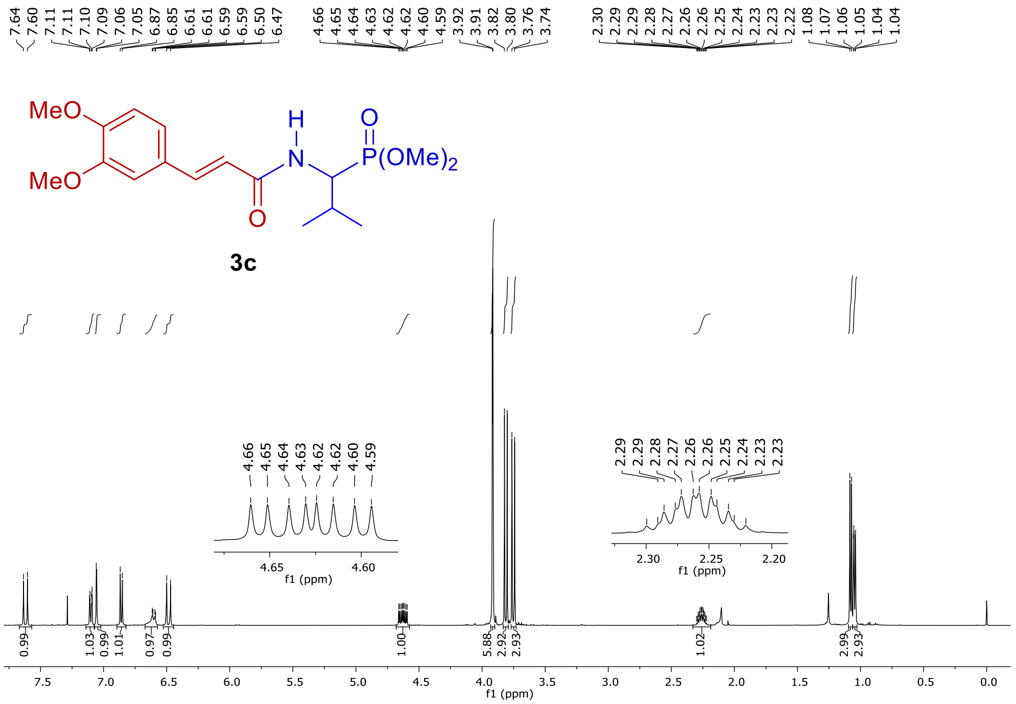

Figure S25. Spectrum of dimethyl *N*-[3-(4,3-dimethoxyphenyl)-2-ene-1-oxo]-2-(methylpropyl)phosphonate **3c** (^{1}H NMR 500 MHz, $CDCl_3$).

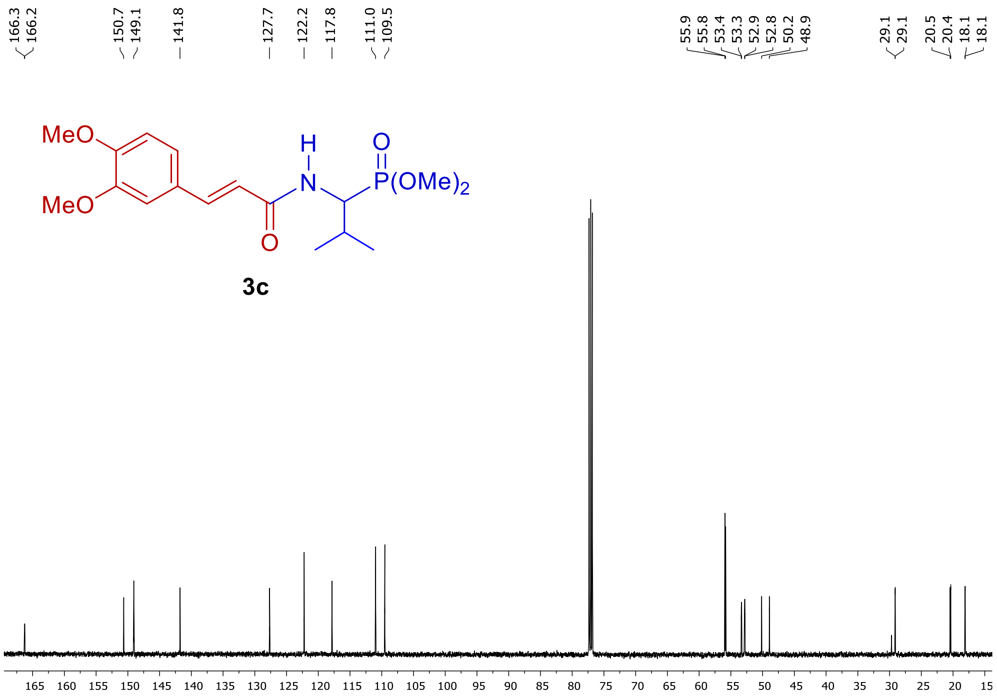

Figure S26. Spectrum of dimethyl *N*-[3-(4,3-dimethoxyphenyl)-2-ene-1-oxo]-2-(methylpropyl)phosphonate **3c** (^{13}C NMR 125 MHz, $CDCl_3$).

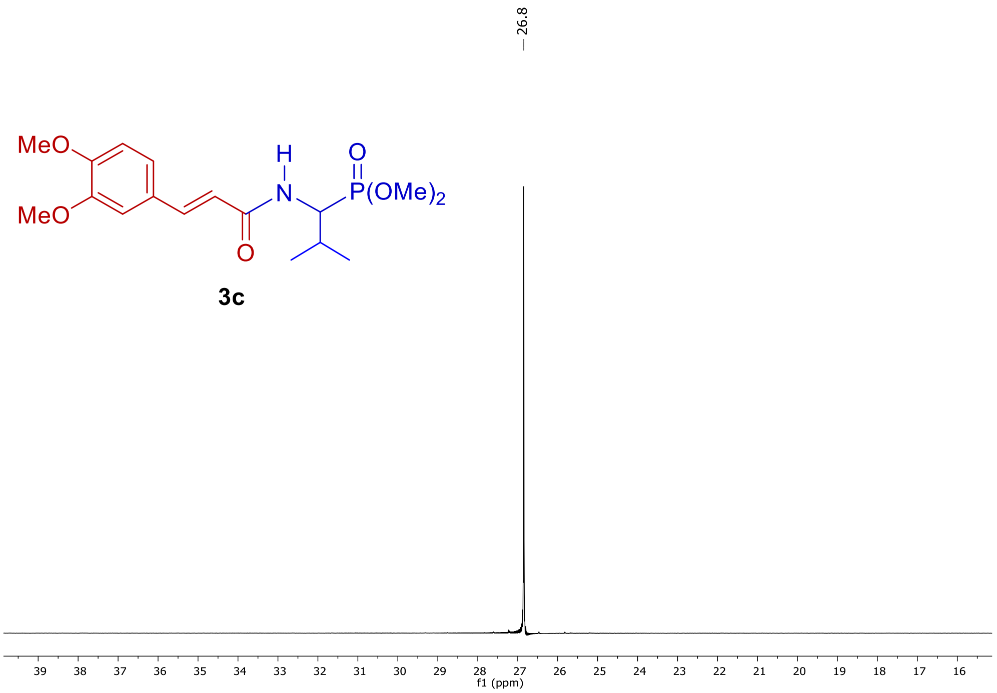

Figure S27. Spectrum of dimethyl *N*-[3-(4,3-dimethoxyphenyl)-2-ene-1-oxo]-2-(methylpropyl)phosphonate **3c** (^{31}P NMR 202 MHz, CDCl$_3$).

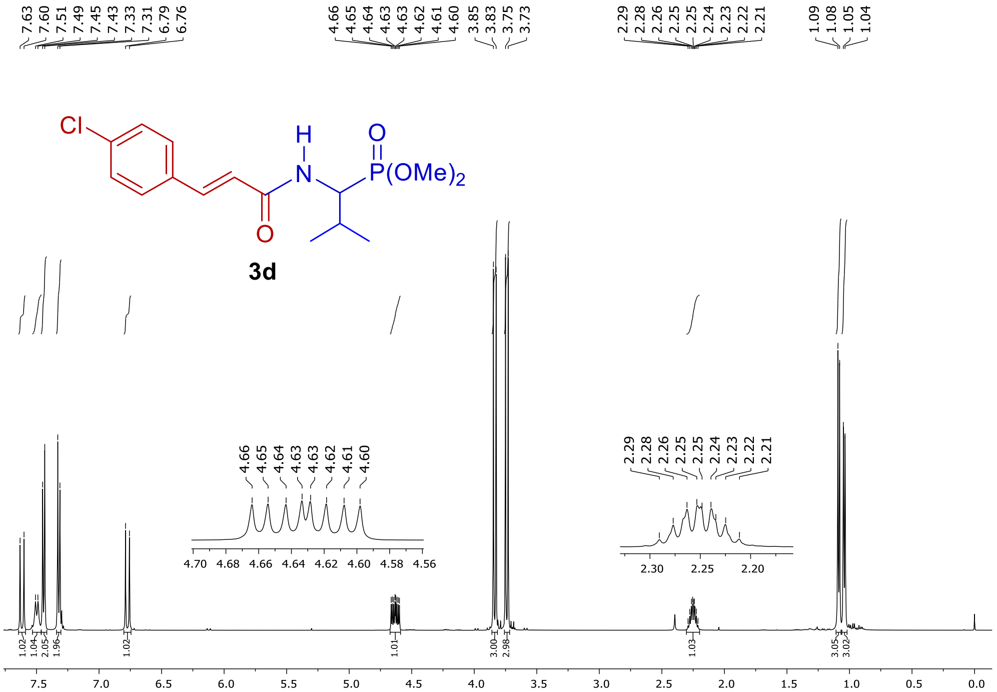

Figure S28. Spectrum of dimethyl *N*-[3-(4-chlorophenyl)-2-ene-1-oxo]-2-(methylpropyl)phosphonate **3d** (^{1}H NMR 500 MHz, CDCl$_3$).

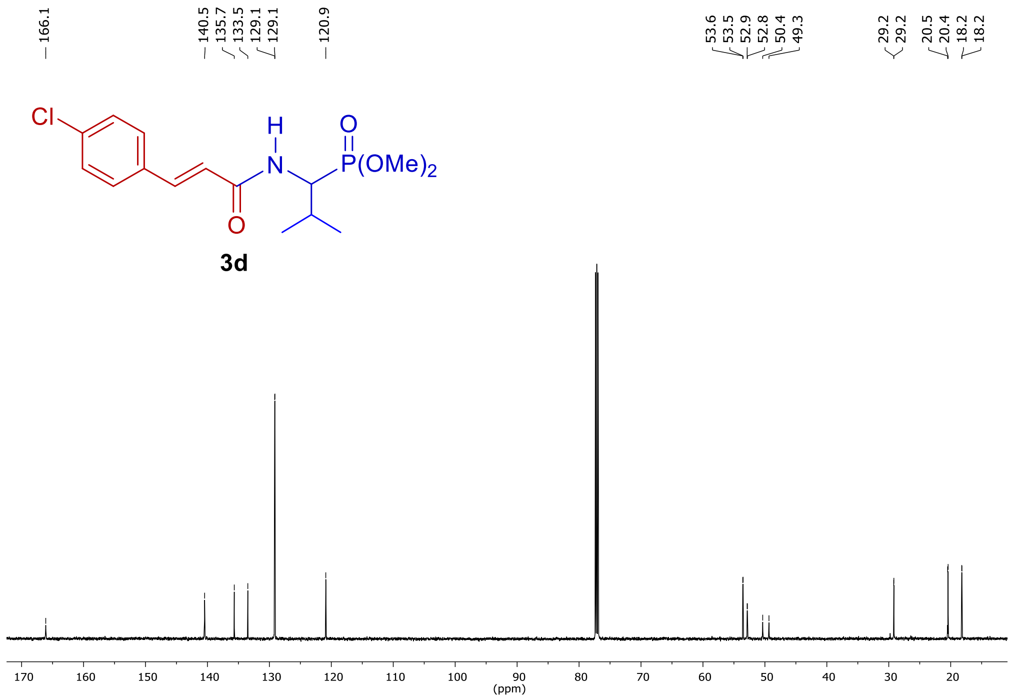

Figure S29. Spectrum of dimethyl *N*-[3-(4-chlorophenyl)-2-ene-1-oxo]-2-(methylpropyl)phosphonate **3d** (^{13}C NMR 151 MHz, $CDCl_3$).

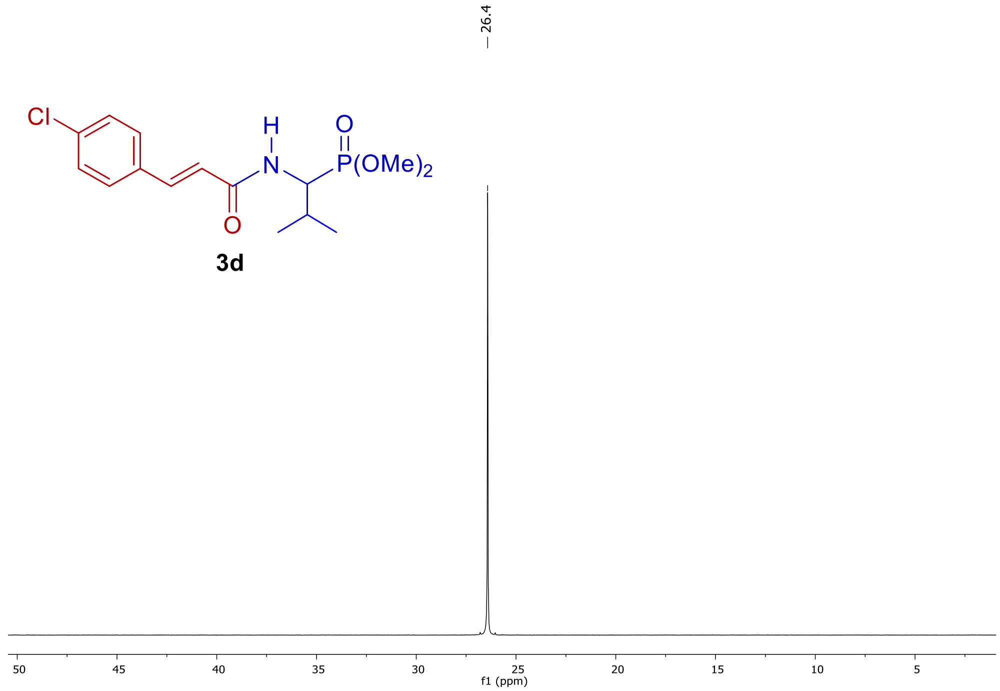

Figure S30. Spectrum of dimethyl *N*-[3-(4-chlorophenyl)-2-ene-1-oxo]-2-(methylpropyl)phosphonate **3d** (^{31}P NMR 202 MHz, $CDCl_3$).

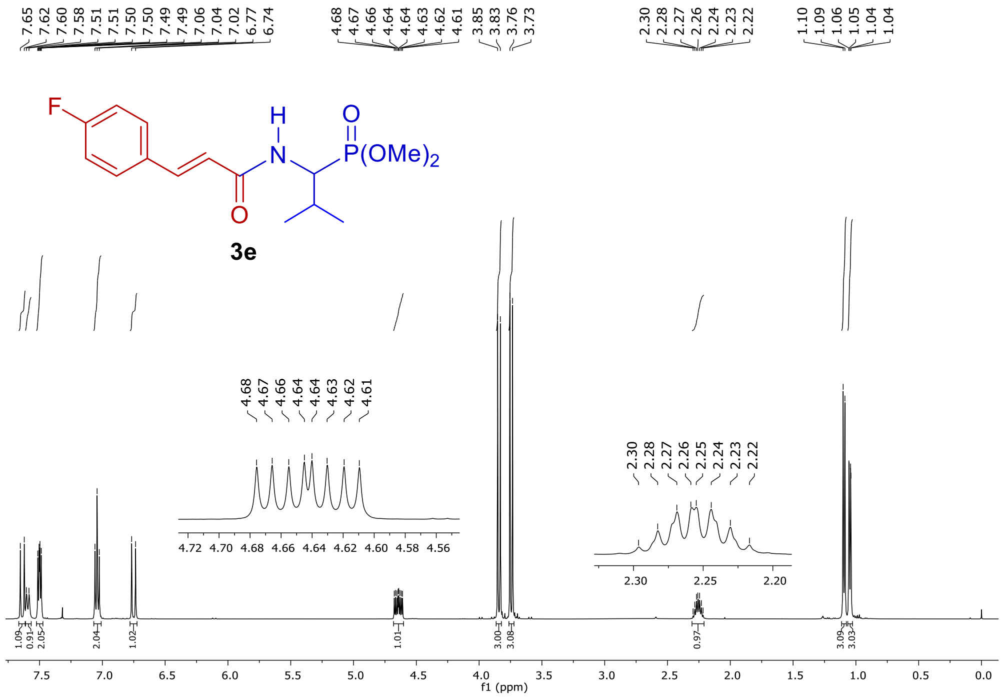

Figure S31. Spectrum of dimethyl *N*-[3-(4-fluorophenyl)-2-ene-1-oxo]-2-(methylpropyl)phosphonate **3e** (^{1}H NMR 500 MHz, $CDCl_3$).

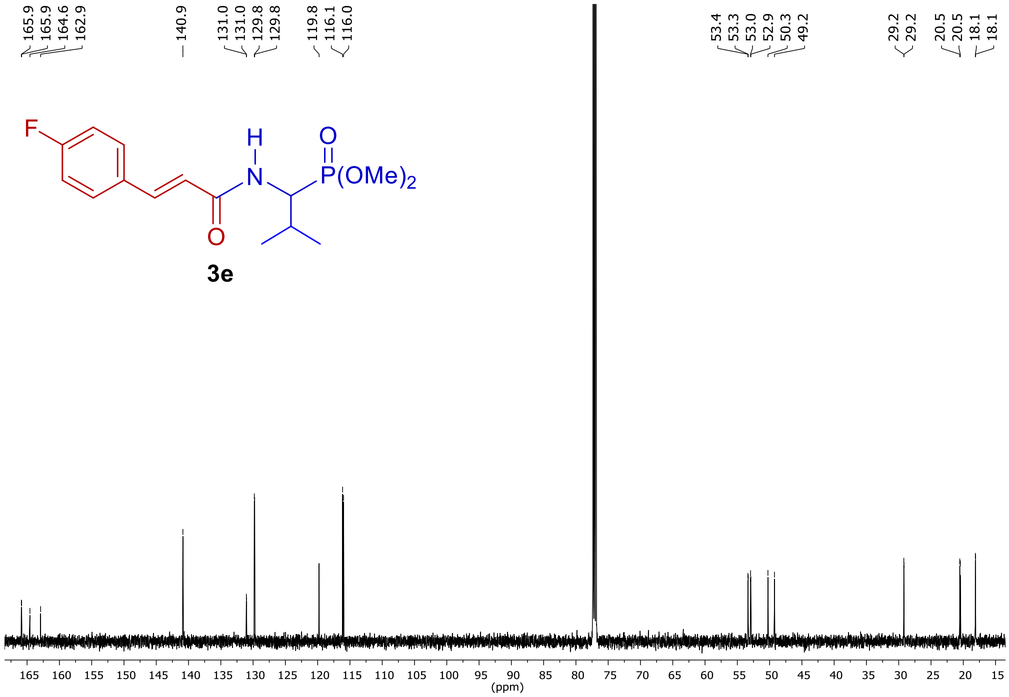

Figure S32. Spectrum of dimethyl *N*-[3-(4-fluorophenyl)-2-ene-1-oxo]-2-(methylpropyl)phosphonate **3e** (^{13}C NMR 151 MHz, $CDCl_3$).

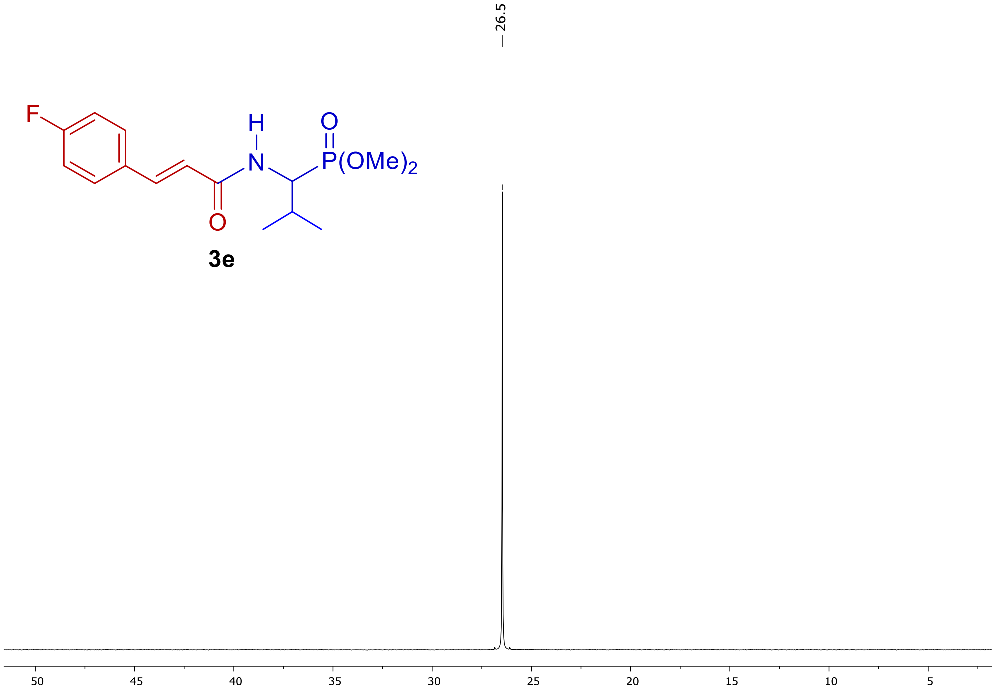

Figure S33. Spectrum of dimethyl *N*-[3-(4-fluorophenyl)-2-ene-1-oxo]-2-(methylpropyl)phosphonate **3e** (^{31}P NMR 202 MHz, $CDCl_3$).

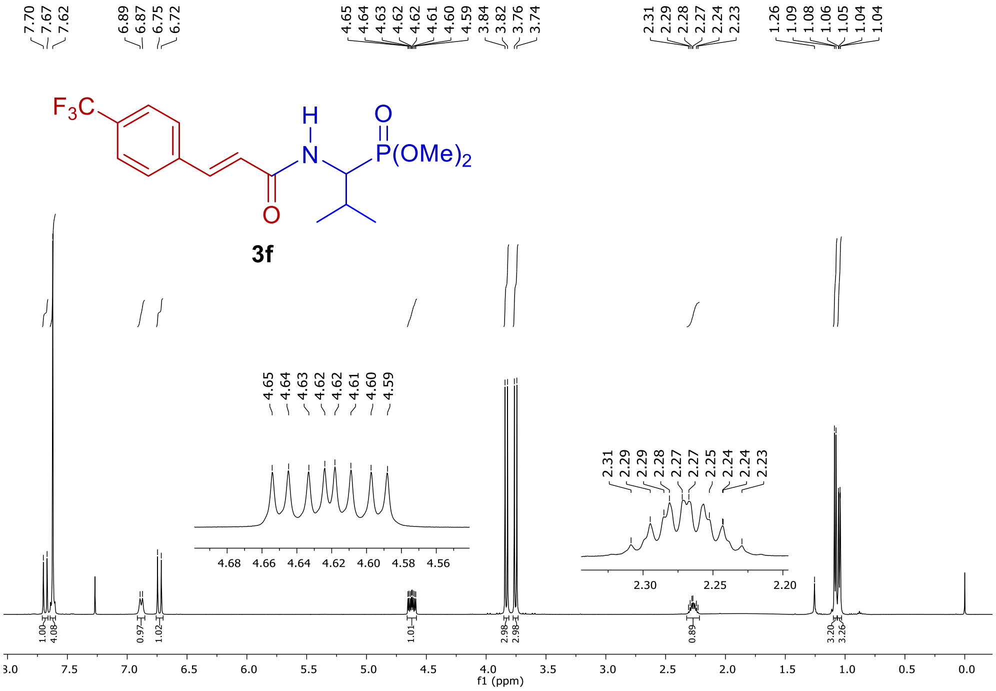

Figure S34. Spectrum of dimethyl *N*-[3-(4-(trifluoromethyl)phenyl)-2-ene-1-oxo]-2-(methylpropyl)phosphonate **3f** (^{1}H NMR 600 MHz, $CDCl_3$).

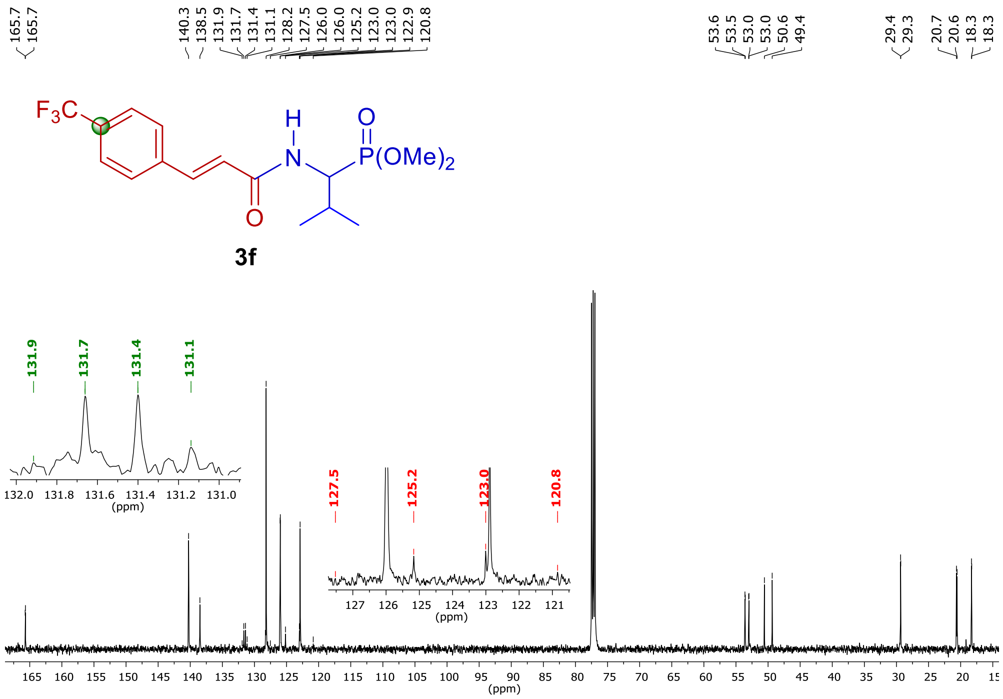

Figure S35. Spectrum of dimethyl *N*-[3-(4-(trifluoromethyl)phenyl)-2-ene-1-oxo]-2-(methylpropyl)phosphonate **3f** (^{13}C NMR 125 MHz, $CDCl_3$).

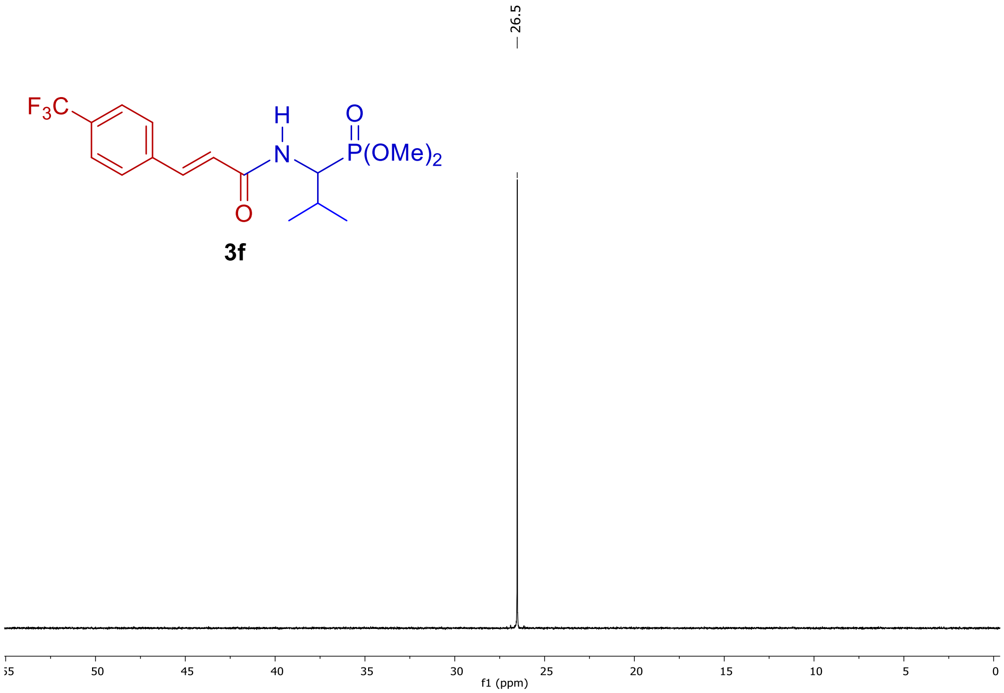

Figure S36. Spectrum of dimethyl *N*-[3-(4-(trifluoromethyl)phenyl)-2-ene-1-oxo]-2-(methylpropyl)phosphonate **3f** (^{31}P NMR 243 MHz, $CDCl_3$).

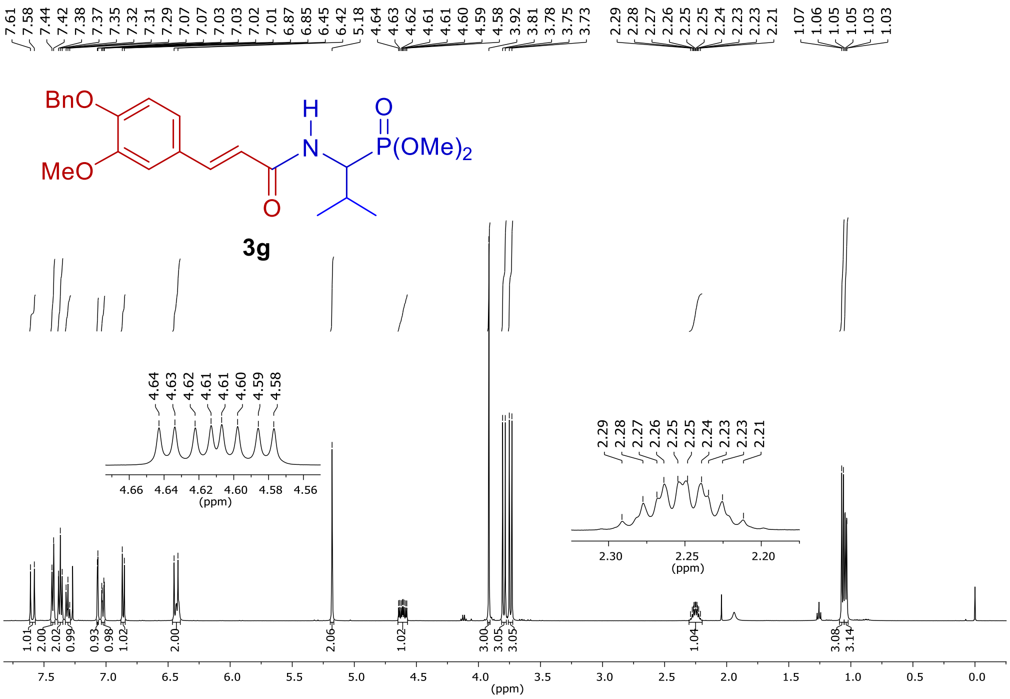

Figure S37. Spectrum of dimethyl *N*-[3-((4-benzyloxi)-3-methoxyphenyl)-2-ene-1-oxo]-2-(methylpropyl)phosphonate **3g** (^{1}H NMR 500 MHz, $CDCl_3$).

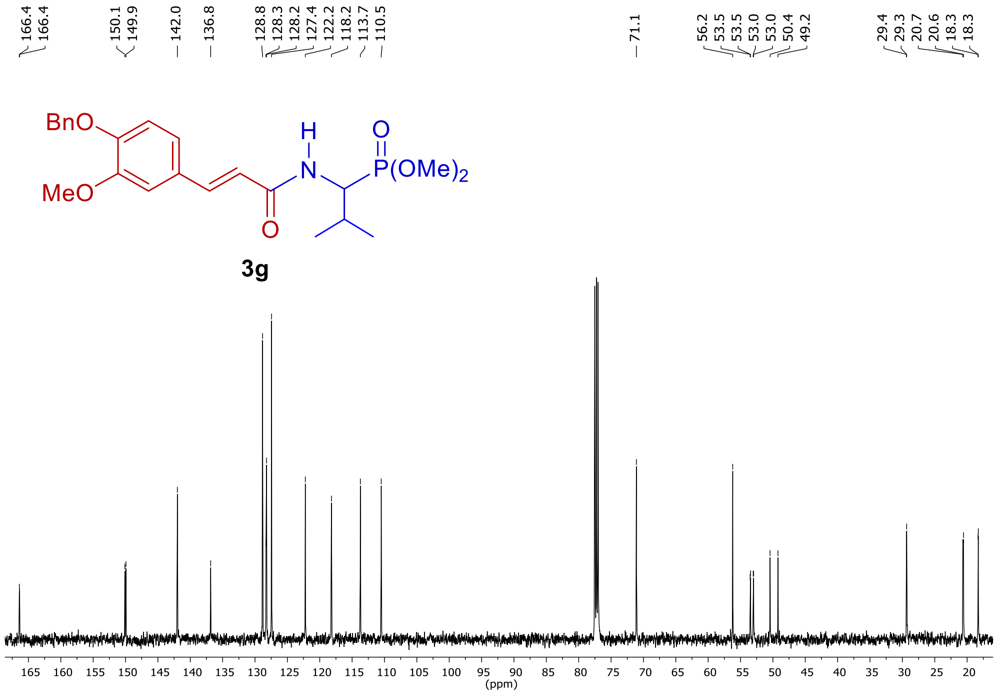

Figure S38. Spectrum of dimethyl *N*-[3-((4-benzyloxi)-3-methoxyphenyl)-2-ene-1-oxo]-2-(methylpropyl)phosphonate **3g** (^{13}C NMR 125 MHz, $CDCl_3$).

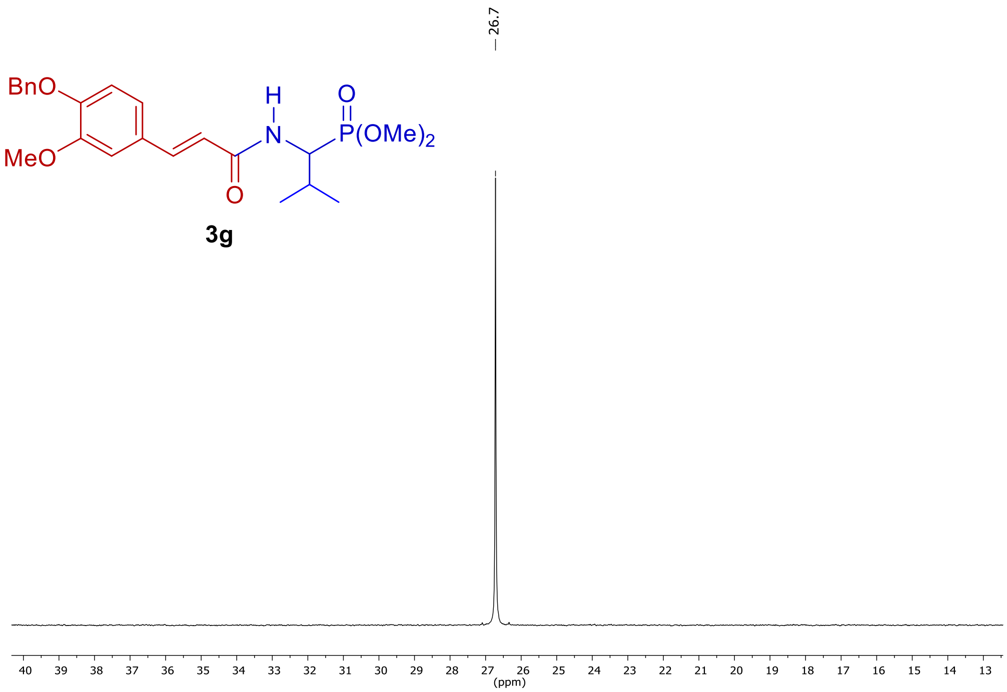

Figure S39. Spectrum of dimethyl *N*-[3-((4-benzyloxi)-3-methoxyphenyl)-2-ene-1-oxo]-2-(methylpropyl)phosphonate **3g** (^{31}P NMR (202 MHz, $CDCl_3$).

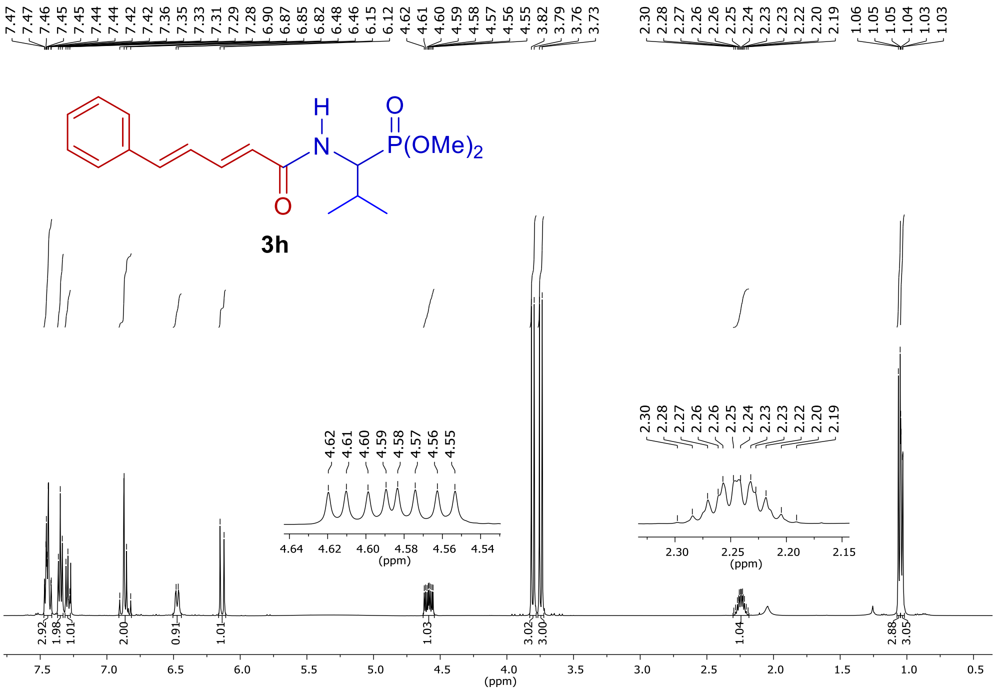

Figure S40. Spectrum of dimethyl *N*-[(2*E*,4*E*)-5-phenylpenta-2,4-dien-1-oxo]-2-(methylpropyl)phosphonate **3h** (^{1}H NMR 500 MHz, $CDCl_3$).

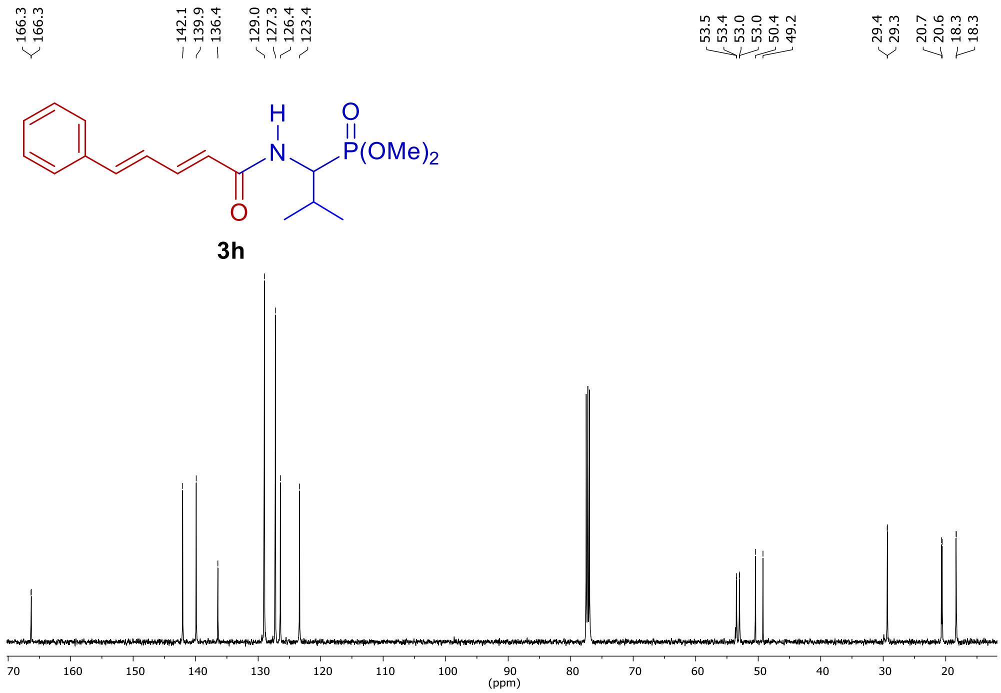

Figure S41. Spectrum of dimethyl *N*-[(2*E*,4*E*)-5-phenylpenta-2,4-dien-1-oxo]-2-(methylpropyl)phosphonate **3h** (^{13}C NMR 125 MHz, $CDCl_3$).

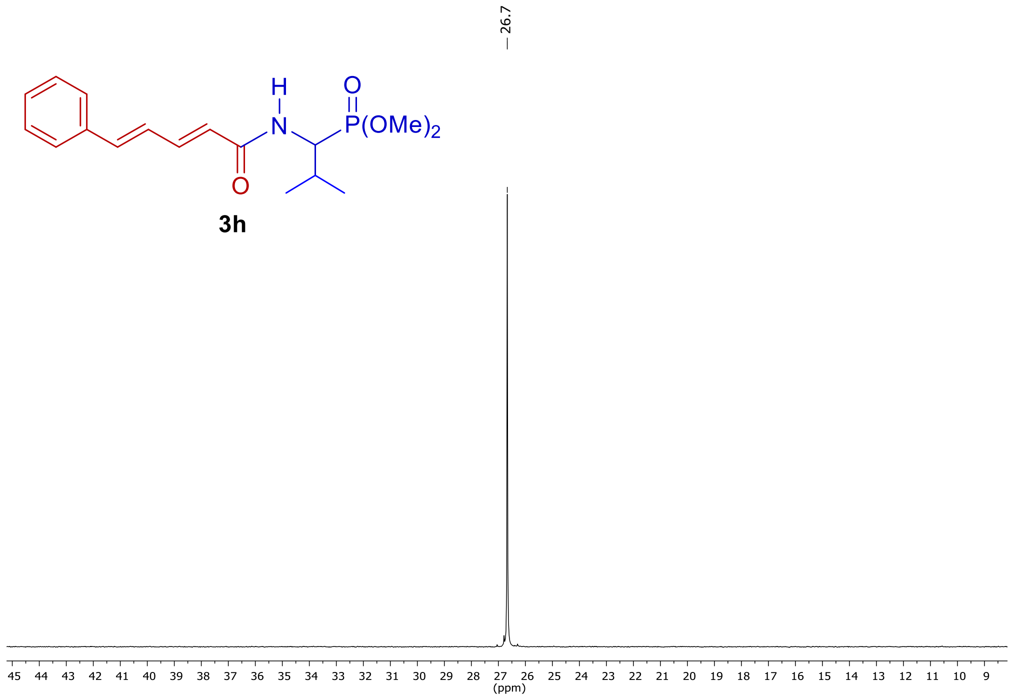

Figure S42. Spectrum of dimethyl *N*-[(2*E*,4*E*)-5-phenylpenta-2,4-dien-1-oxo]-2-(methylpropyl)phosphonate **3h** (^{31}P NMR 202 MHz, $CDCl_3$).

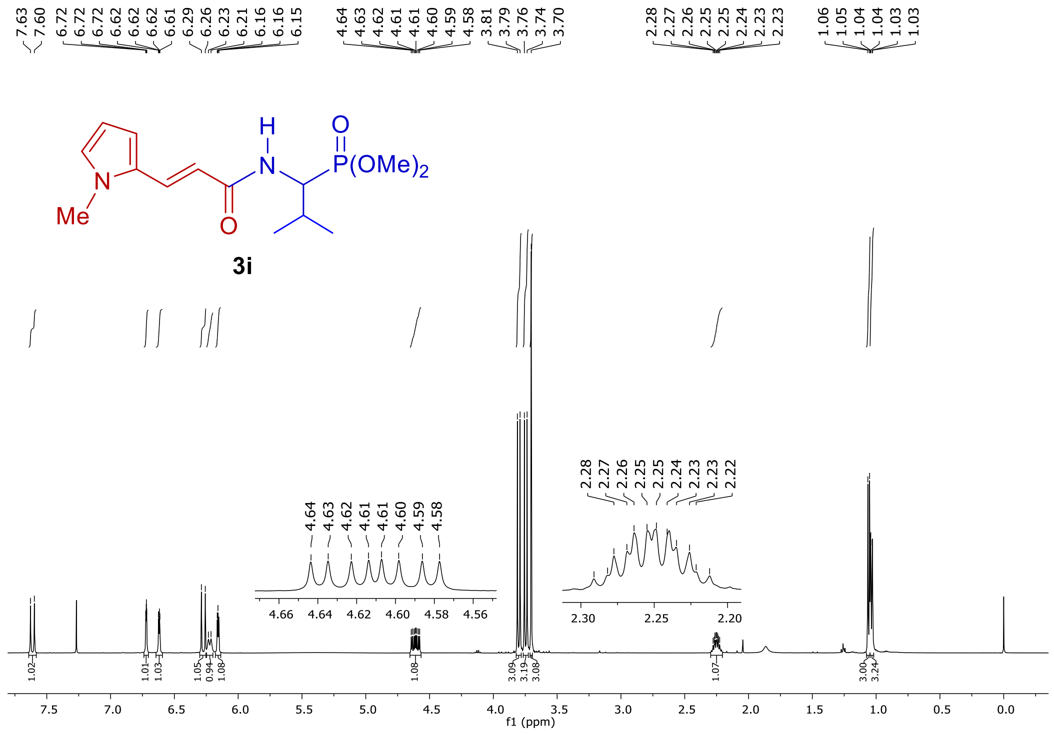

Figure S43. Spectrum of dimethyl *N*-[*N*-methylpyrrol-2-ene-1-oxo]-2-(methylpropyl)phosphonate **3i** (^{1}H NMR 500 MHz, $CDCl_3$).

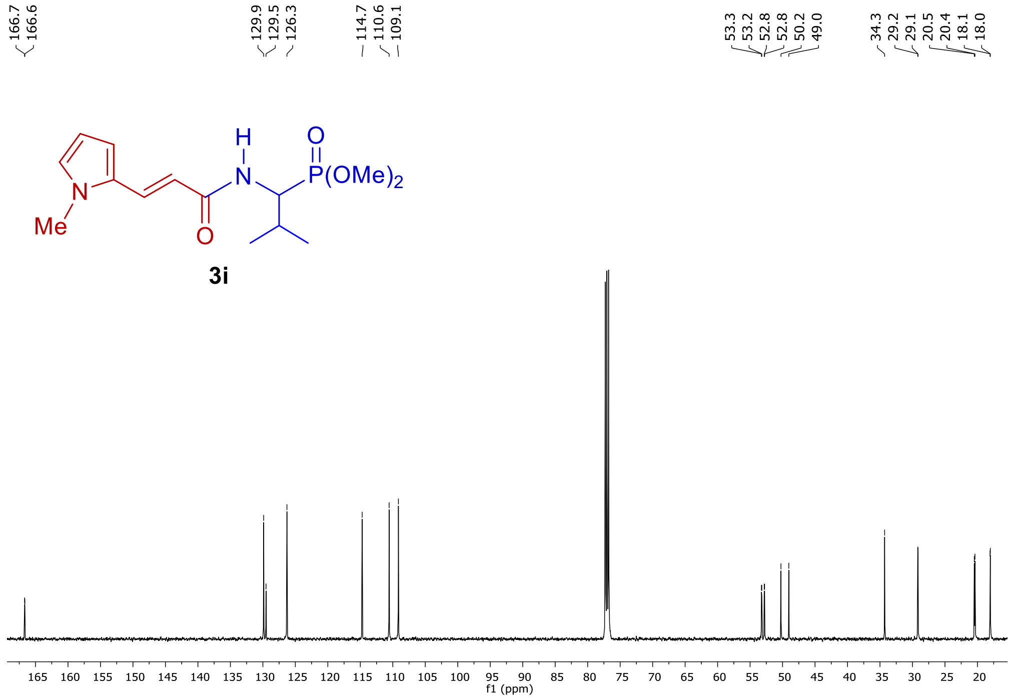

Figure S44. Spectrum of dimethyl *N*-[*N*-methylpyrrol-2-ene-1-oxo]-2-(methylpropyl)phosphonate **3i** (^{13}C NMR 125 MHz, $CDCl_3$).

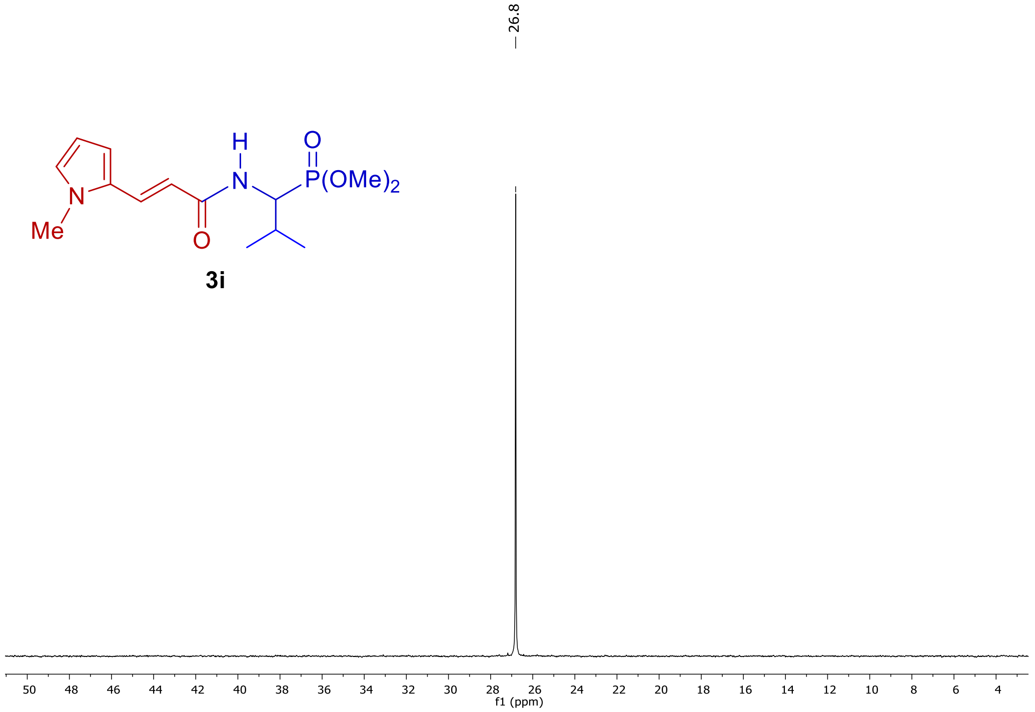

Figure S45. Spectrum of dimethyl *N*-[*N*-methylpyrrol-2-ene-1-oxo]-2-(methylpropyl)phosphonate **3i** (^{31}P NMR 202 MHz, $CDCl_3$).

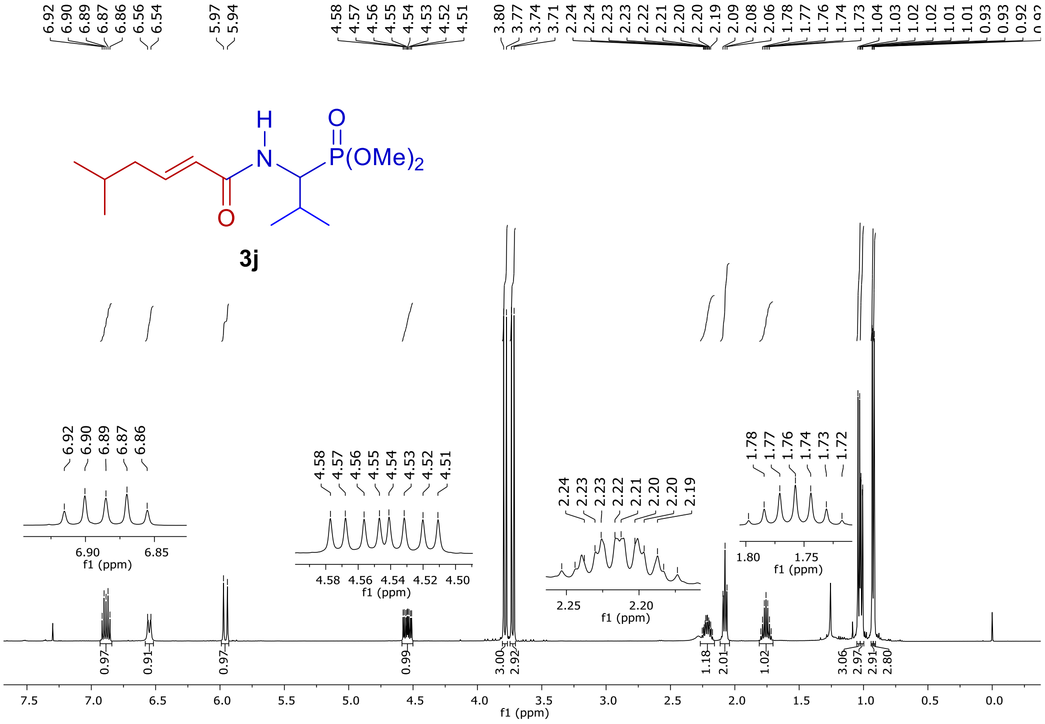

Figure S46. Spectrum of dimethyl *N*-[5-methylhex-2-ene-1-oxo]-2-(methylpropyl)phosphonate **3j** (^{1}H NMR 500 MHz, $CDCl_3$).

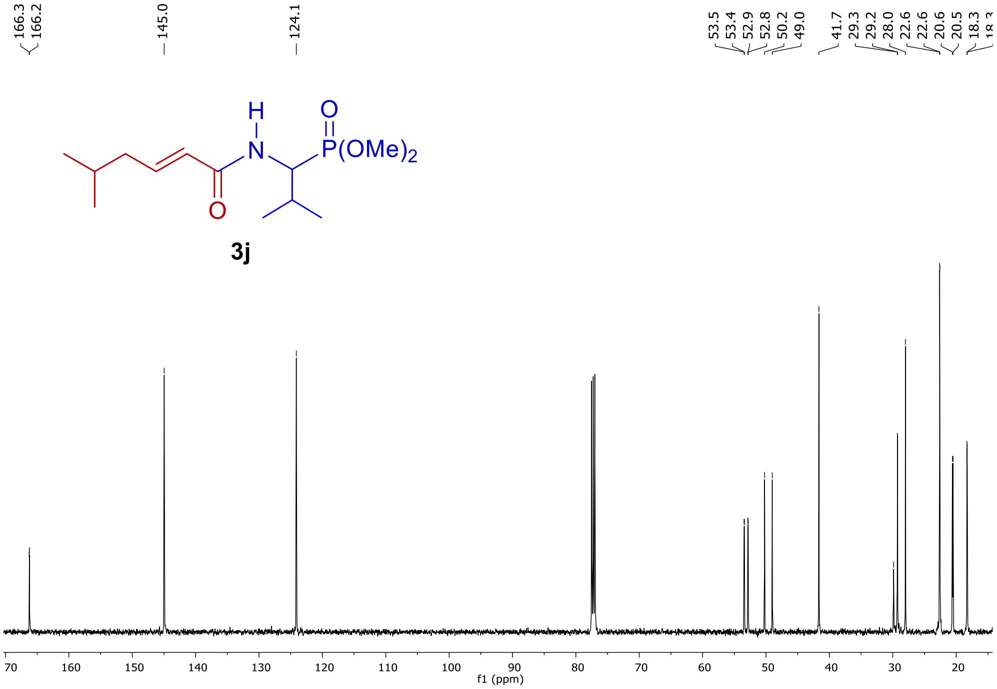

Figure S47. Spectrum of dimethyl *N*-[5-methylhex-2-ene-1-oxo]-2-(methylpropyl)phosphonate **3j** (^{13}C NMR 125 MHz, $CDCl_3$).

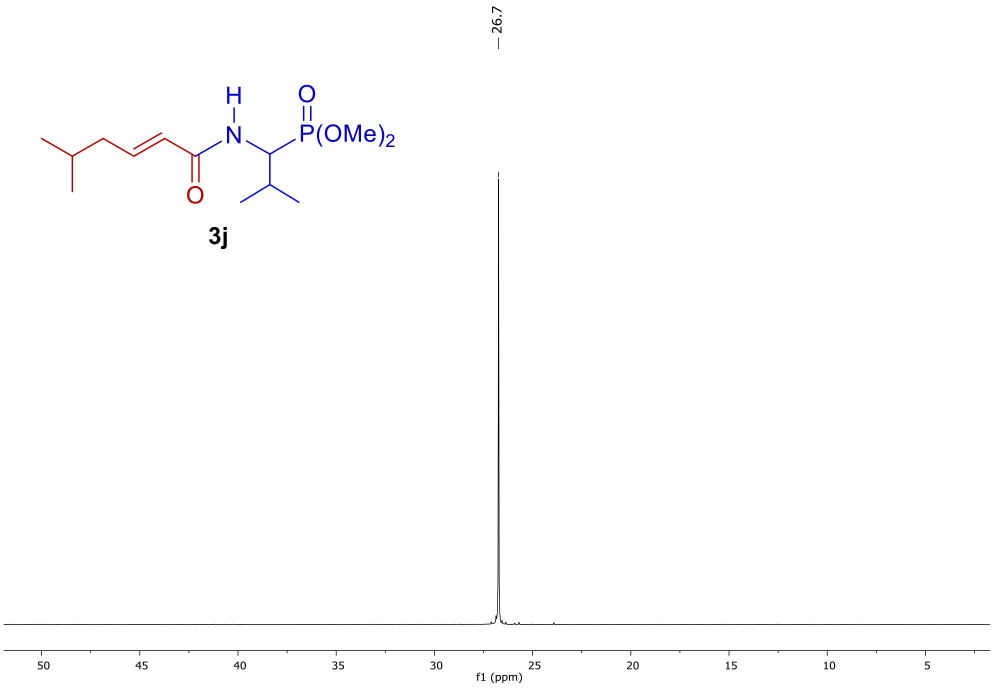

Figure S48. Spectrum of dimethyl *N*-[5-methylhex-2-ene-1-oxo]-2-(methylpropyl)phosphonate **3j** (^{31}P NMR 202 MHz, $CDCl_3$).

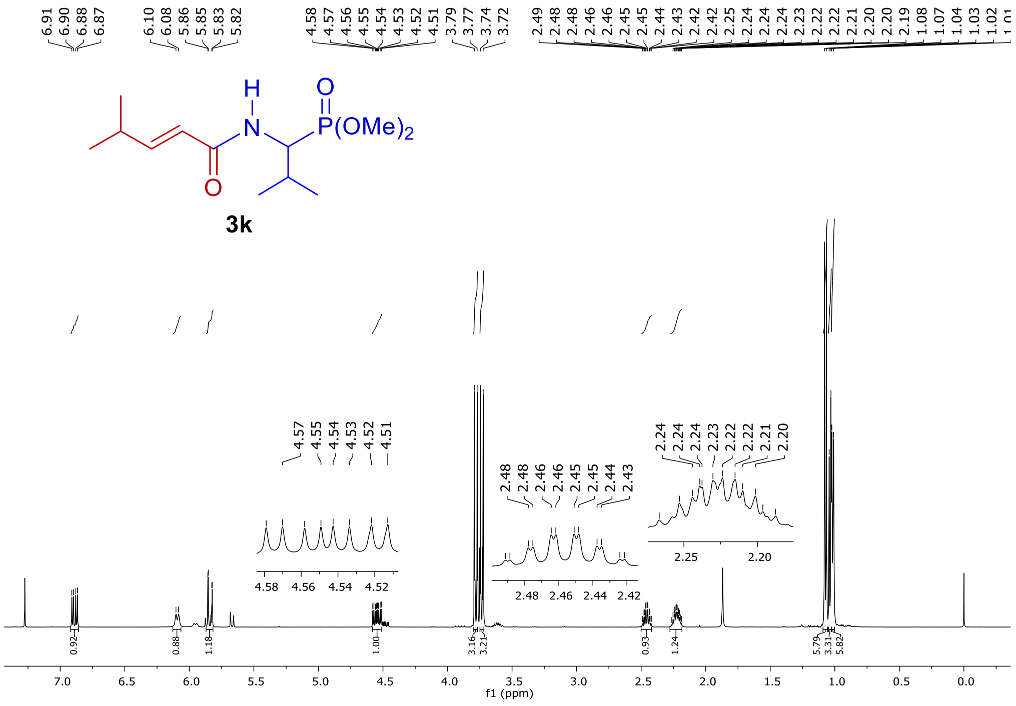

Figure S49. Spectrum of the mixture 85:15 *E:Z* ratio of dimethyl *N*-[4-methylpent-2-ene-1-oxo]-2-(methylpropyl)phosphonate **3k** (^{1}H NMR 500 MHz, $CDCl_3$).

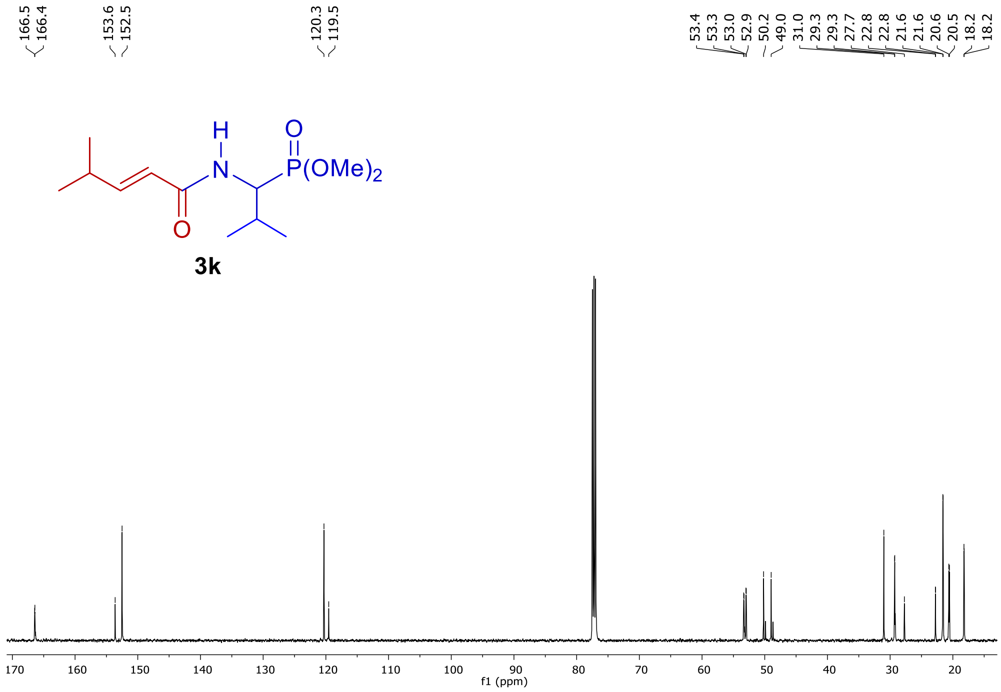

Figure S50. Spectrum of the mixture 85:15 *E:Z* ratio of dimethyl *N*-[4-methylpent-2-ene-1-oxo]-2-(methylpropyl)phosphonate **3k** (^{13}C NMR 125 MHz, $CDCl_3$).

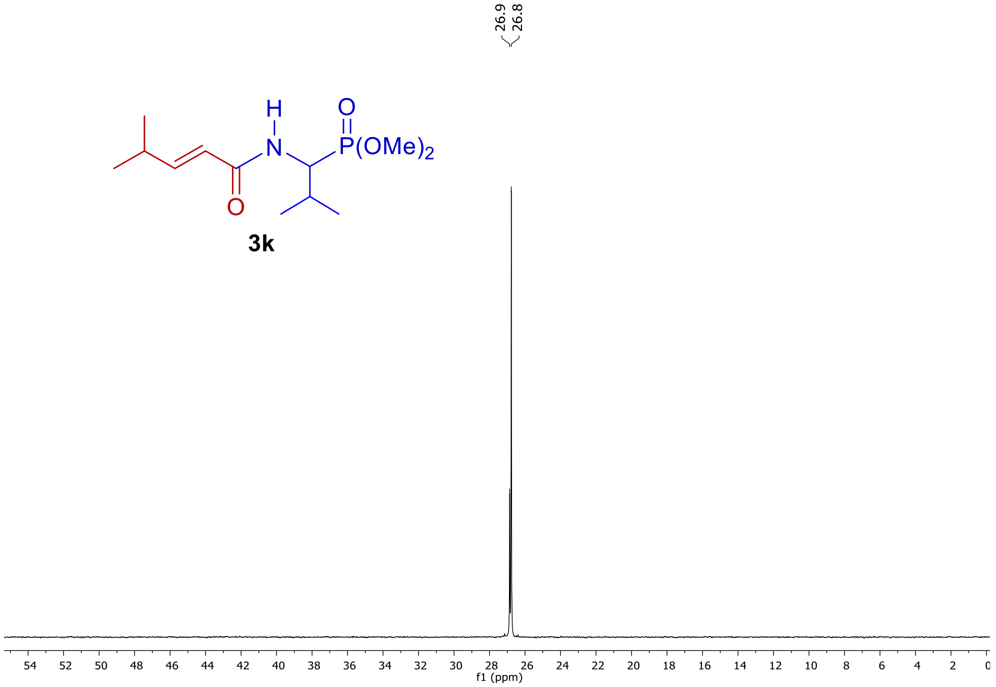

Figure S51. Spectrum of the mixture 85:15 *E:Z* ratio of dimethyl *N*-[4-methylpent-2-ene-1-oxo]-2-(methylpropyl)phosphonate **3k** (^{31}P NMR 202 MHz, $CDCl_3$).

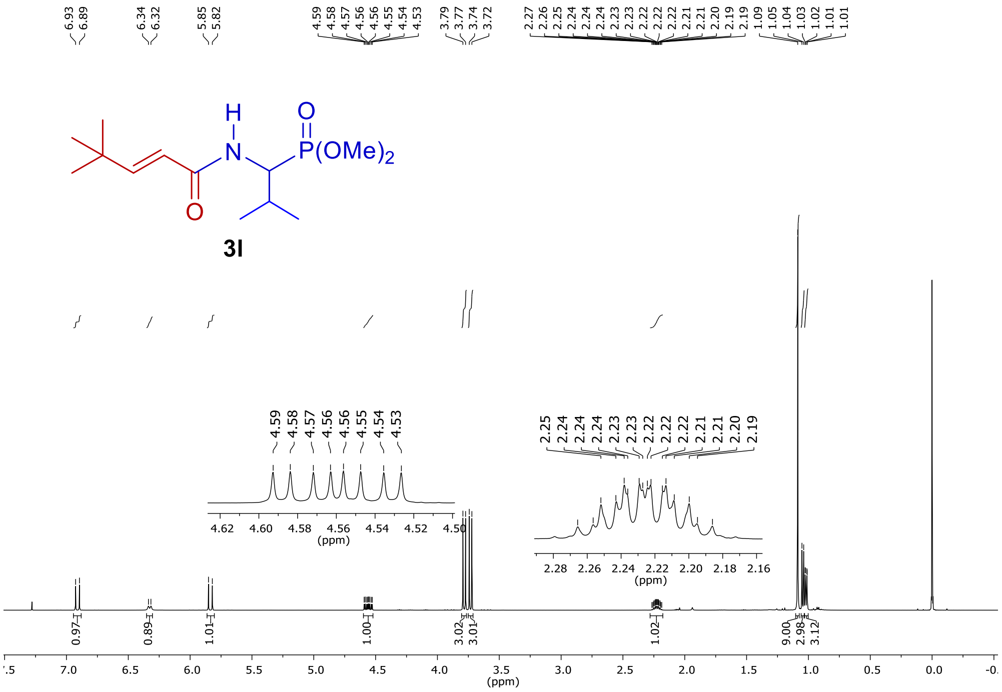

Figure S52. Spectrum of dimethyl *N*-[4,4-dimethylpent-2-ene-1-oxo]-2-(methylpropyl)phosphonate **3l** (^1H NMR 500 MHz, $CDCl_3$).

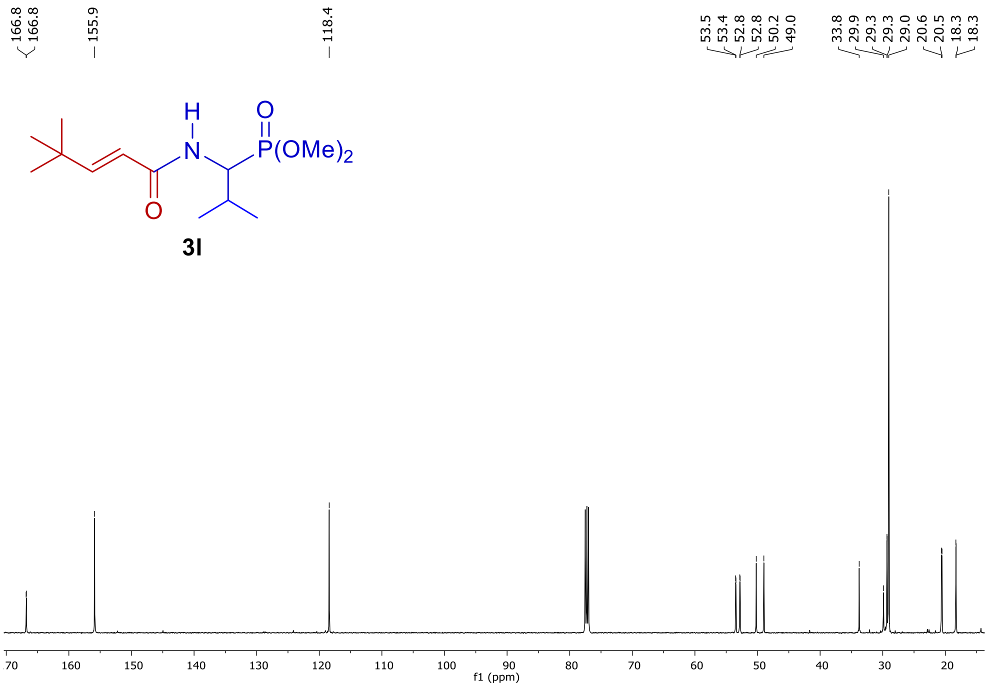

Figure S53. Spectrum of dimethyl *N*-[4,4-methylpent-2-ene-1-oxo]-2-(methylpropyl)phosphonate **3l** (^{13}C NMR 125 MHz, $CDCl_3$).

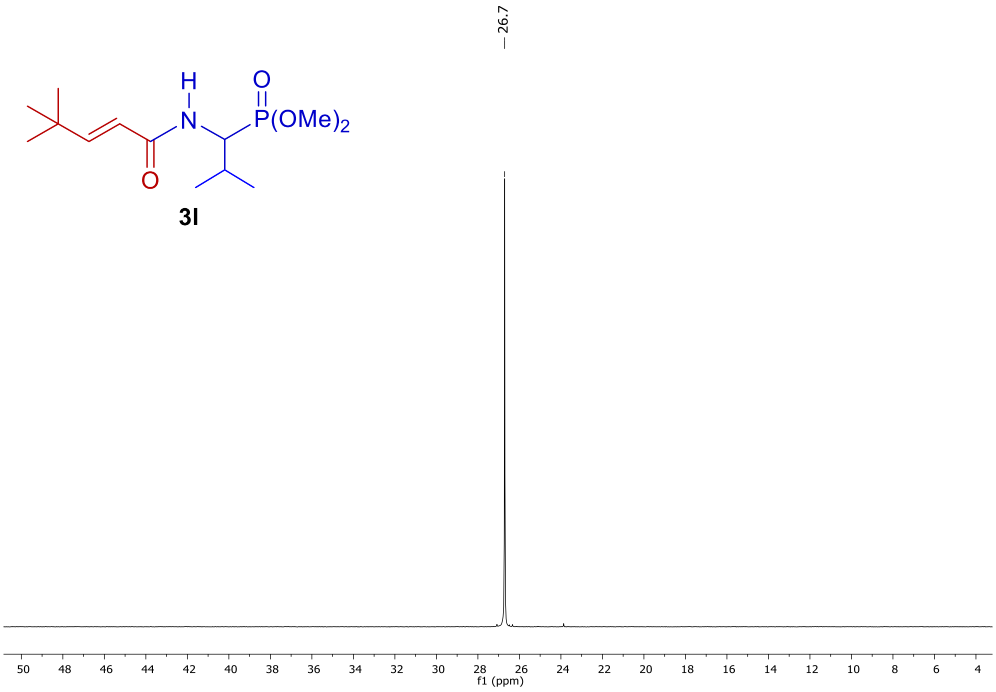

Figure S54. Spectrum of dimethyl *N*-[4,4-dimethylpent-2-ene-1-oxo]-2-(methylpropyl)phosphonate **3l** (^{31}P NMR 202 MHz, $CDCl_3$).

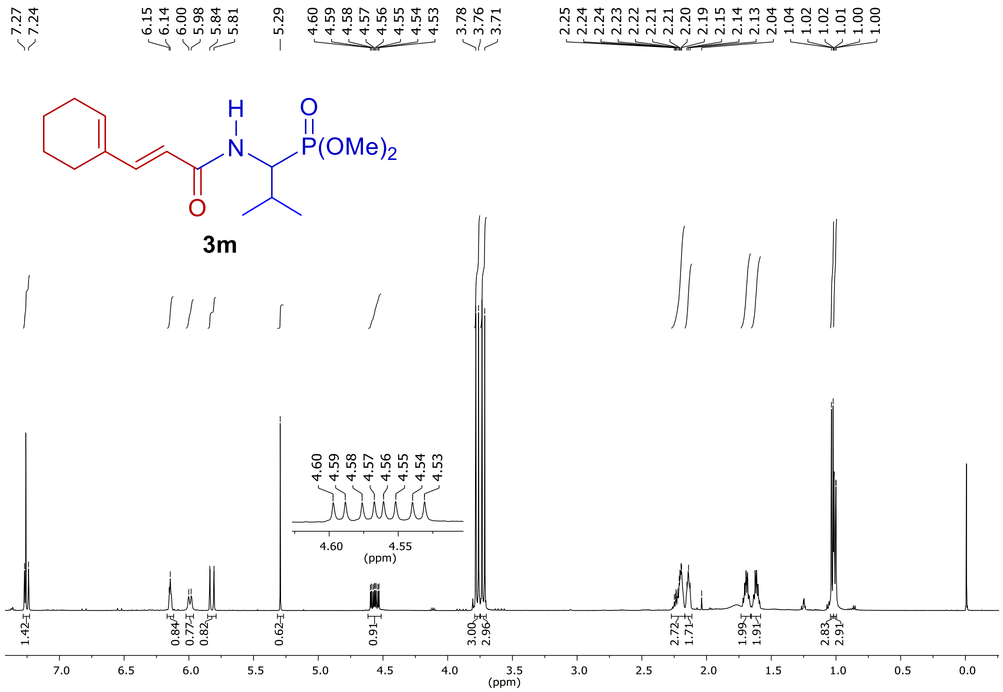

Figure S55. Spectrum of dimethyl *N*-[1-(3-cyclohex-1-ene)-1-oxo]-2-(methylpropyl)phosphonate **3m** (^{1}H NMR 500 MHz, $CDCl_3$).

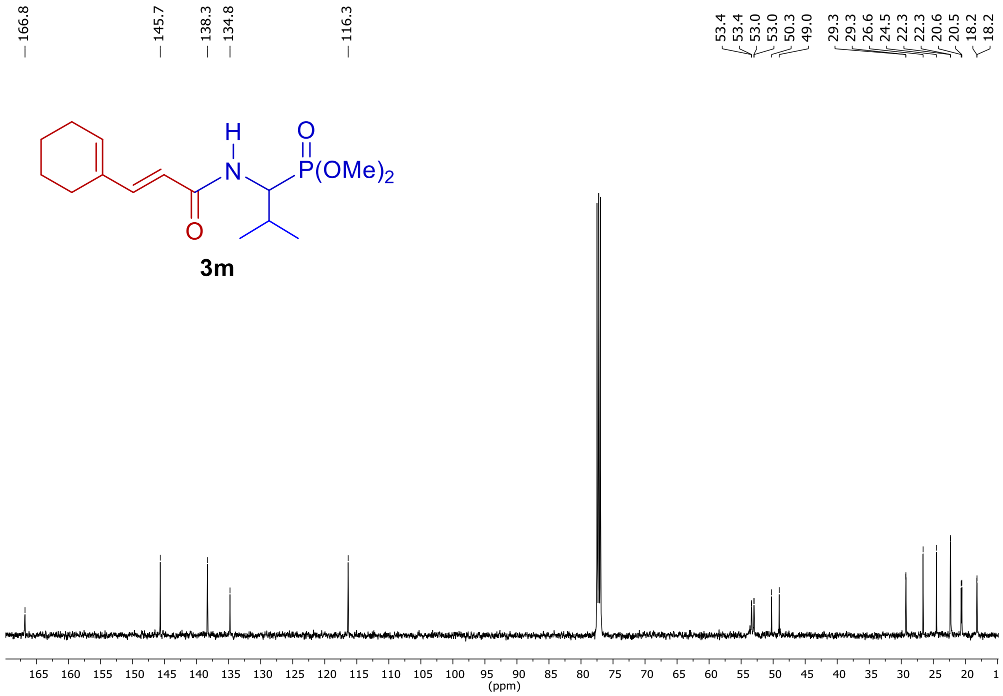

Figure S56. Spectrum of dimethyl *N*-[1-(3-(cyclohex-1-ene)-1-oxo]-2-(methylpropyl)phosphonate **3m** (^{13}C NMR 125 MHz, $CDCl_3$).

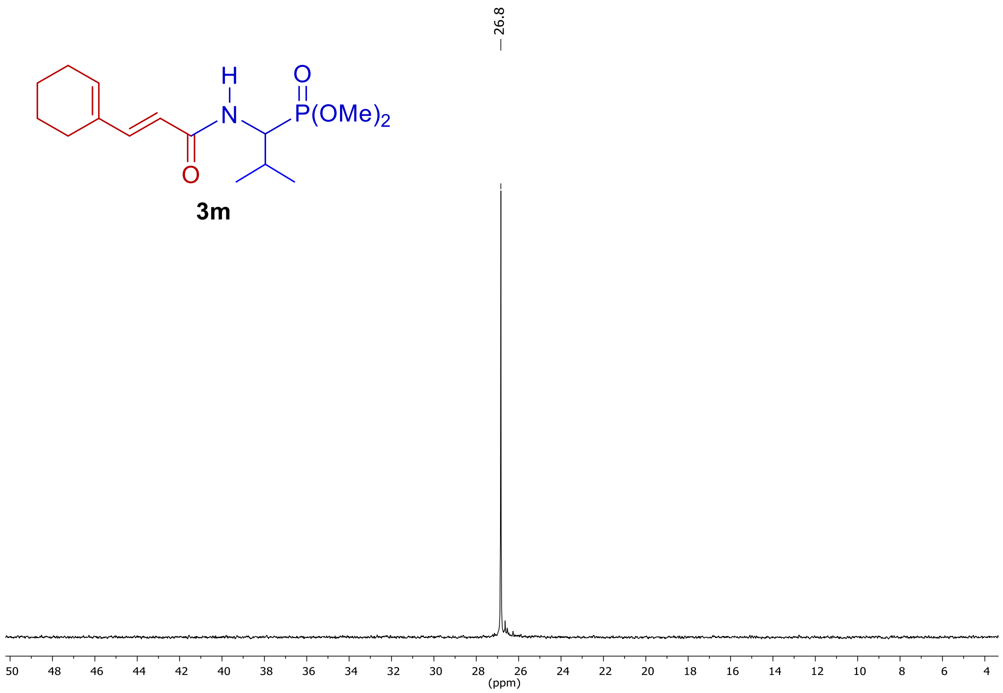

Figure S57. Spectrum of dimethyl *N*-[1-(3-(cyclohex-1-ene)-1-oxo]-2-(methylpropyl)phosphonate **3m** (^{31}P NMR 202 MHz, $CDCl_3$).

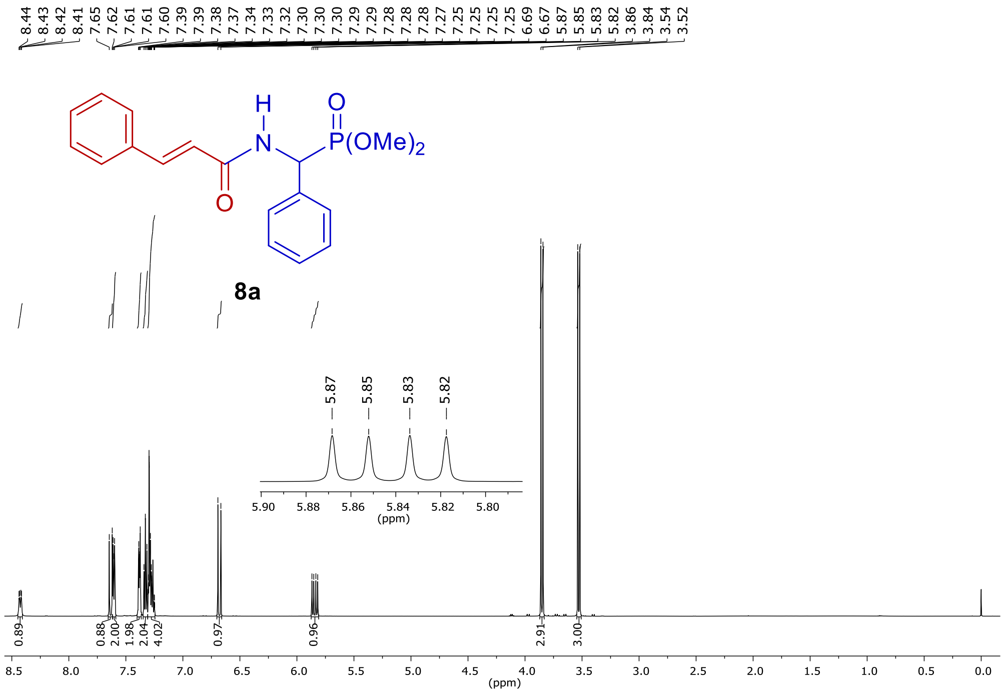

Figure S58. Spectrum of dimethyl *N*-[3-phenyl-2-ene-1-oxo]-(phenyl-methyl)phosphonate **8a** (^{1}H NMR 500 MHz, $CDCl_3$).

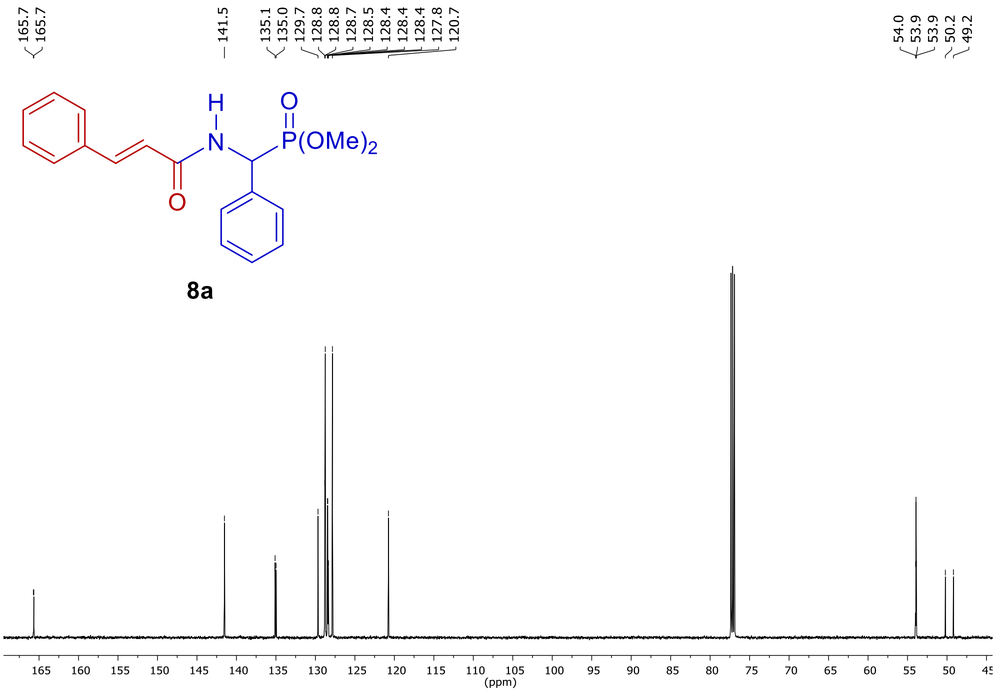

Figure S59. Spectrum of dimethyl *N*-[3-phenyl-2-ene-1-oxo]-(phenyl-methyl)phosphonate **8a** (^{13}C NMR 125 MHz, $CDCl_3$).

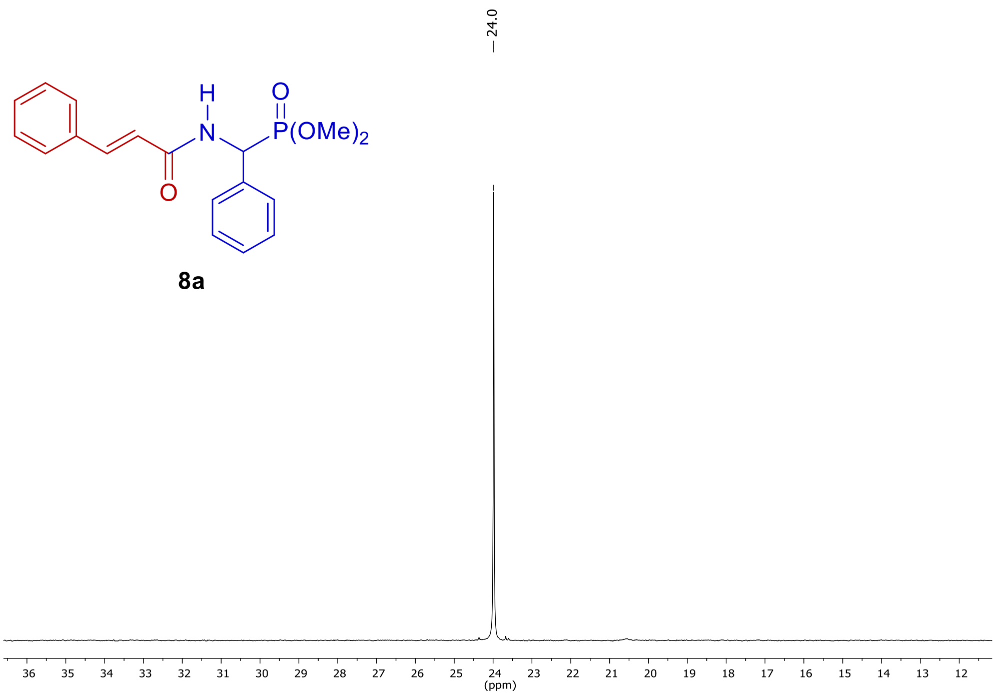

Figure S60. Spectrum of dimethyl *N*-[3-phenyl-2-ene-1-oxo]-(phenyl-methyl)phosphonate **8a** (^{31}P NMR 202 MHz, $CDCl_3$).

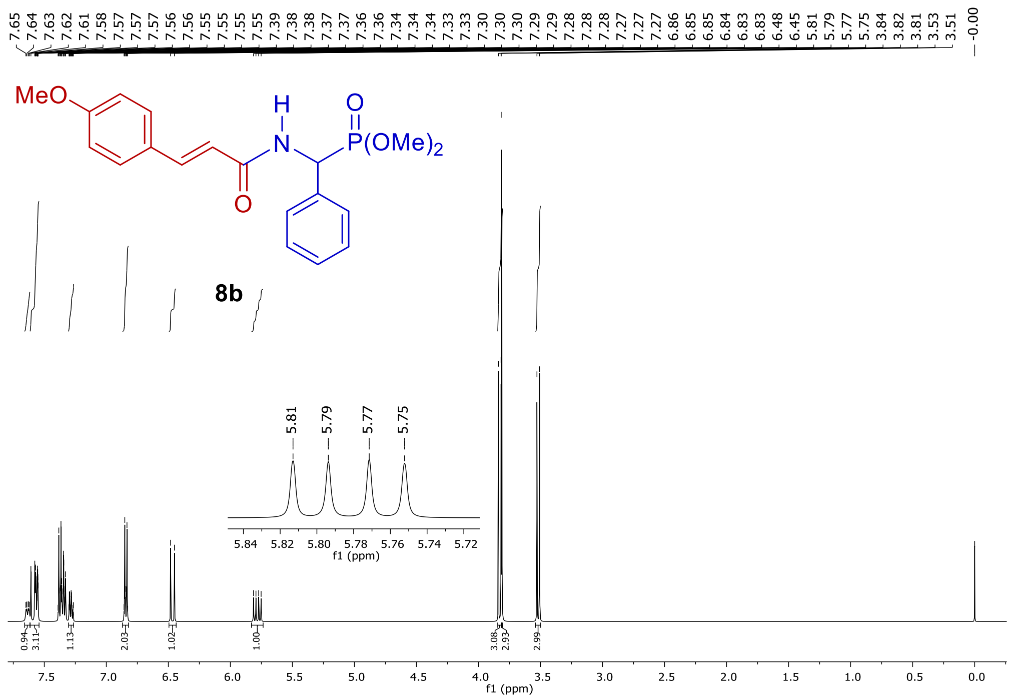

Figure S61. Spectrum of dimethyl *N*-[3-(4-methoxyphenyl)-2-ene-1-oxo]-(phenyl-methyl)phosphonate **8b** (^{1}H NMR 500 MHz, $CDCl_3$).

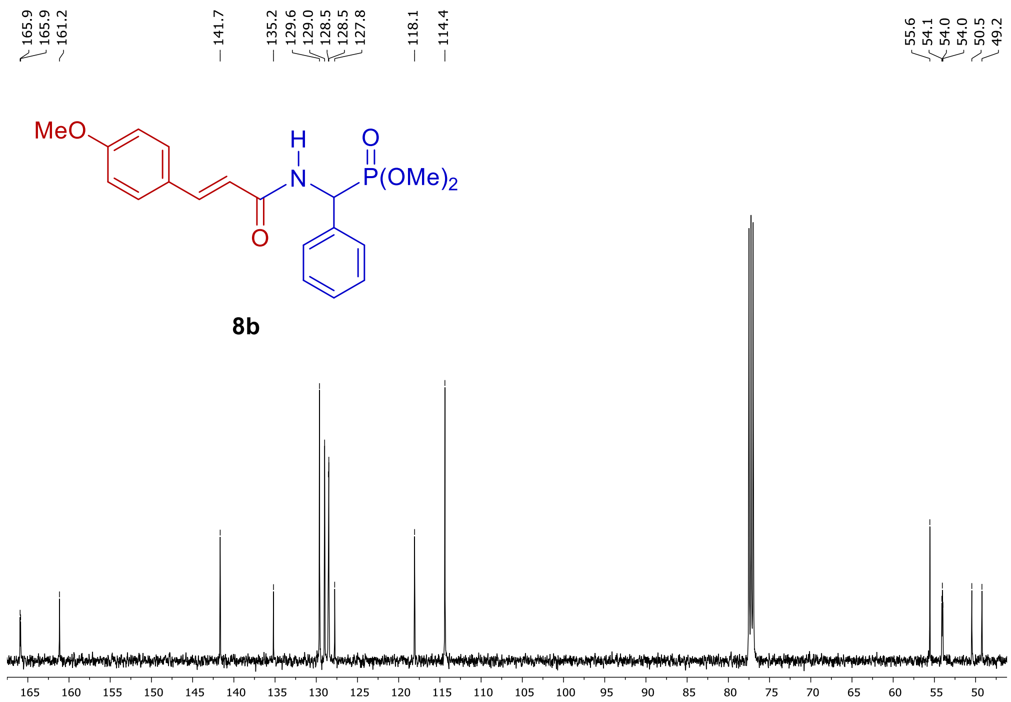

Figure S62. Spectrum of dimethyl *N*-[3-(4-methoxyphenyl)-2-ene-1-oxo]-(phenyl-methyl)phosphonate **8b** (^{13}C NMR 125 MHz, $CDCl_3$).

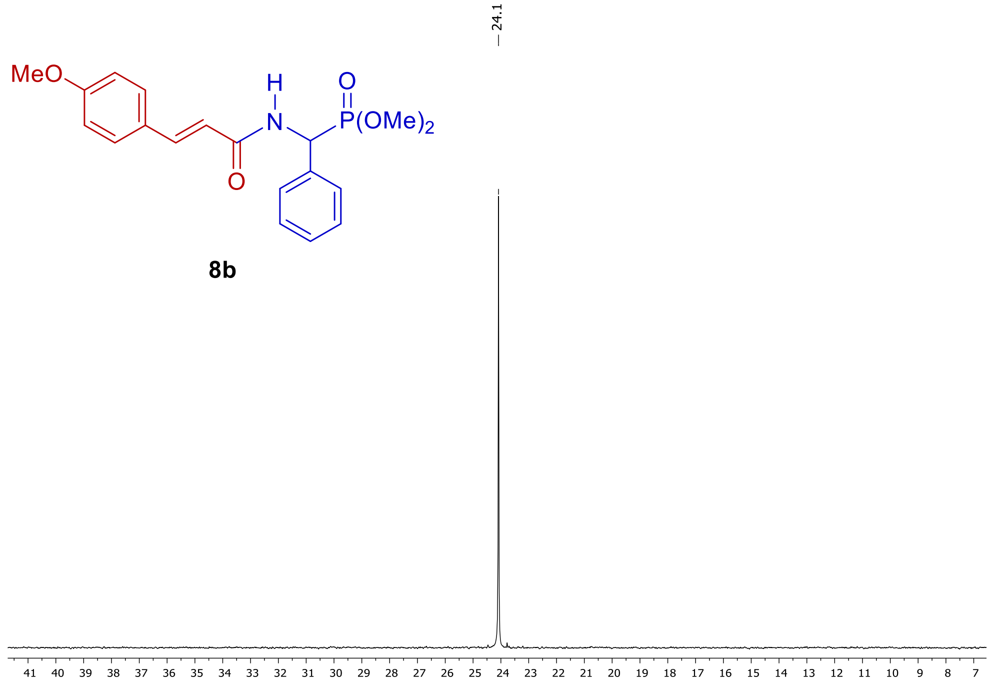

Figure S63. Spectrum of dimethyl *N*-[3-(4-methoxyphenyl)-2-ene-1-oxo]-(phenyl-methyl)phosphonate **8b** (^{31}P NMR 202 MHz, $CDCl_3$).

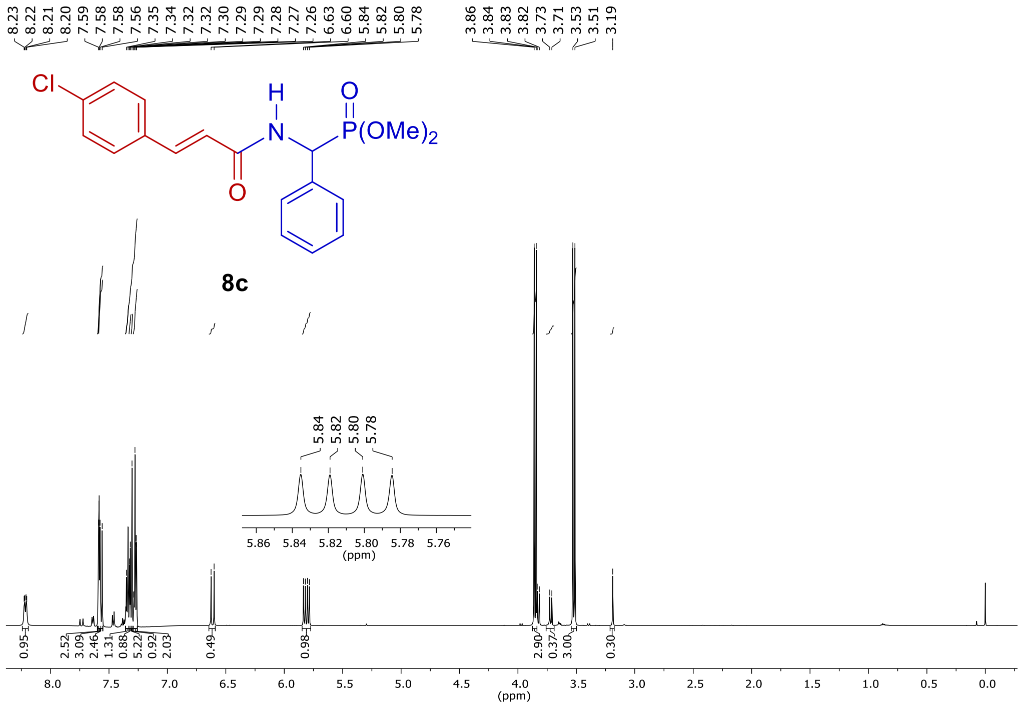

Figure S64. Spectrum of dimethyl *N*-[3-(4-chlorophenyl)-2-ene-1-oxo]-(phenyl-methyl)phosphonate **8c** (^{1}H NMR 500 MHz, $CDCl_3$).

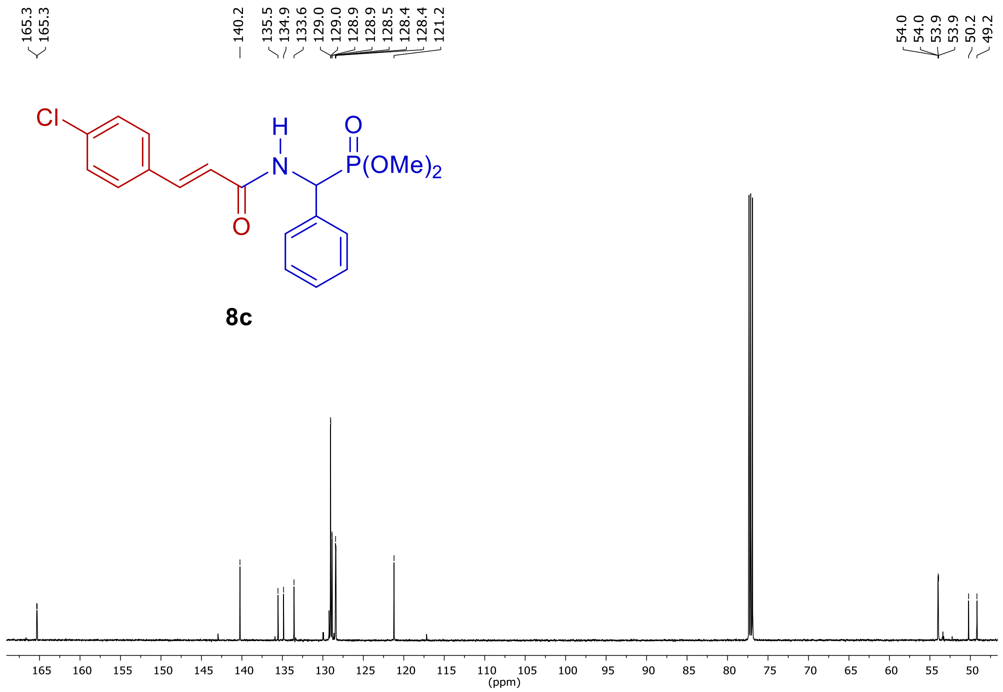

Figure S65. Spectrum of dimethyl *N*-[3-(4-chlorophenyl)-2-ene-1-oxo]-(phenyl-methyl)phosphonate **8c** (^{13}C NMR 125 MHz, $CDCl_3$).

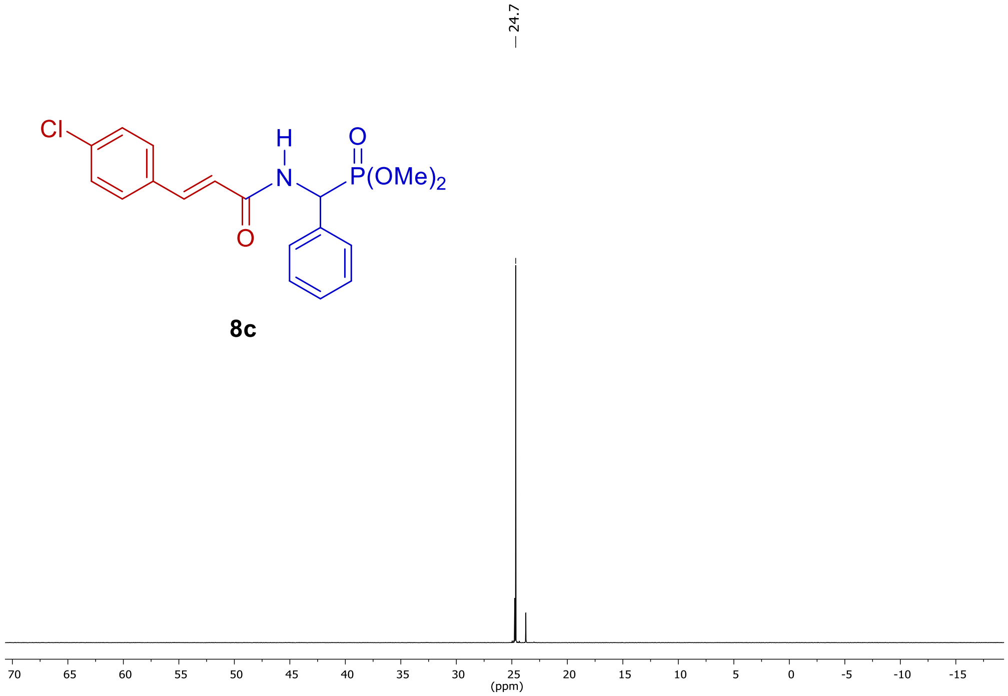

Figure S66. Spectrum of dimethyl *N*-[3-(4-chlorophenyl)-2-ene-1-oxo]-(phenyl-methyl)phosphonate **8c** (^{31}P NMR 202 MHz, $CDCl_3$).

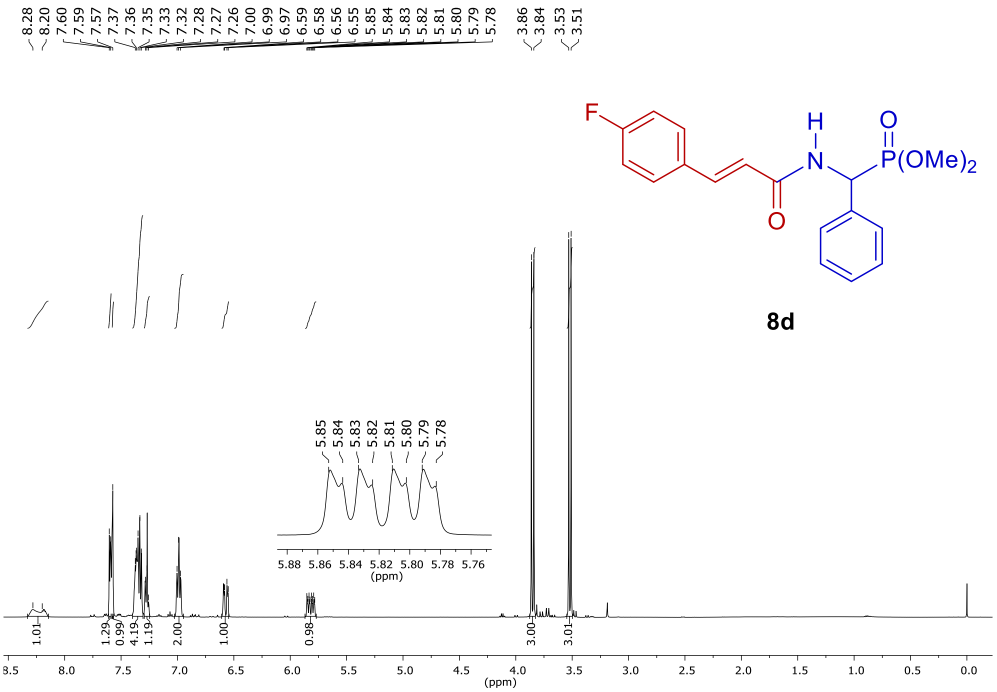

Figure S67. Spectrum of dimethyl *N*-[3-(4-fluorophenyl)-2-ene-1-oxo]-(phenyl-methyl)phosphonate **8d** (^{1}H NMR 500 MHz, $CDCl_3$).

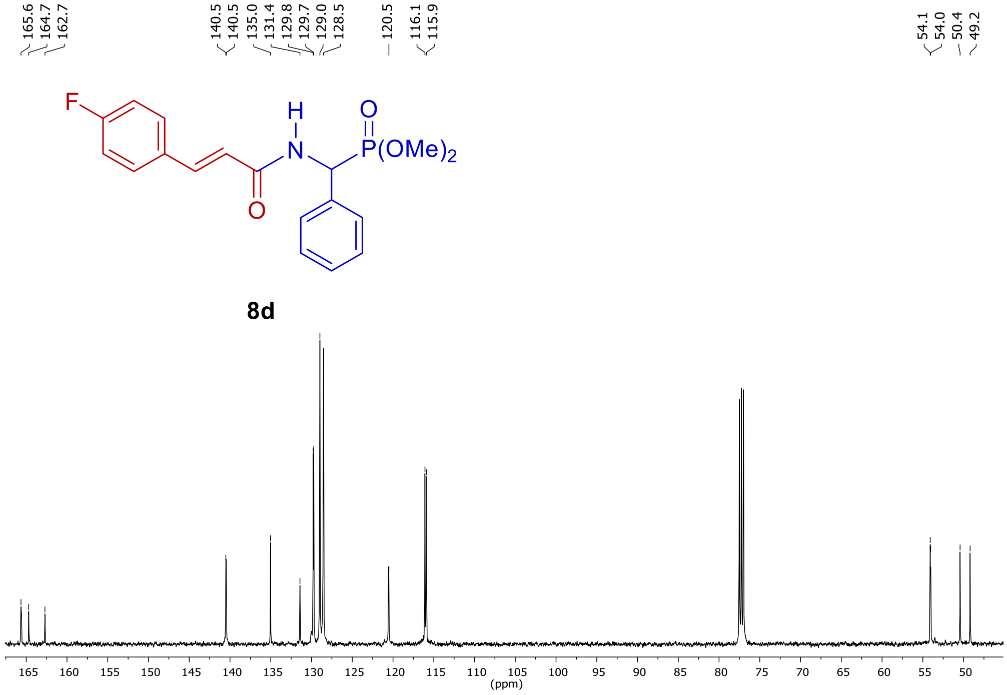

Figure S68. Spectrum of dimethyl *N*-[3-(4-fluorophenyl)-2-ene-1-oxo]-(phenyl-methyl)phosphonate **8d** (^{13}C NMR 125 MHz, $CDCl_3$).

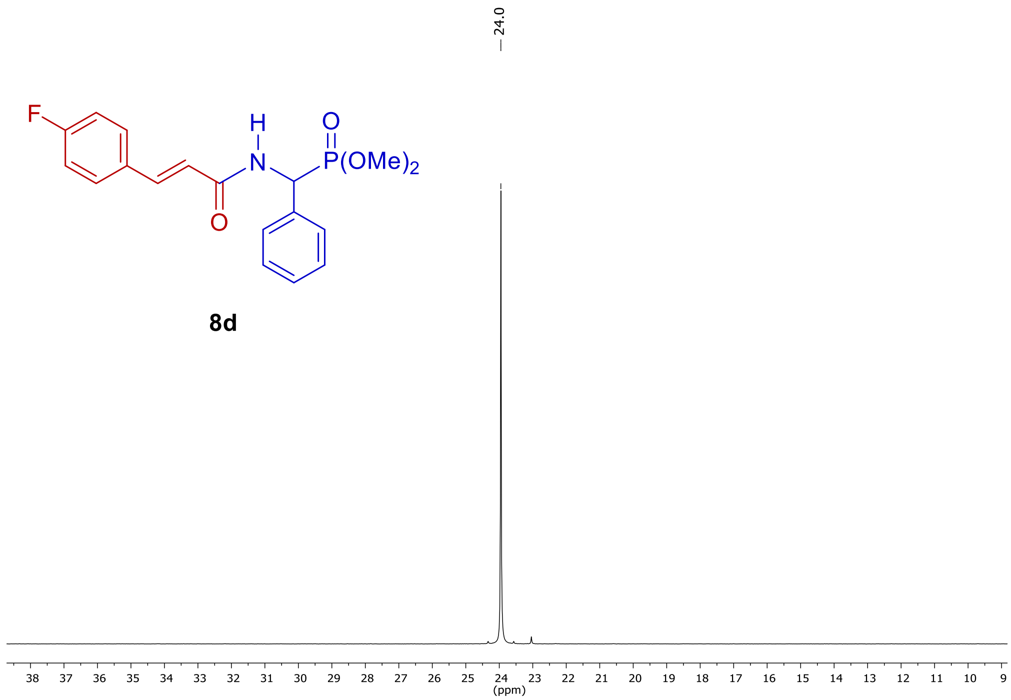

Figure S69. Spectrum of dimethyl *N*-[3-(4-fluorophenyl)-2-ene-1-oxo]-(phenyl-methyl)phosphonate **8d** (^{31}P NMR 202 MHz, $CDCl_3$).

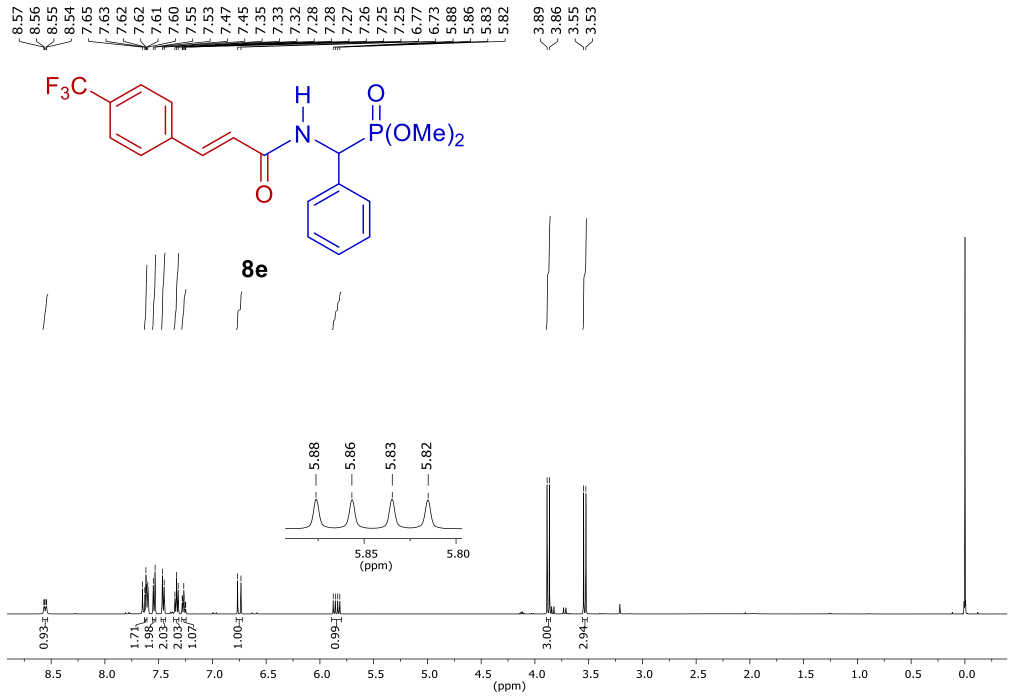

Figure S70. Spectrum of dimethyl *N*-[3-(4-trifluoromethyl)phenyl)-2-ene-1-oxo]-(phenyl-methyl)phosphonate **8e** (^{1}H NMR 500 MHz, $CDCl_3$).

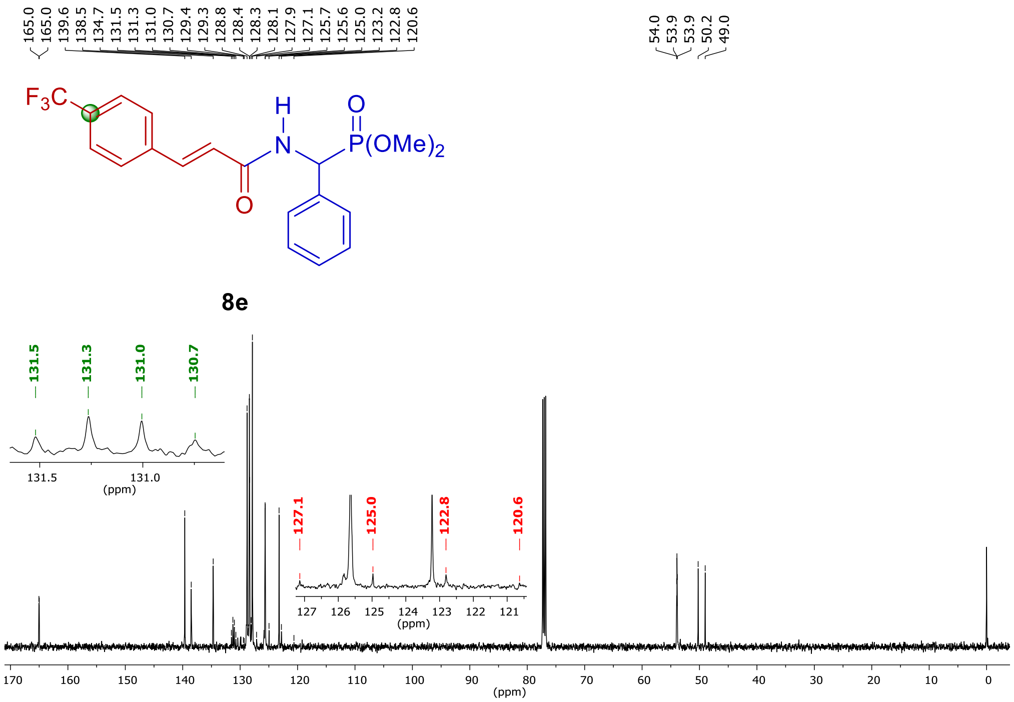

Figure S71. Spectrum of dimethyl *N*-[3-(4-trifluoromethyl)phenyl)-2-ene-1-oxo]-(phenylmethyl)phosphonate **8e** (^{13}C NMR 125 MHz, $CDCl_3$).

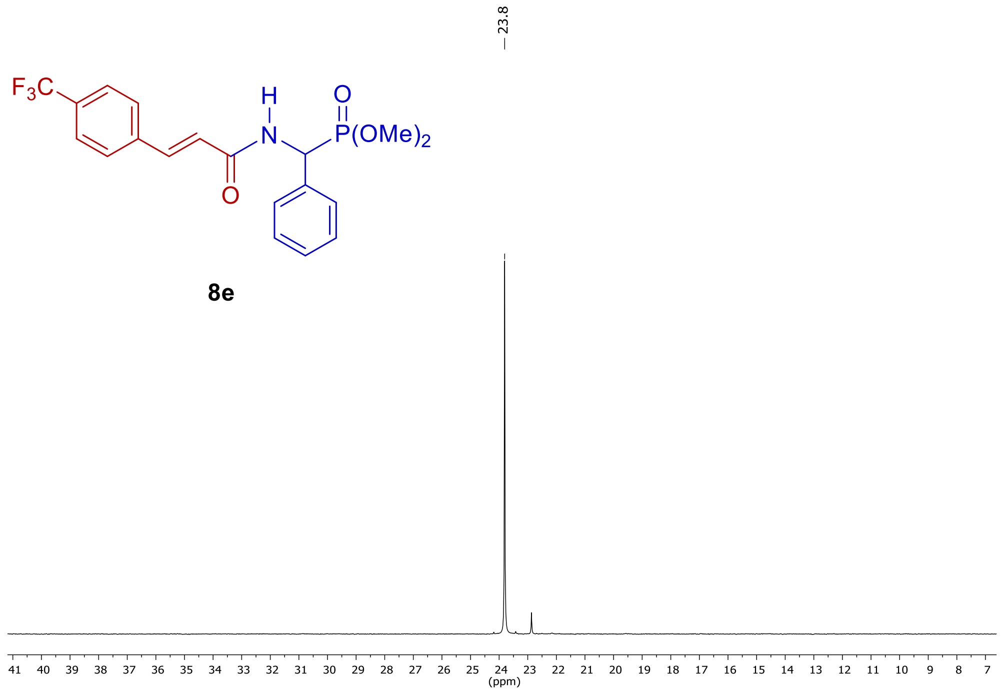

Figure S72. Spectrum of dimethyl *N*-[3-(4-trifluoromethyl)phenyl)-2-ene-1-oxo]-(phenylmethyl)phosphonate **8e** (^{31}P NMR 202 MHz, $CDCl_3$).

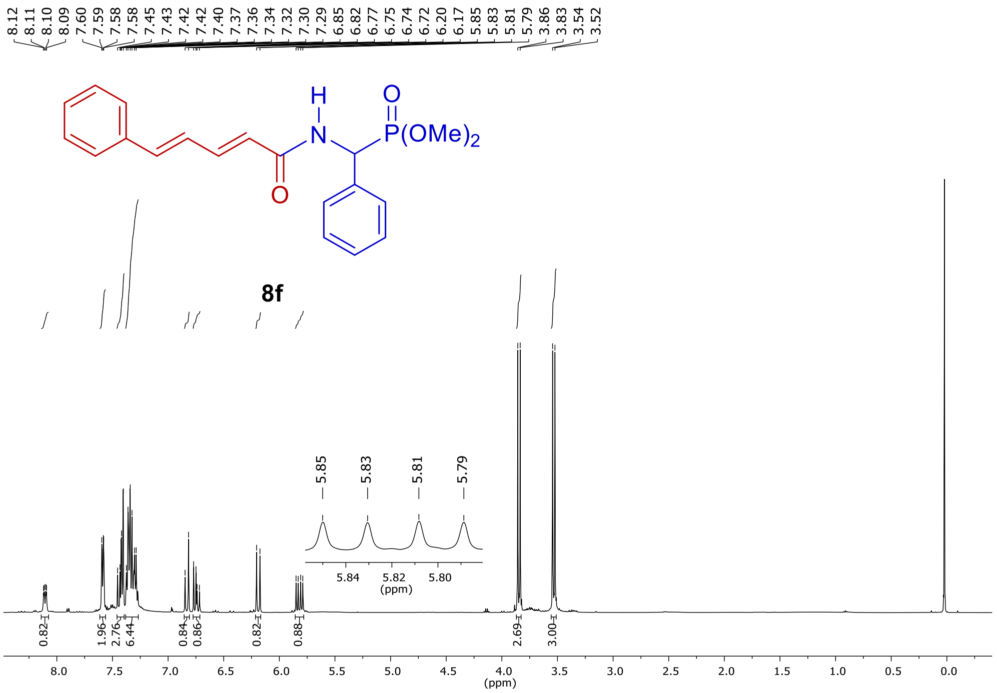

Figure S73. Spectrum of dimethyl *N*-[(2*E*,4*E*)-5-phenylpenta-2,4-dien-1-oxo]-(phenylmethyl)phosphonate **8f** (^{1}H NMR 500 MHz, $CDCl_3$).

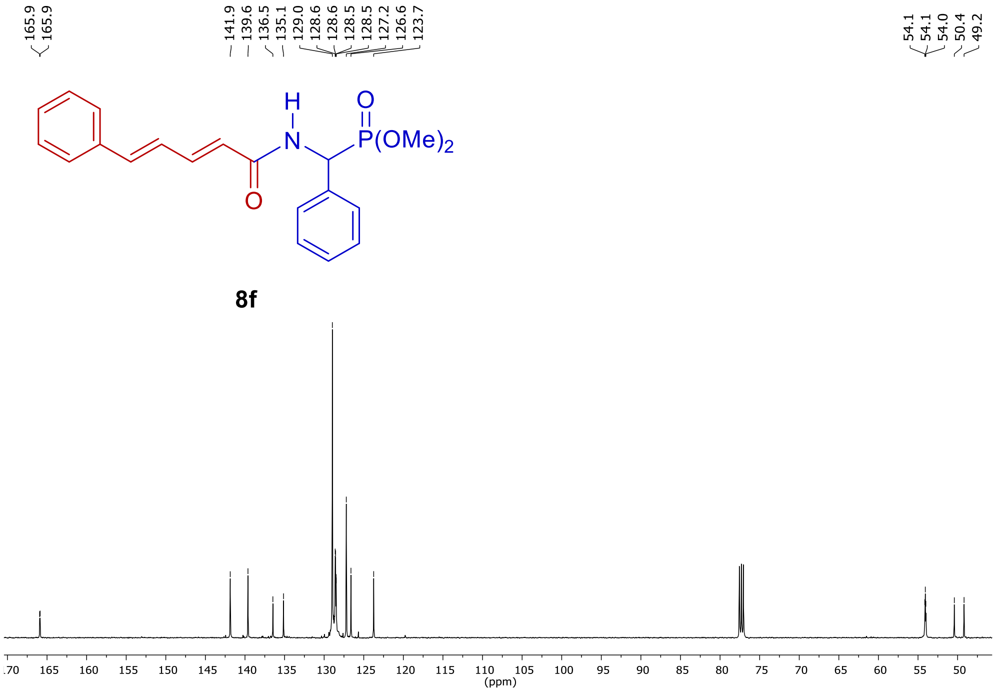

Figure S74. Spectrum of dimethyl *N*-[(2*E*,4*E*)-5-phenylpenta-2,4-dien-1-oxo]-(phenylmethyl)phosphonate **8f** (^{13}C NMR 125 MHz, $CDCl_3$).

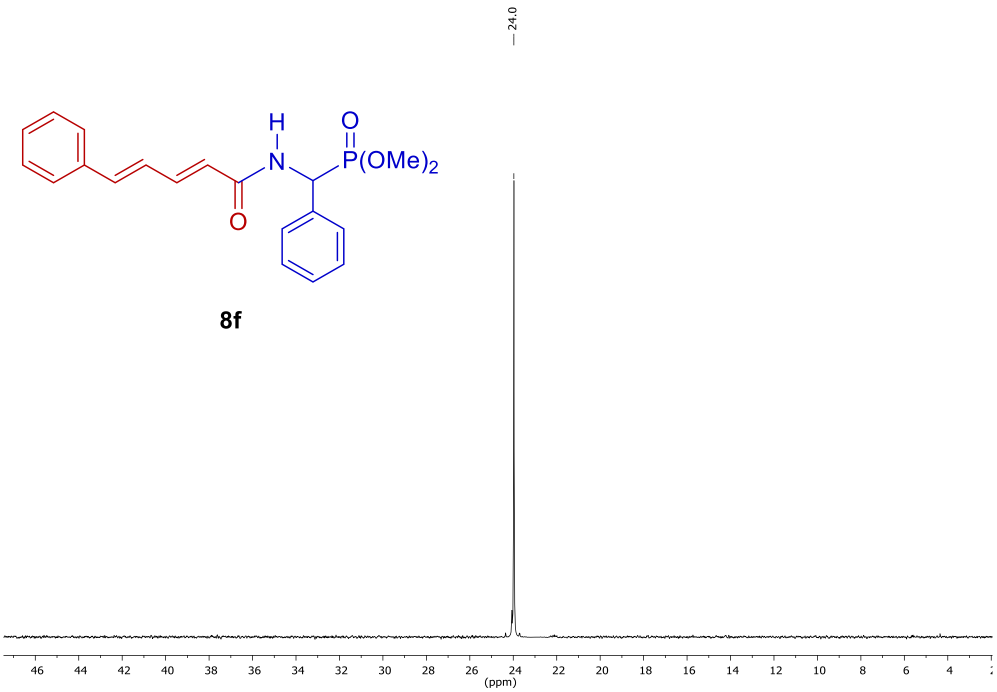

Figure S75. Spectrum of dimethyl *N*-[(2*E*,4*E*)-5-phenylpenta-2,4-dien-1-oxo]-(phenyl-methyl)phosphonate **8f** (^{31}P NMR 202 MHz, $CDCl_3$).

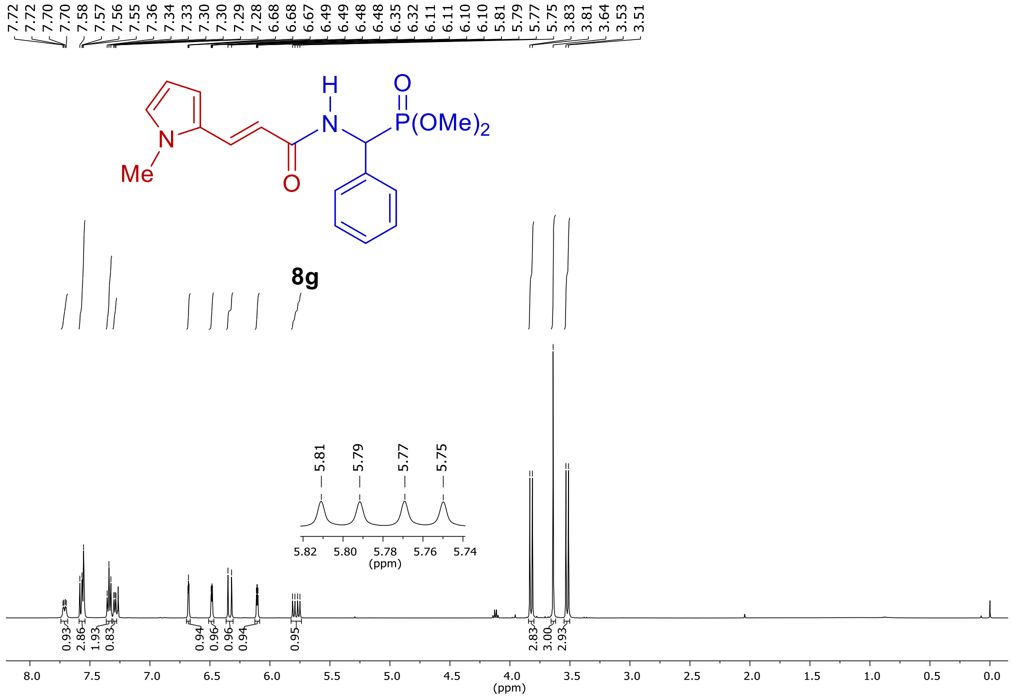

Figure S76. Spectrum of dimethyl *N*-[*N*-methylpyrrol-2-ene-1-oxo]-(phenyl-methyl)phosphonate **8g** (^{1}H NMR 500 MHz, $CDCl_3$).

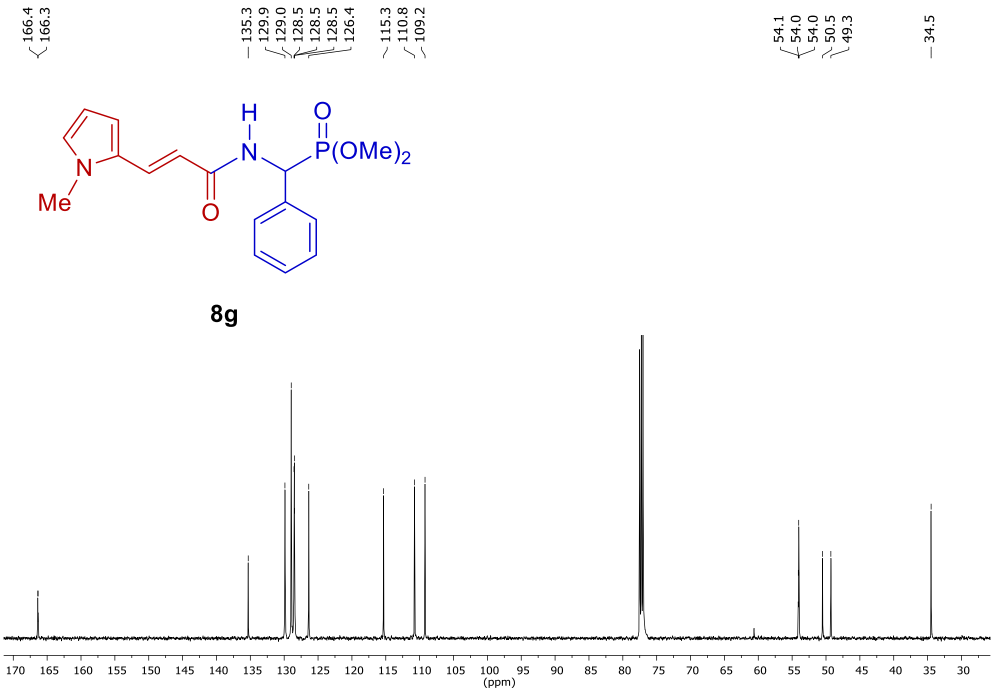

Figure S77. Spectrum of dimethyl *N*-[*N*-methylpyrrol-2-ene-1-oxo]-(phenyl-methyl)phosphonate **8g** (^{13}C NMR 125 MHz, $CDCl_3$).

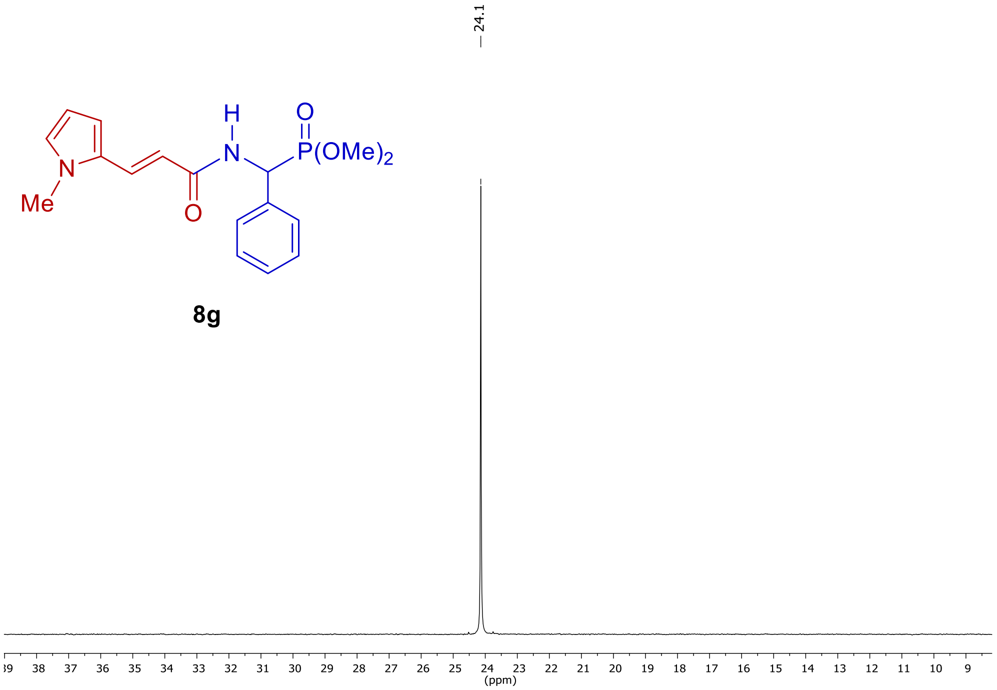

Figure S78. Spectrum of dimethyl *N*-[*N*-methylpyrrol-2-ene-1-oxo]-(phenyl-methyl)phosphonate **8g** (^{31}P NMR 202 MHz, $CDCl_3$).

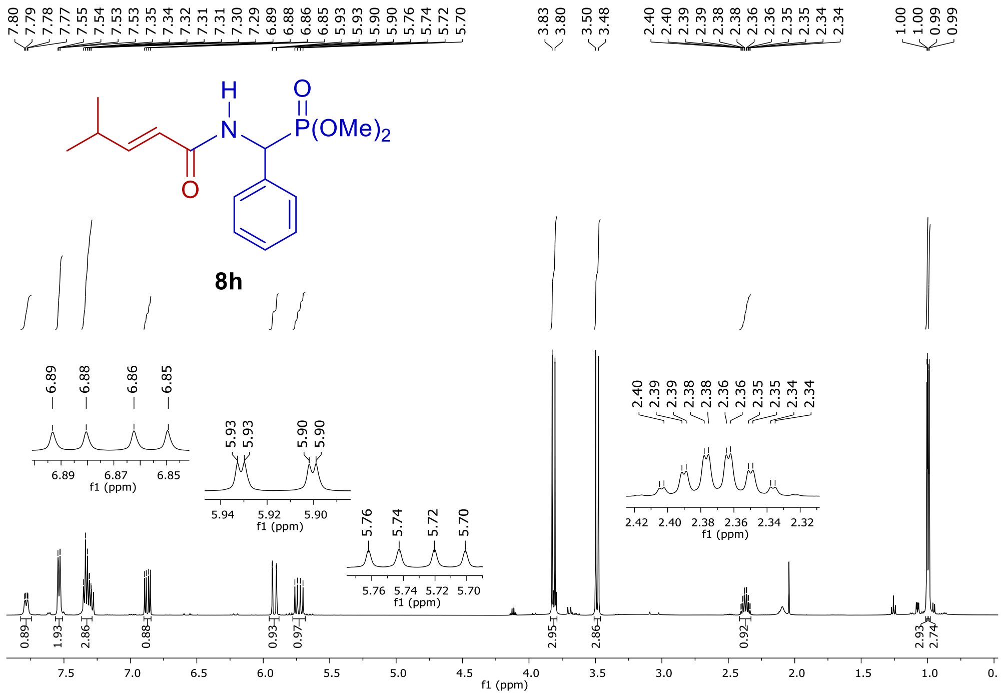

Figure S79. Spectrum of dimethyl *N*-[4-methylpent-2-ene-1-oxo]-(phenyl-methyl)phosphonate **8h** (^{1}H NMR 500 MHz, $CDCl_3$).

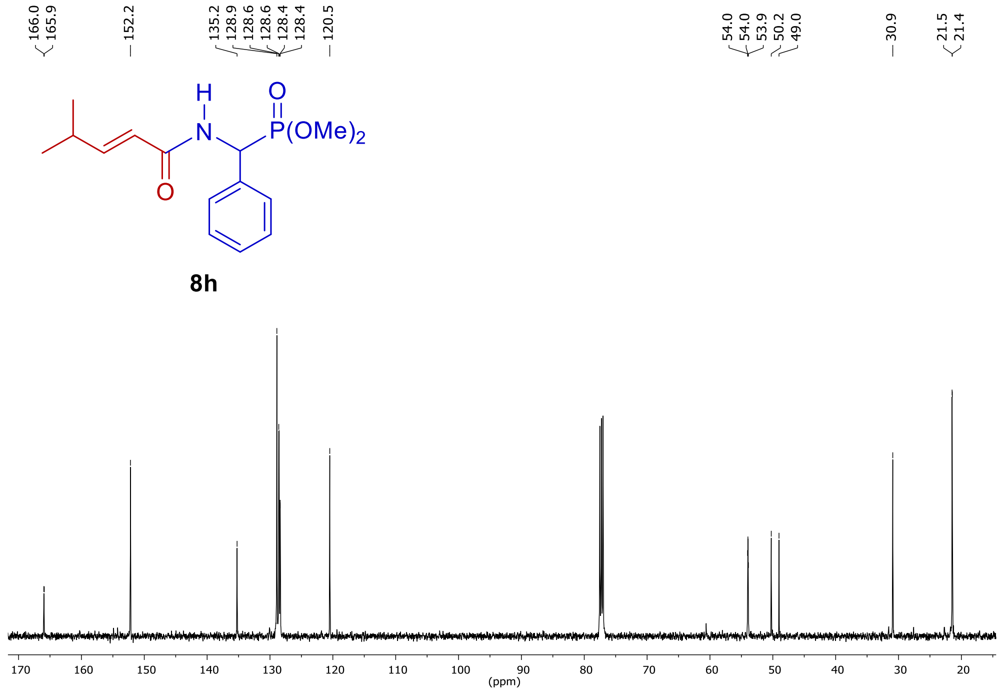

Figure S80. Spectrum of dimethyl *N*-[4-methylpent-2-ene-1-oxo]-(phenyl-methyl)phosphonate **8h** (^{13}C NMR 125 MHz, $CDCl_3$).

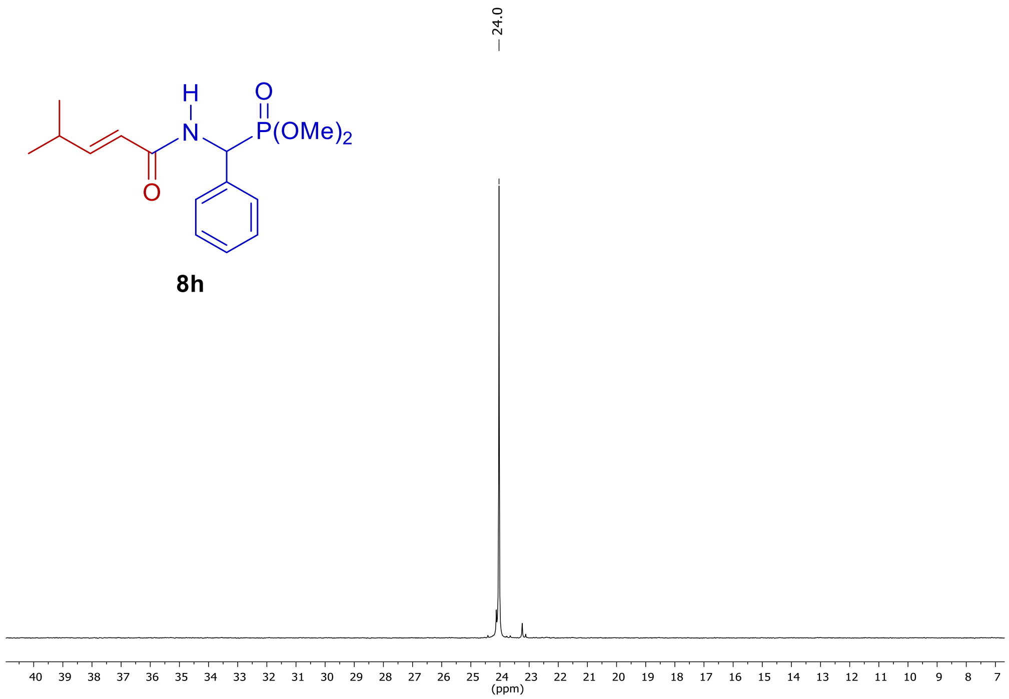

Figure S81. Spectrum of dimethyl *N*-[4-methylpent-2-ene-1-oxo]-(phenyl-methyl)phosphonate **8h** (^{31}P NMR 202 MHz, $CDCl_3$).

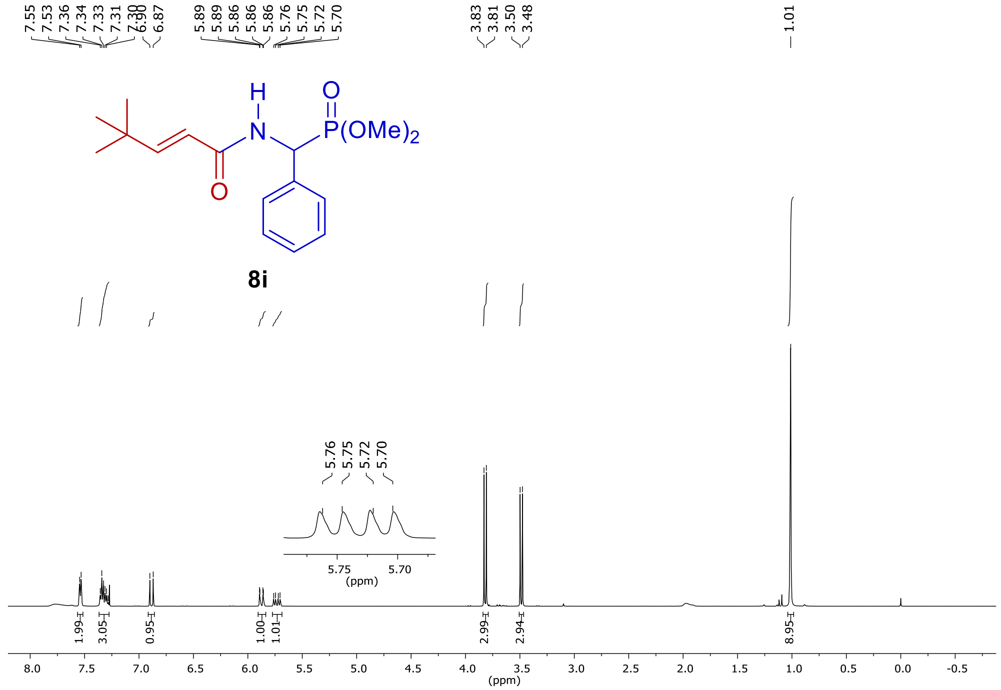

Figure S82. Spectrum of dimethyl *N*-[4,4-dimethylpent-2-ene-1-oxo]-(phenyl-methyl)phosphonate **8i** (^{1}H NMR 500 MHz, $CDCl_3$).

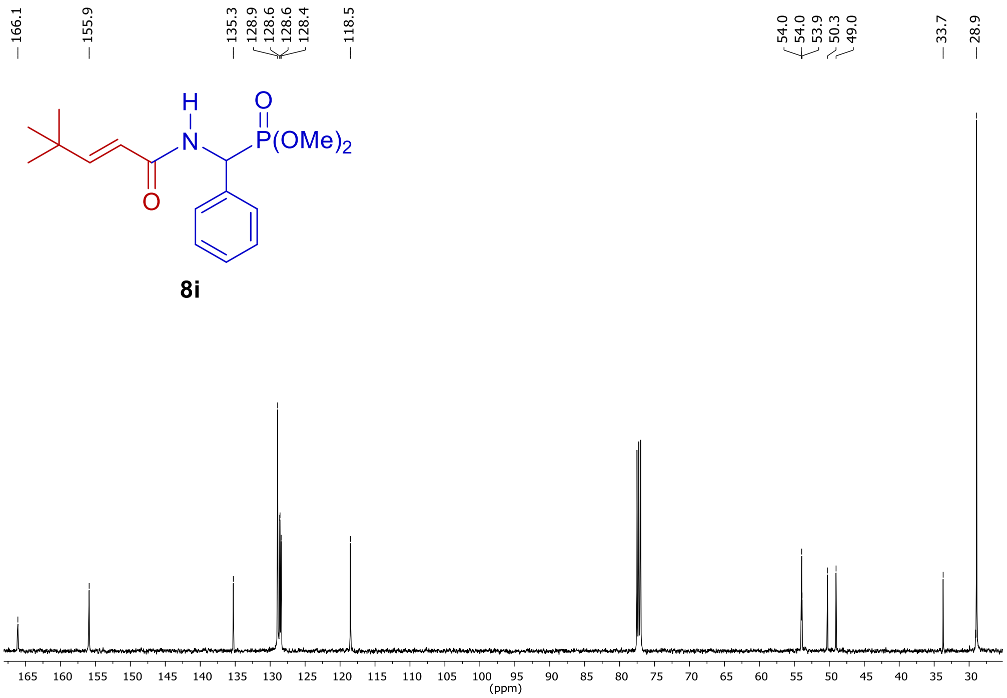

Figure S83. Spectrum of dimethyl *N*-[4,4-dimethylpent-2-ene-1-oxo]-(phenyl-methyl)phosphonate **8i** (^{13}C NMR 125 MHz, $CDCl_3$).

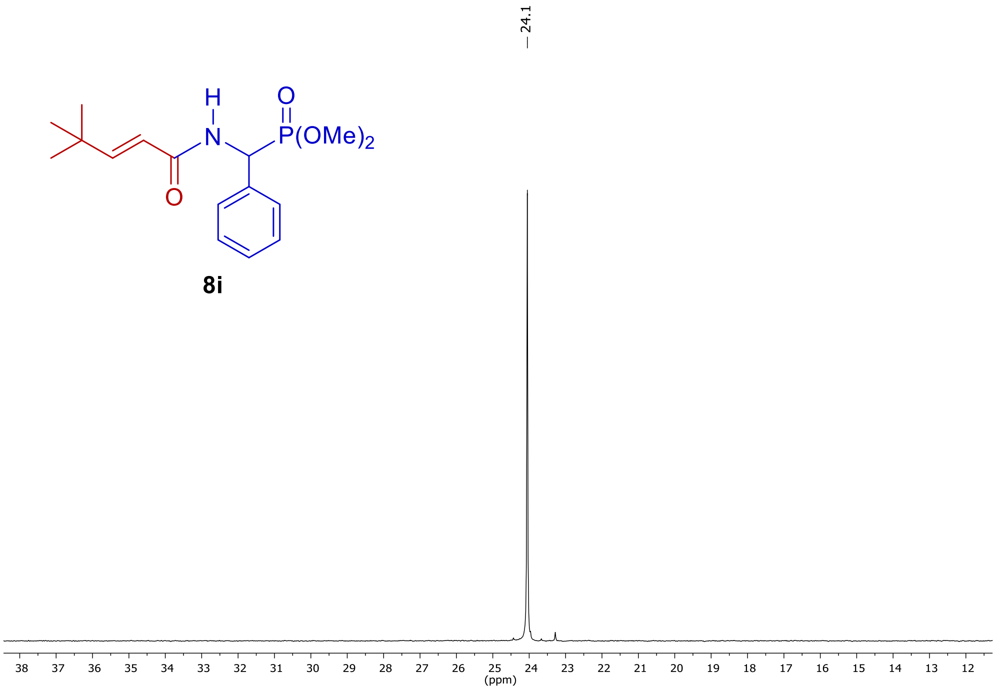

Figure S84. Spectrum of dimethyl *N*-[4,4-dimethylpent-2-ene-1-oxo]-(phenyl-methyl)phosphonate **8i** (^{31}P NMR 202 MHz, $CDCl_3$).

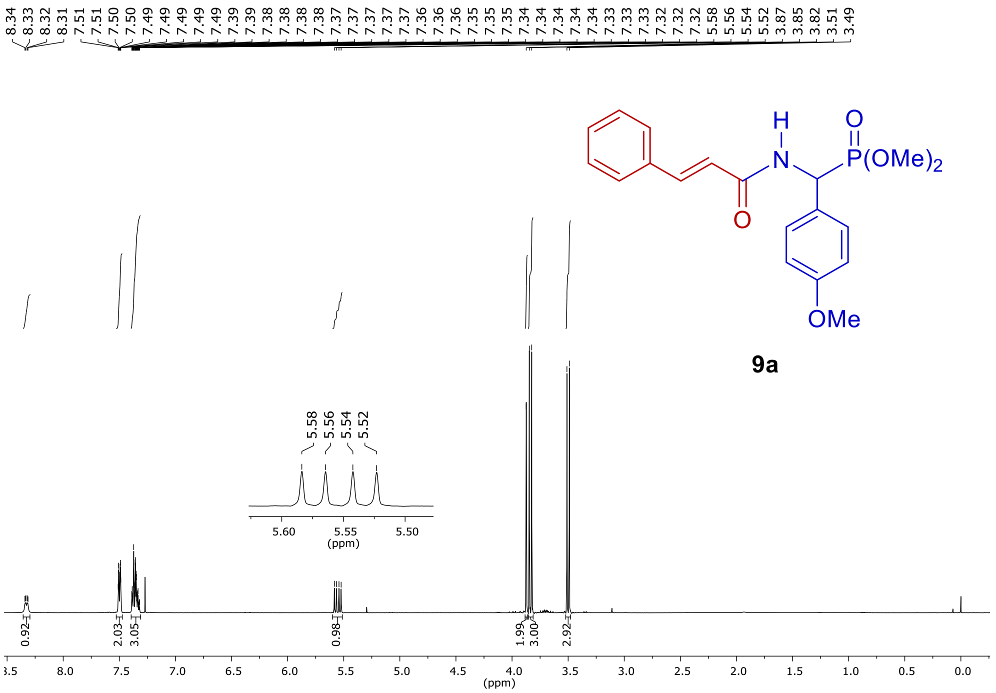

Figure S85. Spectrum of dimethyl *N*-[3-phenyl-2-ene-1-oxo]-(4-methoxyphenyl-methyl)phosphonate **9a** (^{1}H NMR 500 MHz, $CDCl_3$).

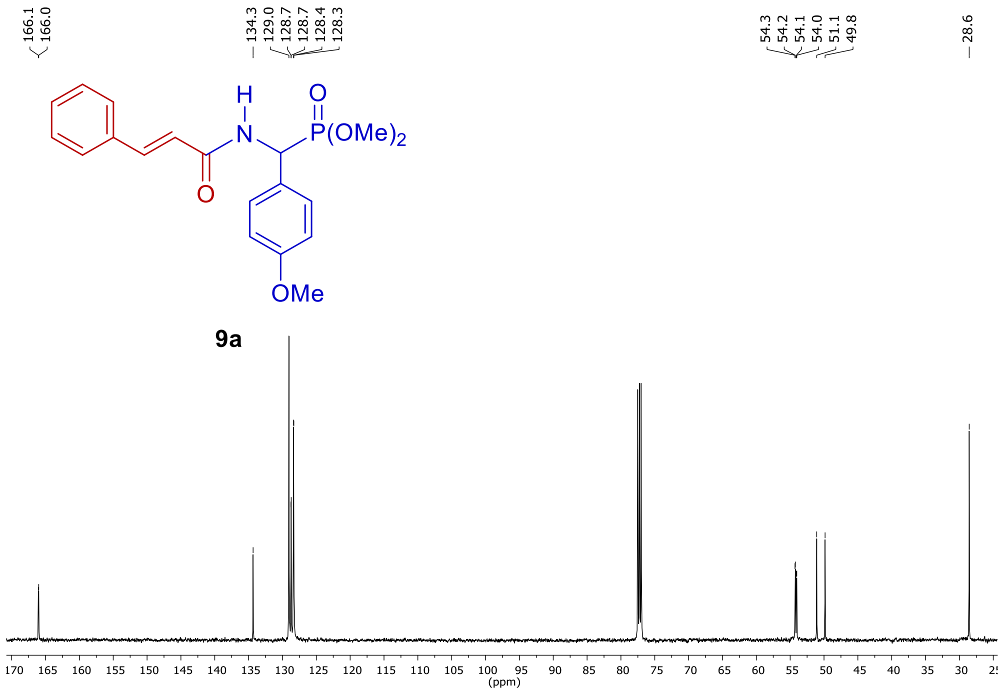

Figure S86. Spectrum of dimethyl *N*-[3-phenyl-2-ene-1-oxo]-(4-methoxyphenyl-methyl)phosphonate **9a** (^{13}C NMR 125 MHz, $CDCl_3$).

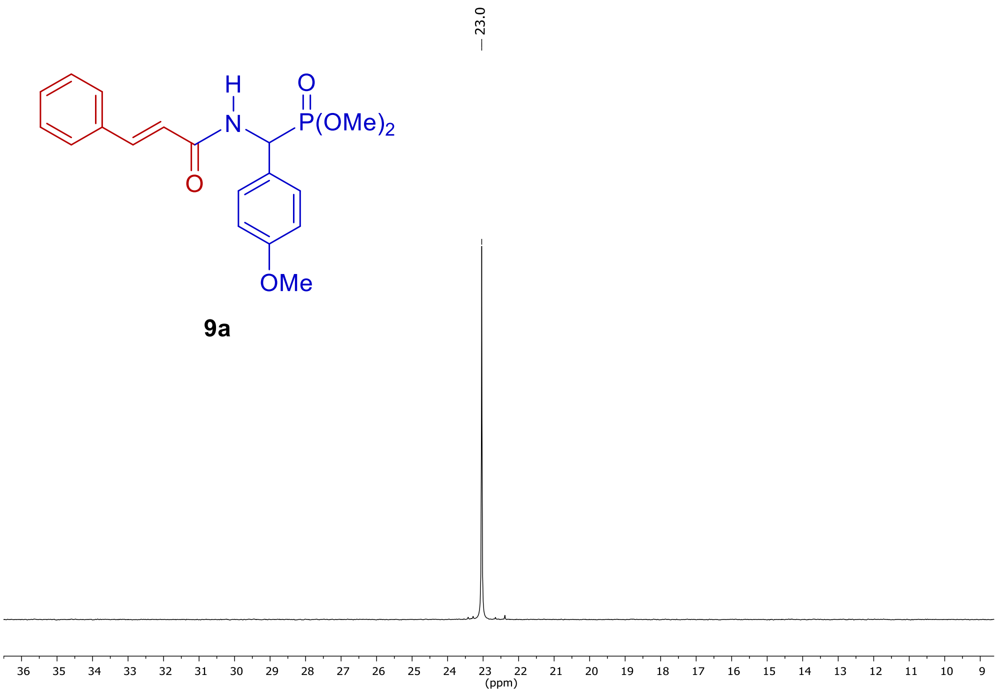

Figure S87. Spectrum of dimethyl *N*-[3-phenyl-2-ene-1-oxo]-(4-methoxyphenyl-methyl)phosphonate **9a** (^{31}P NMR 202 MHz, $CDCl_3$).

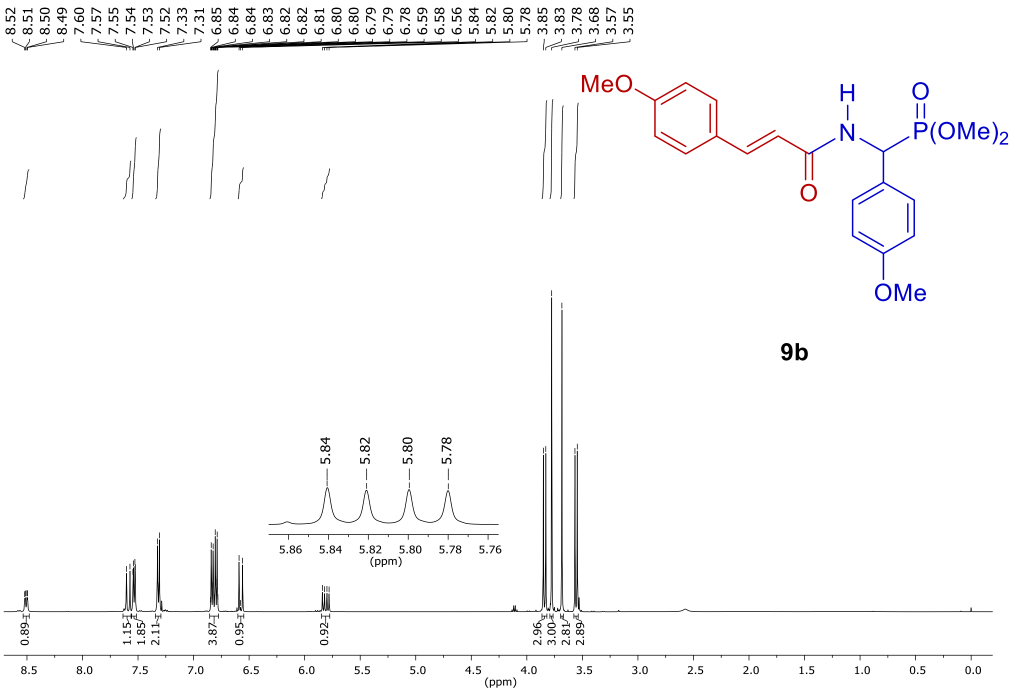

Figure S88. Spectrum of dimethyl *N*-[3-(4-methoxyphenyl)-2-ene-1-oxo]-(4-methoxyphenyl-methyl)phosphonate **9b** (^{1}H NMR 500 MHz, $CDCl_3$).

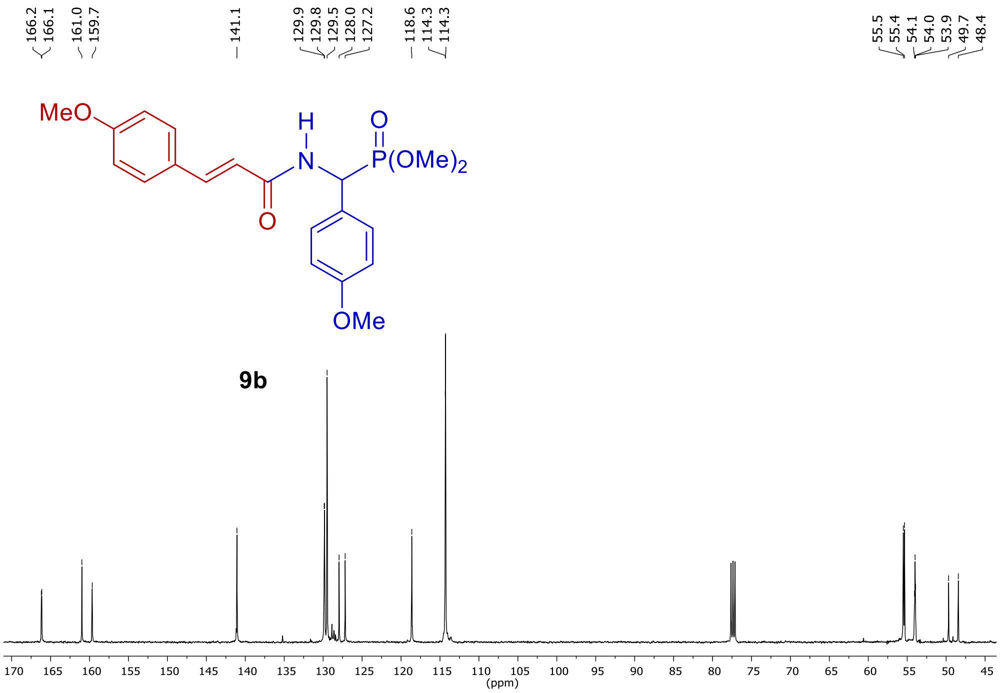

Figure S89. Spectrum of dimethyl *N*-[3-(4-methoxyphenyl)-2-ene-1-oxo]-(4-methoxyphenyl-methyl)phosphonate **9b** (^{13}C NMR 125 MHz, $CDCl_3$).

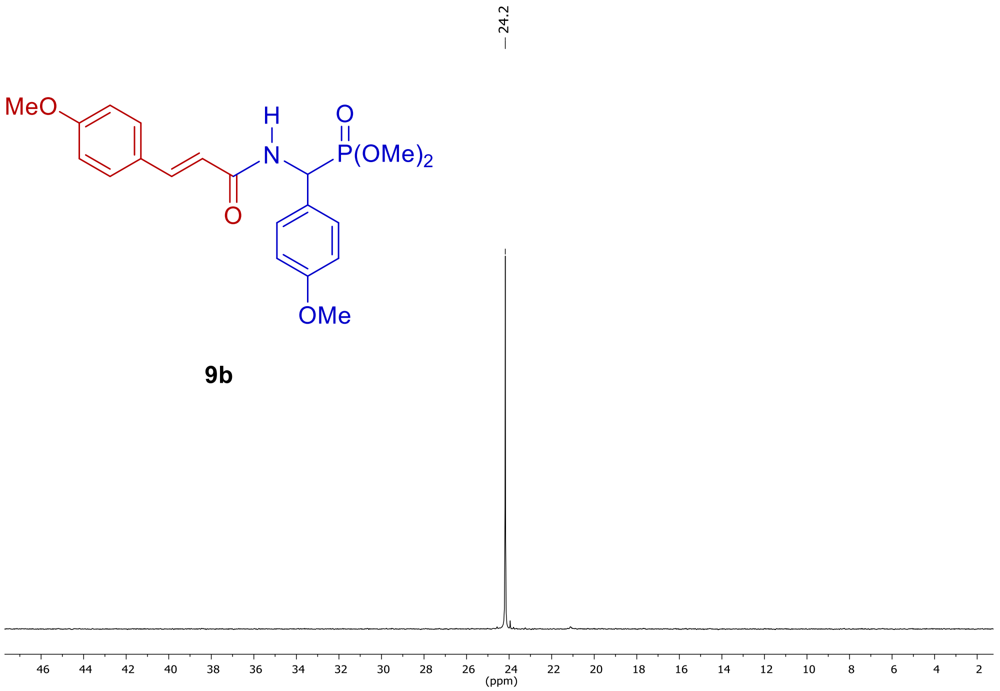

Figure S90. Spectrum of dimethyl *N*-[3-(4-methoxyphenyl)-2-ene-1-oxo]-(4-methoxyphenyl-methyl)phosphonate **9b** (^{31}P NMR 202 MHz, $CDCl_3$).

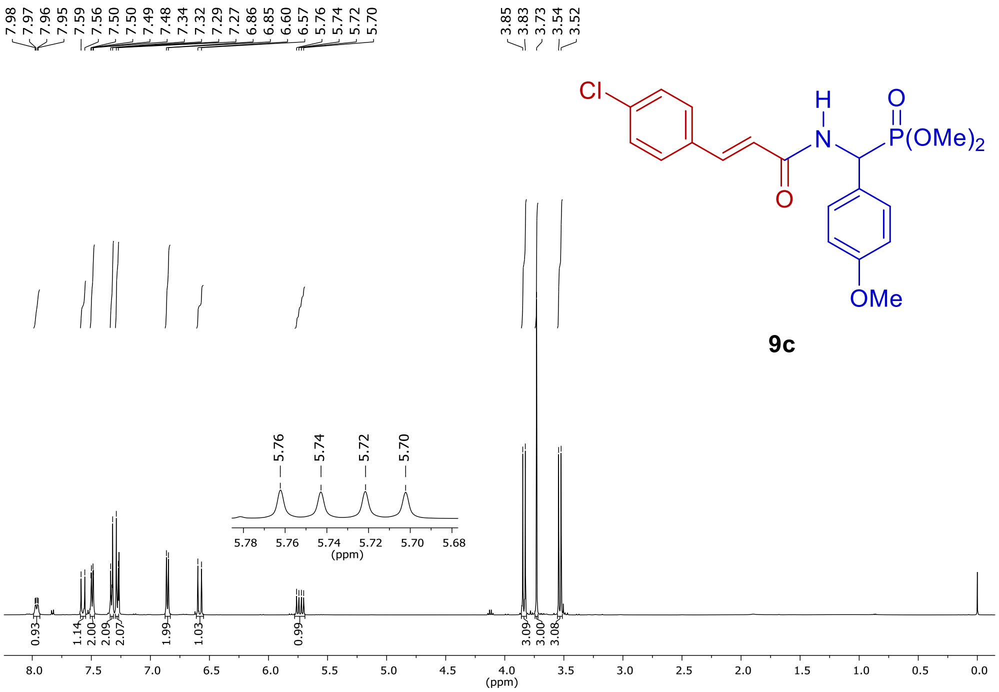

Figure S91. Spectrum of dimethyl *N*-[3-(4-chlorophenyl)-2-ene-1-oxo]-(4-methoxyphenyl-methyl)phosphonate **9c** (^{1}H NMR 500 MHz, $CDCl_3$).

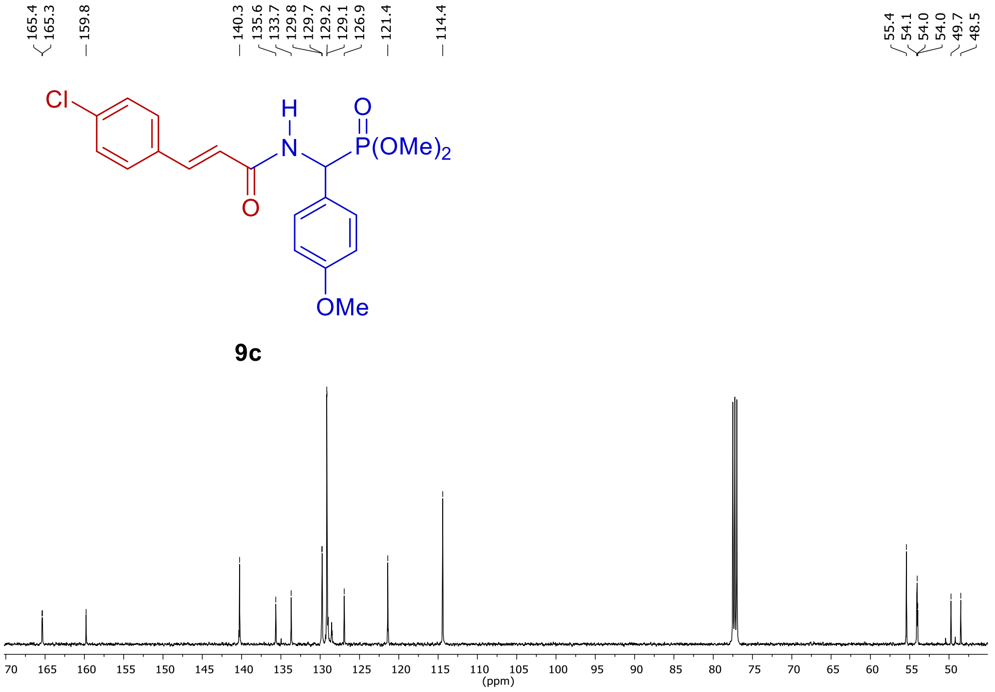

Figure S92. Spectrum of dimethyl *N*-[3-(4-chlorophenyl)-2-ene-1-oxo]-(4-methoxyphenyl-methyl)phosphonate **9c** (^{13}C NMR 125 MHz, $CDCl_3$).

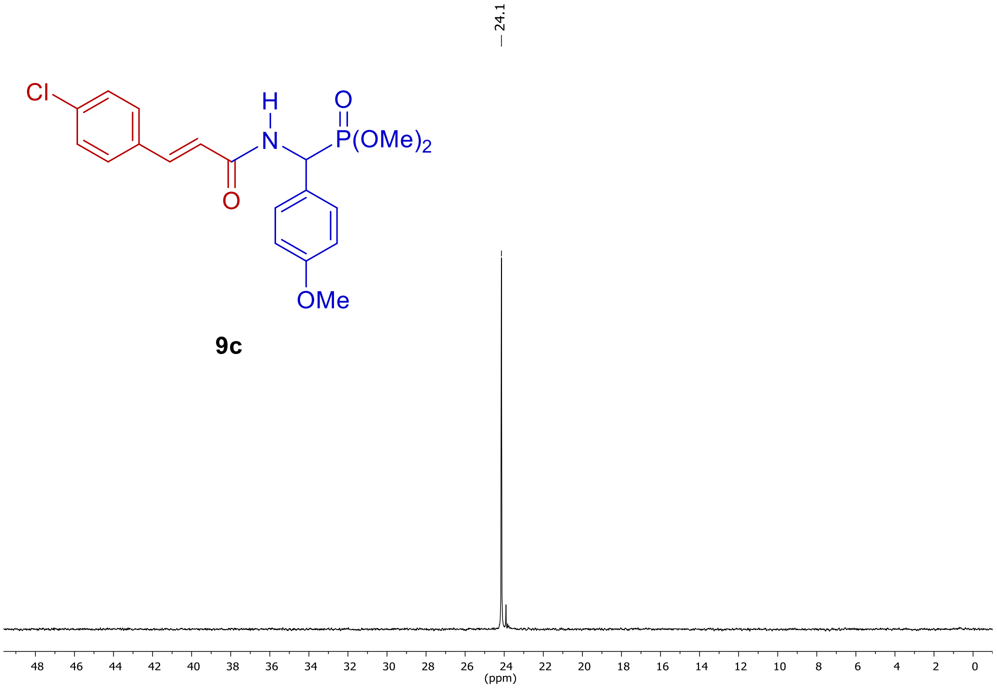

Figure S93. Spectrum of dimethyl *N*-[3-(4-chlorophenyl)-2-ene-1-oxo]-(4-methoxyphenyl-methyl)phosphonate **9c** (^{31}P NMR 202 MHz, $CDCl_3$).

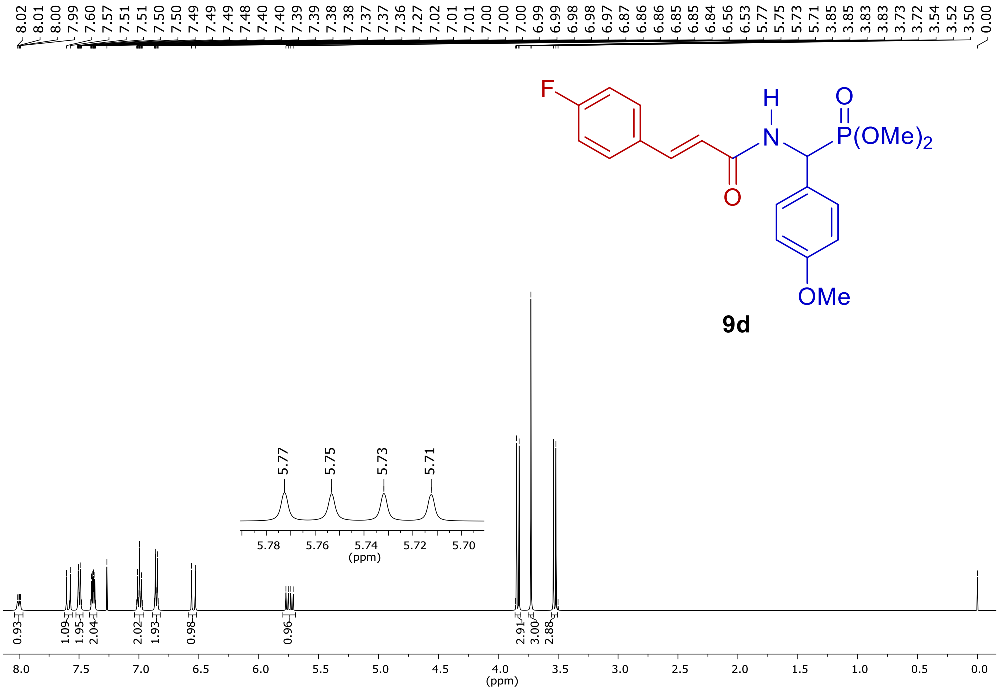

Figure S94. Spectrum of dimethyl *N*-[3-(4-fluorophenyl)-2-ene-1-oxo]-(4-methoxyphenyl-methyl)phosphonate **9d (**^{1}H NMR (500 MHz, $CDCl_3$).

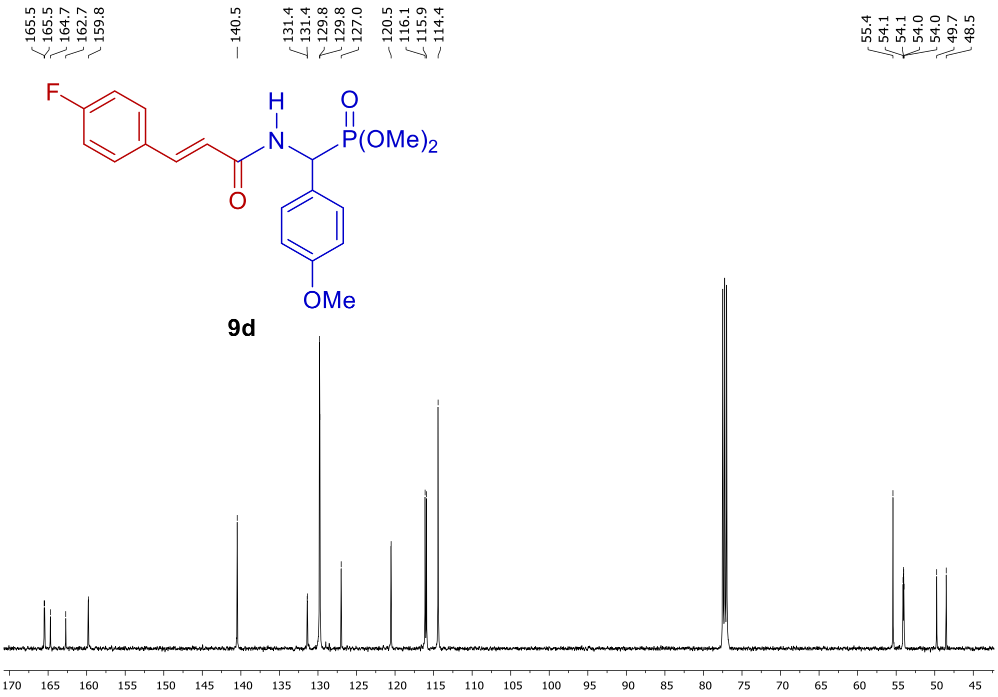

Figure S95. Spectrum of dimethyl *N*-[3-(4-fluorophenyl)-2-ene-1-oxo]-(4-methoxyphenyl-methyl)phosphonate **9d** (^{13}C NMR 125 MHz, $CDCl_3$).

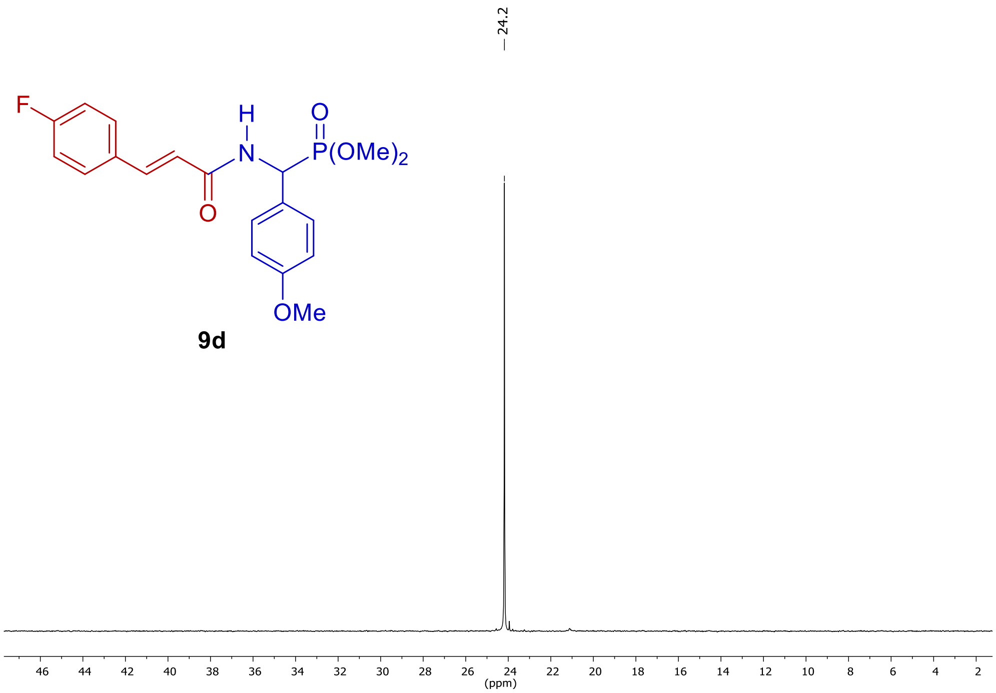

Figure S96. Spectrum of dimethyl *N*-[3-(4-fluorophenyl)-2-ene-1-oxo]-(4-methoxyphenyl-methyl)phosphonate **9d** (^{31}P NMR 202 MHz, $CDCl_3$).

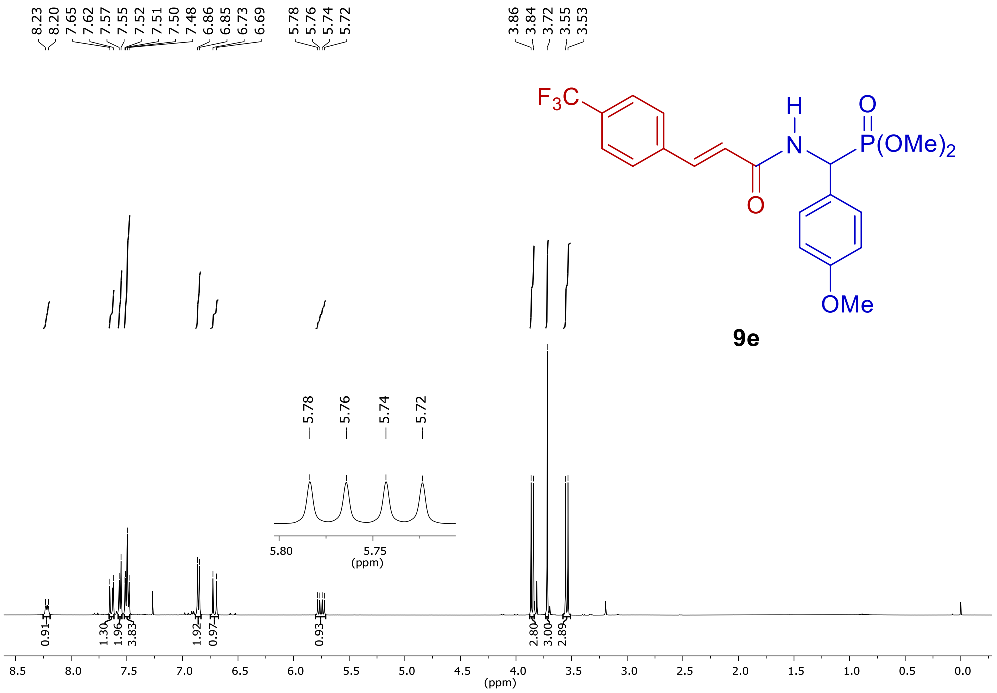

Figure S97. Spectrum of dimethyl *N*-[3-(4-(trifluoromethyl)phenyl)-2-ene-1-oxo]-(4-methoxyphenyl-methyl)phosphonate **9e** (^{1}H NMR 500 MHz, $CDCl_3$).

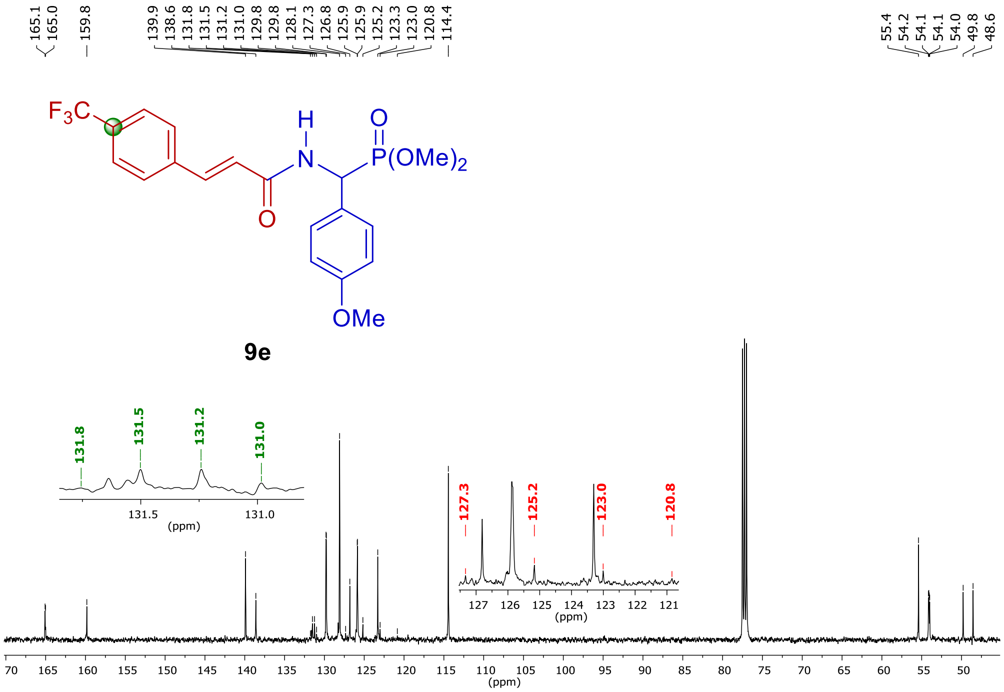

Figure S98. Spectrum of dimethyl *N*-[3-(4-(trifluoromethyl)phenyl)-2-ene-1-oxo]-(4-methoxyphenyl-methyl)phosphonate **9e** (^{13}C NMR 125 MHz, $CDCl_3$).

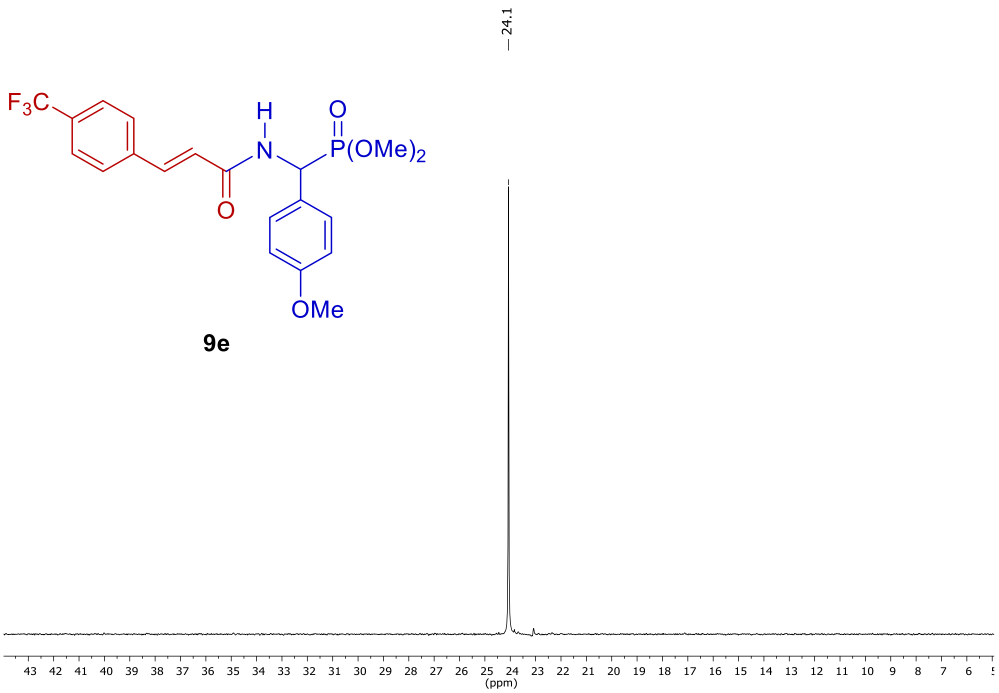

Figure S99. Spectrum of dimethyl *N*-[3-(4-(trifluoromethyl)phenyl)-2-ene-1-oxo]-(4-methoxyphenyl-methyl)phosphonate **9e** (^{31}P NMR 202 MHz, $CDCl_3$).

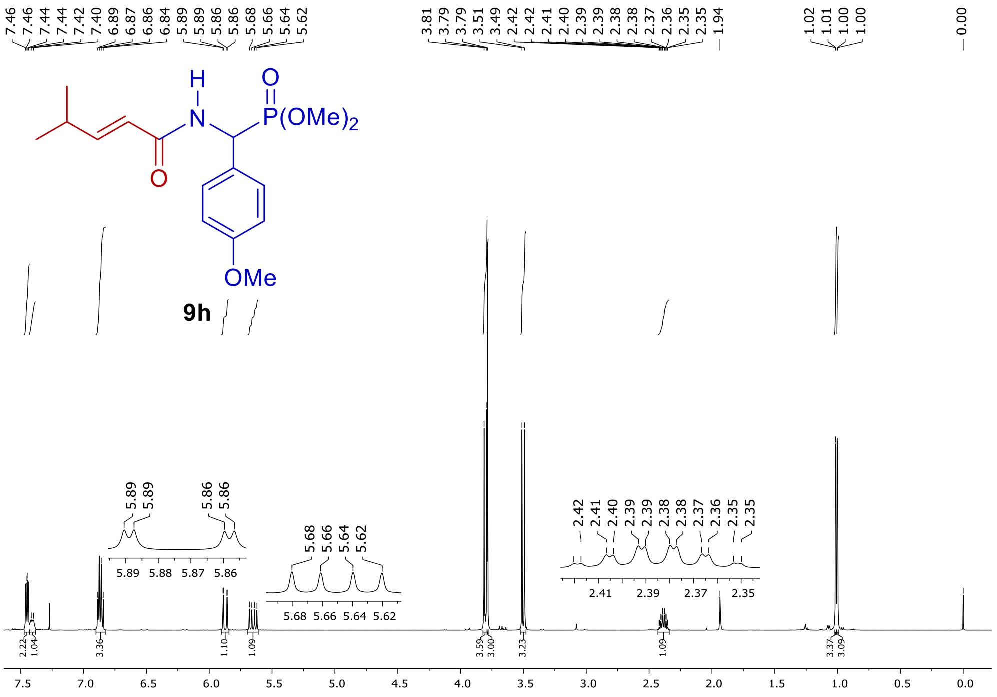

Figure S100. Spectrum of dimethyl *N*-[4-methylpent-2-ene-1-oxo]-(4-methoxyphenyl-methyl)phosphonate **9h** (^{1}H NMR 500 MHz, $CDCl_3$).

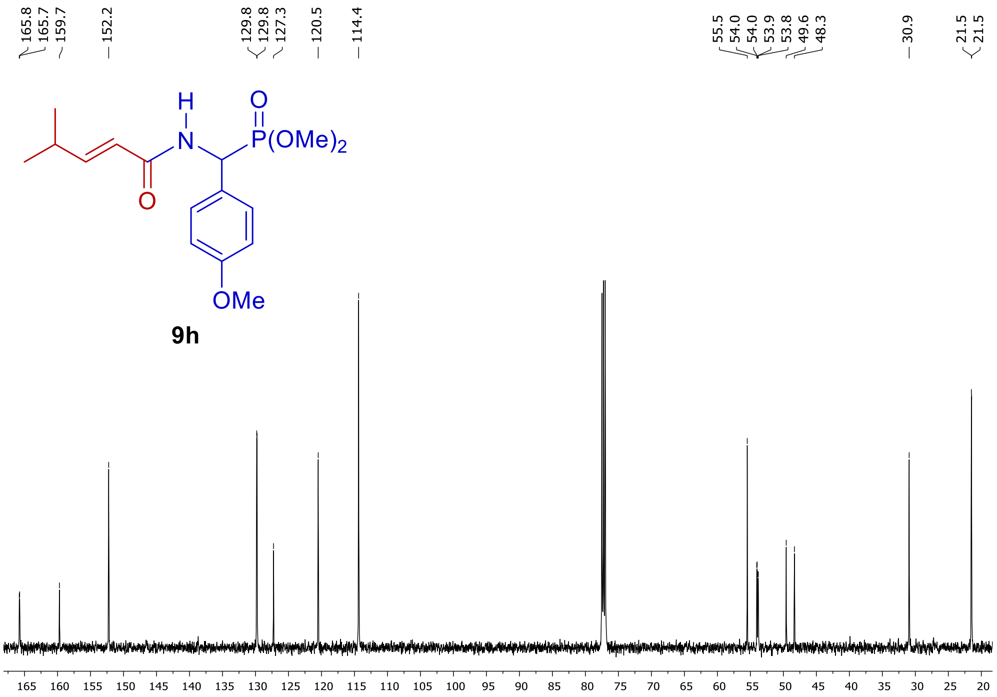

Figure S101. Spectrum of dimethyl *N*-[4-methylpent-2-ene-1-oxo]-(4-methoxyphenyl-methyl)phosphonate **9h** (^{13}C NMR 125 MHz, $CDCl_3$).

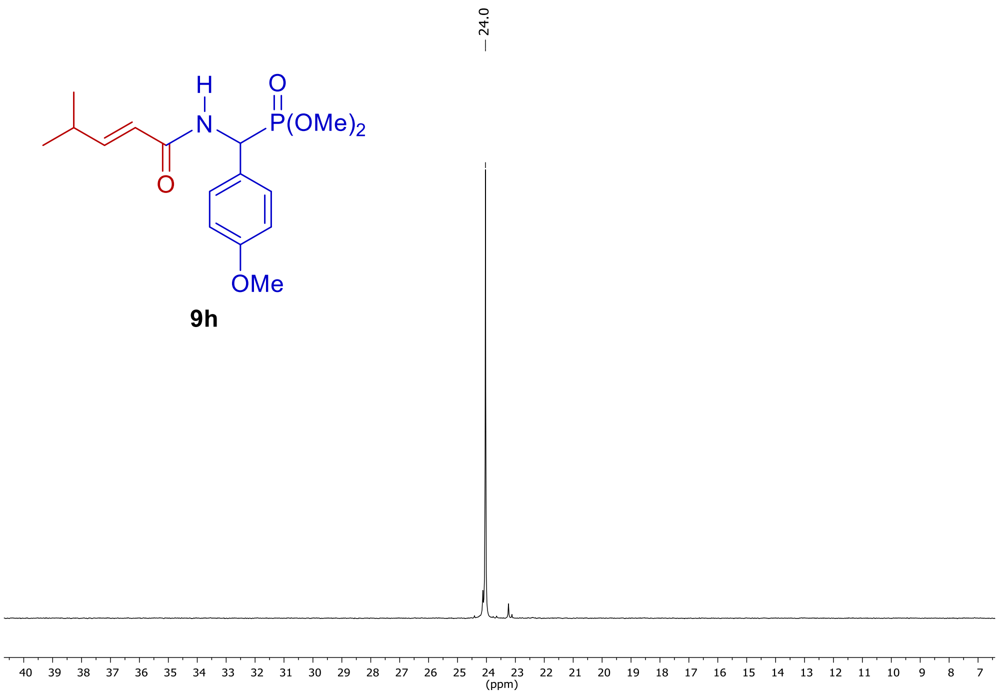

Figure S102. Spectrum of dimethyl *N*-[4-methylpent-2-ene-1-oxo]-(4-methoxyphenyl-methyl)phosphonate **9h** (^{31}P NMR 202 MHz, $CDCl_3$).

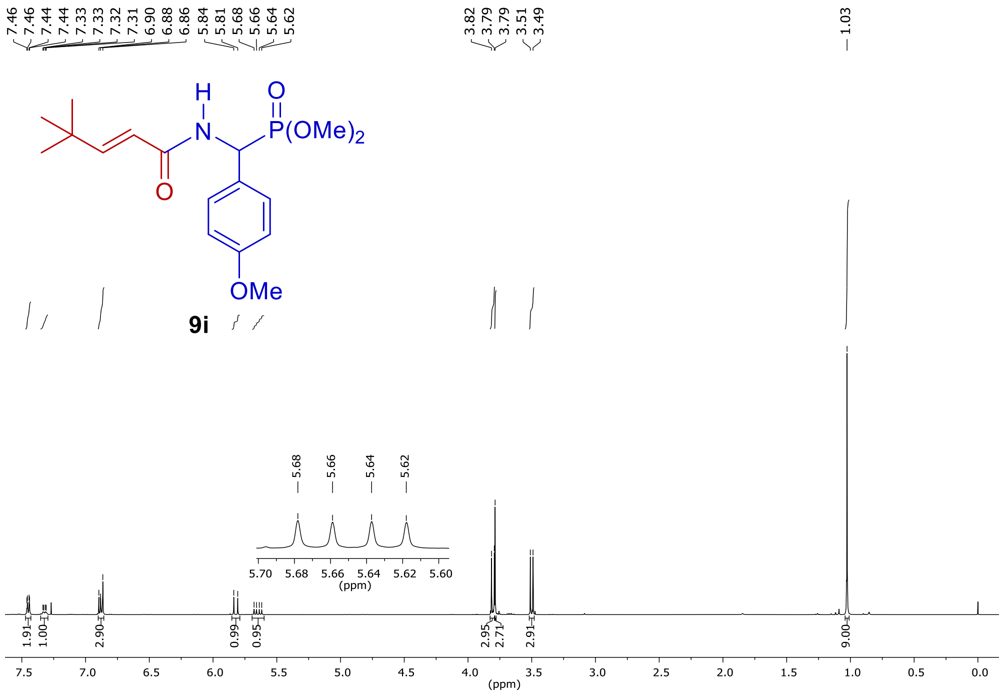

Figure S103. Spectrum of dimethyl *N*-[4,4-dimethylpent-2-ene-1-oxo]-(4-methoxyphenyl-methyl)phosphonate **9i** (^{1}H NMR 500 MHz, $CDCl_3$).

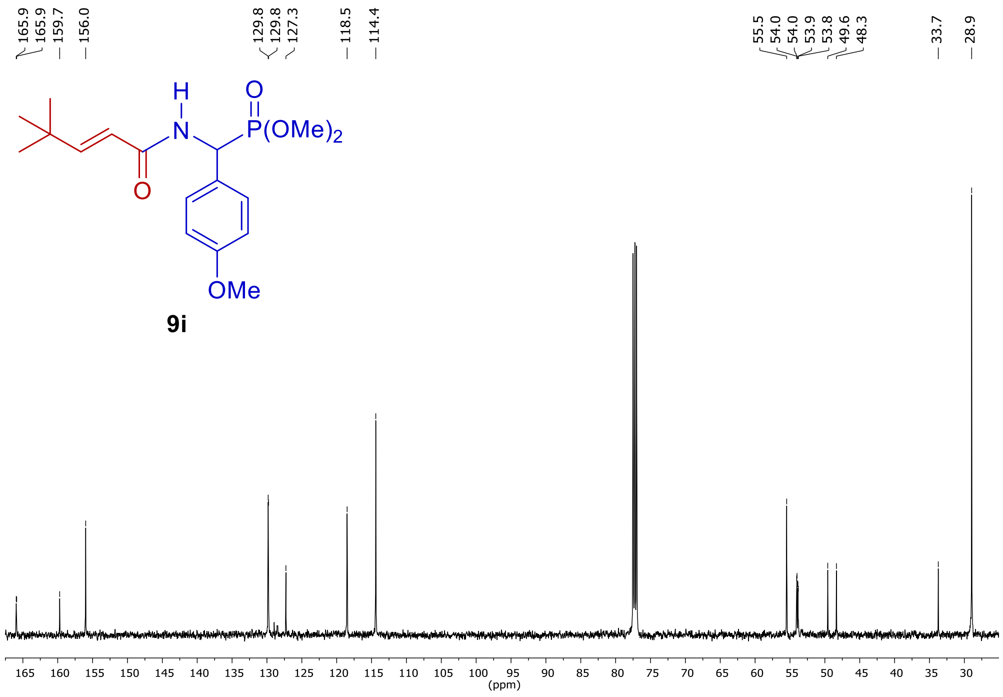

Figure S104. Spectrum of dimethyl *N*-[4,4-dimethylpent-2-ene-1-oxo]-(4-methoxyphenyl-methyl)phosphonate **9i** (^{13}C NMR 125 MHz, $CDCl_3$).

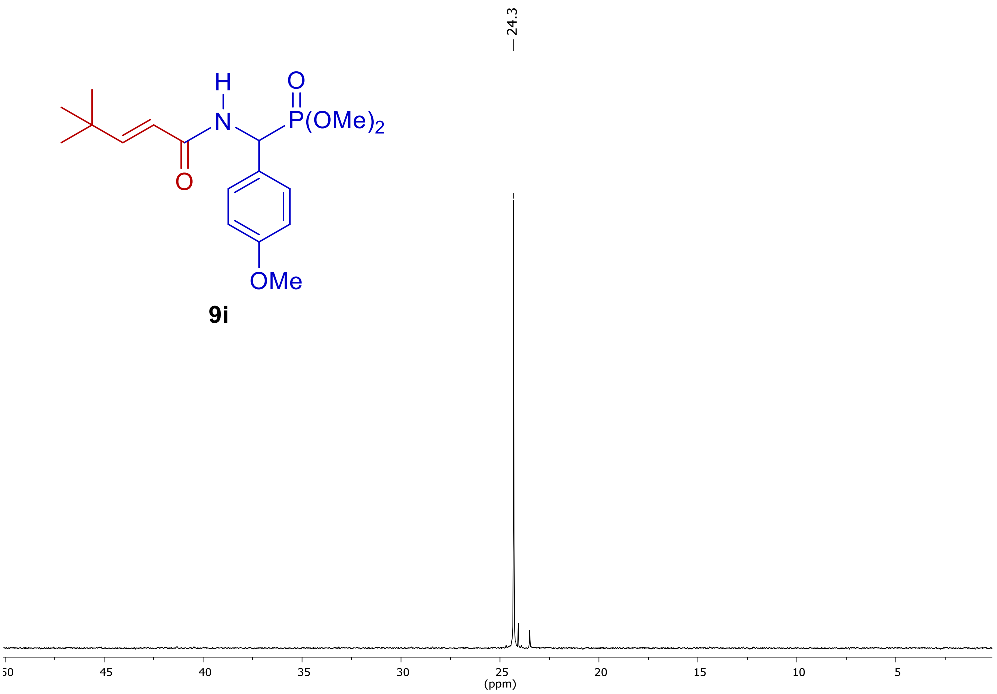

Figure S105. Spectrum of dimethyl *N*-[4,4-dimethylpent-2-ene-1-oxo]-(4-methoxyphenyl-methyl)phosphonate **9i** (^{31}P NMR 202 MHz, $CDCl_3$).
